# Morphological and phylogenetic analyses reveal taxonomic insights in *Marasmiaceae*, including ten new species from Northeast China Ecological Region

**DOI:** 10.3897/imafungus.17.206155

**Published:** 2026-11-05

**Authors:** Wei-Nan Hou, Tolgor Bau

**Affiliations:** 1 College of Mycology, Jilin Agricultural University, Changchun 130118, China College of Mycology, Jilin Agricultural University Changchun China; 2 Key Laboratory of Edible Fungal Resources and Utilization (North), Ministry of Agriculture and Rural Affairs, Jilin Agricultural University, Changchun 130118, China Key Laboratory of Edible Fungal Resources and Utilization (North), Ministry of Agriculture and Rural Affairs, Jilin Agricultural University Changchun China

**Keywords:** Marasmioid fungi, molecular phylogeny, polyphasic taxonomy, saprotrophic fungi

## Abstract

Based on morphological characteristics and phylogenetic analyses using ITS, nrLSU, *rpb*2, and *tef*1-α sequence data, six new species belonging to subg. *Globulares* were identified and described. In addition, this study addressed the taxonomic confusion surrounding ser. *Haematocephali* through detailed discussions. Two new species were documented in *Marasmius* subg. *Marasmius*. Furthermore, Cr. nigricaulis var. macrospora was raised to species rank as *Cr. macrospora*, and one new species each of *Crinipellis* and *Moniliophthora* was described. Within *Marasmiaceae*, the subfamily names *Marasmieae* and *Crinipellieae* are corrected to *Marasmioideae* and *Crinipelloideae*, respectively. *Cr. xiaoxinganlingensis* nom. prov., with an uncertain systematic position, was also incorporated into *Crinipellis*. Accordingly, this genus is of great significance in resolving the taxonomic framework of *Crinipelloideae*. By compiling and discussing habitat information for the relevant species, this study further emphasized the value of ecological evidence for clarifying taxonomic boundaries and evolutionary relationships among genera. Moreover, by synthesizing diagnostic characters, clearer and more practical identification keys were constructed at multiple taxonomic levels, including the generic level within *Marasmiaceae* and the subgeneric, sectional, subsectional, and series levels within *Marasmius*. These findings have also substantially enriched the species diversity of *Marasmiaceae* in the Northeast China Ecological Region.

## Introduction

Members of *Marasmiaceae* Roze ex Kühner are mainly saprotrophic, functioning as key decomposers in the forest floor and litter layer (Pan et al. 2011; Læssøe and Petersen 2019; Zhang et al. 2024), whereas a small number of species are parasitic and can cause plant diseases. For example, *Paramarasmius mesosporus* (Singer) Antonín, K. Hosaka & Kolařík (= *Marasmiellus mesosporus* Singer) can lead to the large-scale death of grasses (Takehashi et al. 2007; Antonín et al. 2022). Additionally, rhizomorphs produced by some members of the family represent an active resource acquisition strategy that not only enables the retention and capture of leaf litter through a mycelial network but also potentially forms an indirect mutualistic relationship with nesting birds (Elliott et al. 2019). Moreover, species such as *Marasmius oreades* (Bolton) Fr. tend to form characteristic “fairy rings” (Hiltunen et al. 2019).

The taxonomic history of *Marasmiaceae* can be traced back to the studies by Fries, who established and proposed subgeneric, sectional, subsectional, and series ranks within *Marasmius* s.l. (Fries 1836). By examining European specimens, Kühner was the first to integrate microscopic structural characteristics into taxonomic criteria, particularly the amyloid reaction of stipe hyphae, thereby formally establishing the family *Marasmiaceae* Roze ex Kühner with *Marasmius* as its type genus (Kühner 1936, 1980). Singer validated and refined Kühner’s classification system based on specimens collected from a broader geographical range (Singer 1958, 1965). Subsequent infrageneric classification systems of *Marasmius* were mostly revisions and supplements to this established framework (Clémençon 1982; Desjardin and Petersen 1989; Antonín and Noordeloos 1993). With the application of molecular techniques, later studies based on expanded sampling confirmed three stable clades, namely *Omphalotaceae*, *Marasmiaceae*, and *Physalacriaceae*, which were subsequently recognized as independent families within *Agaricales* (Moncalvo et al. 2000, 2002; Wilson and Desjardin 2005; Matheny et al. 2006). Oliveira et al. (2024a) reassessed the phylogenetic boundaries of *Marasmiaceae* within *Marasmiineae* to establish a more stable family-level circumscription. This circumscription is largely consistent with the revised definition of taxa previously assigned to the subtribes *Marasmiinae* and *Crinipellinae* within *Tricholomataceae sensu* Singer (Singer 1986). Oliveira et al. (2024a) recognized two subfamilies within *Marasmiaceae*: *Marasmieae* and *Crinipellieae*. Subfam. *Marasmieae* is monotypic and contains only the genus *Marasmius*, whereas subfam. *Crinipellieae* comprises the genera *Chaetocalathus* Singer, *Crinipellis* Pat., *Moniliophthora* H.C. Evans, Stalpers, Samson & Benny, and *Paramarasmius* Antonín & M. Kolařík. The circumscription of subfam. *Crinipellieae* largely corresponds to subtrib. *Crinipellinae* as proposed by Singer (1976, 1986) but excludes *Lachnella* Fr. 1836, *Flagelloscypha* Donk, and *Amyloflagellula* Singer, which were incorporated into Singer’s original circumscription.

For *Crinipellis* [typified by *Crinipellis scabella* (Alb. & Schwein.) Murrill], the characters used for species delimitation include pileus coloration, basidium and basidiospore dimensions, cystidial morphology, the presence or absence of rhizomorphs, and the KOH reactions of pileus and stipe hairs or scales (Patouillard 1889). Most species of *Crinipellis* are saprotrophic, whereas a few are parasitic (Liu et al. 2018). Singer retained stipitate species within *Crinipellis* and transferred astipitate taxa to the allied *Chaetocalathus* (Singer 1942). However, during the early development of molecular systematics, the limited and highly fragmented sequence data available for *Crinipellis* obscured its phylogenetic relationships with allied genera. Previous phylogenetic studies recovered *Crinipellis* as sister to *Marasmius* s.s. (Moncalvo et al. 2002; Matheny et al. 2006; Bodensteiner et al. 2004), whereas another study resolved it as sister to a broader clade comprising *Chaetocalathus*, *Moniliophthora*, and *Marasmius* s.s. (Aime and Phillips-Mora 2005). *Moniliophthora* [typified by *Moniliophthora roreri* (Cif.) H.C. Evans, Stalpers, Samson & Benny] is characterized by long dextrinoid hairs similar to those of *Crinipellis*, but differs in having softer stipes and frequently producing pale red, purple, or orange pigments (Evans et al. 1978). *Paramarasmius* [with *Paramarasmius palmivorus* (Sharples) Antonín & Kolařík as the type species] differs from *Marasmius* by lacking a hymeniderm pileipellis, and from *Crinipellis*, *Moniliophthora*, and *Chaetocalathus* by lacking dextrinoid pileus hairs (Antonín et al. 2022).

Index Fungorum records 2090 *Marasmius* names, with more than 700 accepted species (accessed 10 June 2026, https://www.indexfungorum.org/). Studies on the species diversity of *Marasmiaceae* in China date back to the 1960s. Deng (1963) published the first records of four *Marasmius* species in “the monograph Fungi of China.” To date, approximately 220 *Marasmius* species have been reported from China. Roughly 110 *Crinipellis* species have been described worldwide, of which approximately 25 species have been documented in China, including *Cr. floccosa* T.H. Li, Y.W. Xia & W.Q. Deng, *Cr. bidens* T. Bau, and *Cr. setipes* (Peck) Singer (Xia et al. 2015; Liu et al. 2018; Wang et al. 2023). Thus far, three *Moniliophthora* species are known to occur in China. Among these, *Mo. sclerotium* L.P. Tang, W.Hao Zhang bis & G.Li Zhang were reported from Yunnan by Zhang et al. (2025). For *Chaetocalathus*, three species have been recorded, primarily from tropical regions of China: *Ch. liliputianus* (Mont.) Singer, *Ch. craterellus* (Durieu & Lév.) Singer, and *Ch. galeatus* (Berk. & M.A. Curtis) Singer (Wu et al. 2011; Li et al. 2015). These studies have been concentrated mainly in southern and southwestern China, particularly in Guangdong, Hainan, Yunnan, Guizhou, and Jiangxi provinces (Li et al. 1994; Zhu et al. 2022; Zhang 2023). Northeast China constitutes a key ecological region of China, encompassing diverse landscapes, including the Greater and Lesser Khingan Mountains, the Changbai Mountains, the Sanjiang Plain, the Liaohe River basin, and the Songnen Plain (Ministry of Natural Resources 2023). These complex ecosystems, which comprise cold-temperate coniferous forests, mixed broadleaved-coniferous forests, and extensive wetlands, provide a broad range of ecological niches for fungal growth and diversification. Approximately 66 marasmioid fungal species have been recorded from this region (Bi et al. 1985; Song et al. 2009; Deng 2012; Li et al. 2015; Chen and Zhang 2019; Bau 2024).

In this study, the specimens obtained over the past 4 years were identified through morphological characteristics and phylogenetic analyses based on ITS, nrLSU, *rpb*2, and *tef*1-α sequence data. Most of the examined specimens were collected from the Northeast China Ecological Region, and additional materials from other regions were also included. Among these, the specimens from Mongolia were of particular ecological interest, as they occupied a distinctive niche. The specific aims of this study were to (1) refine the delimitation of *Marasmius* and its allied genera by combining morphology, multilocus phylogeny, geographic distribution, and habitat information; and (2) improve the understanding of the species diversity of *Marasmiaceae* in the Northeast China Ecological Region.

## Materials and methods

### Specimen collection

The examined materials were collected from China and Mongolia, with a substantial proportion obtained from the Northeast China Ecological Region. During the survey (2022–2026), detailed records were made of the macroscopic characteristics of each specimen, including texture, color, odor, and moisture condition (whether dry or wet). Particular attention was paid to features such as the shape of the pileus, the presence or absence of striations, the development and attachment of the lamellae, and the presence of scales on the stipe. In addition, ecological characteristics were recorded, including whether the species was saprotrophic or parasitic, as well as the type of habitat or host on which it occurred. Photographs of each collection and its habitat were taken from multiple angles, and all specimens were assigned unique identification numbers. The geographical location of each specimen was recorded in detail. The specimens were deposited at the Fungarium of Jilin Agricultural University (FJAU). Specimen processing and morphological observations followed previously described methods (Aime et al. 2021; Zhu and Bau 2024).

### Morphology

Macromorphological features were recorded from fresh specimens in the field through photography, notes, and detailed descriptions. Basidiomata colors were determined according to the RAL CLASSIC color system established by the German Institute for Quality Assurance and Certification (Reichs-Ausschuss für Lieferbedingungen und Gütesicherung; https://www.ral-gütezeichen.de/).

Micromorphological examinations were performed with a light microscope. Thin hand-cut sections from both fresh basidiomata and dried materials were prepared under a stereomicroscope and mounted in distilled water, 5% KOH, Congo red, or Melzer’s reagent. Particular attention was given to the observation and imaging of basidiospores, basidia, cheilocystidia, pleurocystidia, pileipellis hyphae, pileus trama, stipitipellis hyphae, and stipe trama.

Basidiospores were examined and photographed using a Zeiss Merlin Optical Microscope with a high-resolution 1000× oil-immersion objective. For each specimen, no fewer than 30 mature basidiospores were measured, excluding the hilar appendix. Spore dimensions are reported as (*a*) *b–c* (*d*) × (*e*) *f–g* (*h*) in lateral view, where *a* and *d* indicate the minimum and maximum lengths, *e* and *h* indicate the minimum and maximum widths, and *b–c* and *f–g* represent the 90% confidence intervals. Measurements of spore size and *Q* values (length/width) are expressed in the format (*i*) *j–k* (*l*) [*m* ± *n*], where *i* and *l* indicate the minimum and maximum values, *j–k* represents the 90% confidence interval, and *m* ± *n* denotes the mean ± standard deviation.

Other microscopic structures, including basidia, cystidia, and hyphae, were observed and measured at 200× or 400× magnification. Basidial length was measured without sterigmata, while width was measured at the broadest part. The terminology and order used in the morphological descriptions followed the standardized format for *Marasmius* proposed by Oliveira et al. (2024a).

### DNA extraction, polymerase chain reaction, and sequencing

DNA extraction was performed using a modified CTAB method (Doyle 1987). DNA quality and PCR products were both examined using 1% agarose gel electrophoresis (Larsson and Örstadius 2008). The ITS region was amplified and sequenced using the primers ITS1F (5'-CTTGGTCATTTAGAGGAAGTAA-3') and ITS4 (5'-TCCTCCGCTTATTGATATGC-3') (Gardes and Bruns 1993; White et al. 1990). The nrLSU region was amplified and sequenced using the primers LR0R (5'-ACCCGCTGAACTTAAGC-3') and LR5 (5'-TCCTGAGGGAAACTTCG-3') (Vilgalys and Hester 1990). The *rpb*2 region was amplified and sequenced using the primers rpb2-6F (5'-TGGGGKWTGGTYTGYCCTGC-3') and rpb2-7R (5'-CCCATWGCYTGCTTMCCCAT-3') (Wang et al. 2004). The *tef*1-α region was amplified and sequenced using the primers EF1-983F (5'-GCYCCYGGHCAYCGTGAYTTYAT-3') and EF1-1567R (5'-ACHGTRCCRATACCACCRATCTT-3') (Rehner and Buckley 2005).

The PCR reaction mixture contained 1 μL of each primer, 2 μL of DNA template, 12.5 μL of BioTaq Master Mix (Sangon Biotech, Shanghai, China), and ddH_2_O to a final volume of 25 μL. PCR amplification was performed with an initial denaturation at 94 °C for 5 min, followed by 35 cycles of denaturation at 94 °C for 50 s, annealing at 50 °C for nrLSU and *tef*1-α, 52 °C for ITS, or 55 °C for *rpb*2 for 50 s, and extension at 72 °C for 1 min, with a final extension at 72 °C for 10 min. Sequencing services were provided by Sangon Biotech (Shanghai) Co., Ltd.

Raw reads were processed in BioEdit v. 7.7.1.0 (https://thalljiscience.github.io/page2.html), and sequence assembly and manual correction were carried out with DNAMAN v. 10.0 (https://www.dnaman.net/) to generate reliable consensus sequences. All newly obtained sequences were deposited in GenBank (https://www.ncbi.nlm.nih.gov/).

For phylogenetic analyses, representative sequences of related taxa were retrieved from GenBank and combined with the sequences generated in this study (Table 1). Multiple sequence alignments were conducted using the online MAFFT v. 7 platform (https://mafft.cbrc.jp/alignment/server/) under the G-INS-i algorithm. Phylogenetic analyses were performed in PhyloSuite using IQ-TREE v. 3 for maximum likelihood (ML) inference and MrBayes v. 3.2.7a for Bayesian inference (BI) (Ronquist et al. 2012; Zhao et al. 2025). The best-fit substitution models (without partitioning) were independently selected via ModelFinder; the Bayesian information criterion (BIC) was applied for BI analysis, whereas the Akaike information criterion (AIC) was adopted for ML analysis (Kalyaanamoorthy et al. 2017). ML analyses were conducted under the selected best-fit models, with nodal support assessed using 1,000 ultrafast bootstrap (UFBoot) replicates for each of the three datasets. BI analyses consisted of two independent parallel runs, with 15 M generations for dataset 1, 12 M generations for dataset 2, and 7 M generations for dataset 3. The first 25% of sampled trees were discarded as burn-in.

**Table 1.** Taxa used in the phylogenetic analyses.

**Taxon**	**Country**	**Collection**	**GenBank no**.				**Citation**
			**ITS**	**nrLSU**	***rpb*2**	***tef*1α**	
*Brunneocorticium* sp.	Guyana	MCA5803	MG717360	MG717348	–	–	Koch et al. 2018
*Campanella aff. buettneri*	China	TFB4133ss3	OQ171234	–	–	–	Petersen and Hughes 2024
*Ca. alba*	USA	TFB12565	DQ449943	–	–	–	Petersen and Hughes 2024
*Ca. buettneri*	Sao Tome and Principe	DED 8276	MF075136	MF075138	–	–	Desjardin 2017
*Ca. candida*	Cook Islands	PDD 102184	OQ282823	–	–	–	Direct Submission
*Ca. keralensis*	India	AF 342	MW462889	–	–	–	Direct Submission
*Ca. pustulata*	Australia	AQ793972	JX444168	–	–	–	Direct Submission
*Ca. simulans*	India	AF 129	MW506836	–	–	–	Direct Submission
*Ca.* sp.	China	TFB3075	OQ171237	–	–	–	Petersen and Hughes 2024
*Ca. tristis*	New Zealand	PDD_88322	PP407517	–	–	–	Direct Submission
*Chaetocalathus cocciformis*	New Zealand	JAC12666	OQ282795	–	–	–	Direct Submission
*Ch. columellifer*	–	AF330	MW295463	–	–	–	Direct Submission
*Ch. fragilis*	–	JFK122	FJ167662	–	–	–	Kerekes and Desjardin 2009
*Ch. galeatus*	–	JFK67	FJ167663	–	–	–	Kerekes and Desjardin 2009
*Ch. liliputianus*	USA	iNat59282449	OP028485	–	–	–	Direct Submission
*Ch. magnus*	–	DED4763	FJ167666	–	–	–	Kerekes and Desjardin 2009
*Crinipellis actinophora*	–	JFK139	FJ167626	–	–	–	Kerekes and Desjardin 2009
*Cr. bidens*	China	S14	MH143792	–	–	–	Liu et al. 2018
***Cr. bidens***	**China**	**FJAU69427**	**PZ269329**	–	**PZ277493**	**PZ277496**	**This study**
*Cr. birhizomorpha*	–	BRNM 751593	KF380831	KF380835	–	–	Antonín et al. 2014c
*Cr. brunneipurpurea*	Borneo	JFK84	OR636626	–	–	–	Oliveira et al. 2024a
*Cr. brunnescens*	–	BO DED6791 Type	NR_119705	–	–	–	Schoch et al. 2012
*Cr. cupreostipes*	Thailand	JFK131 Holotype	FJ167642	–	–	–	Kerekes and Desjardin 2009
*Cr. cupreostipes*	Thailand	JFK31	FJ167641	–	–	–	Kerekes and Desjardin 2009
*Cr. cupreostipes*	Thailand	JFK55	FJ167640	–	–	–	Kerekes and Desjardin 2009
*Cr. dipterocarpi*	Malaysia	TYS111	OR636627	OR656946	–	–	Oliveira et al. 2024a
*Cr. dipterocarpi*	–	DED7570	FJ167656	–	–	–	Kerekes and Desjardin 2009
*Cr. furcata*	–	JFK103	FJ167657	–	–	–	Kerekes and Desjardin 2009
***Cr. fuscosquamosa***	**China**	**FJAU69430**	**PZ269332**	**–**	**–**	**–**	**This study**
***Cr. fuscosquamosa***	**China**	**FJAU69429**	**PZ269331**	**PZ269352**	–	**PZ277497**	**This study**
***Cr. fuscosquamosa***	**China**	**FJAU69428 Holotype**	**PZ269330**	**–**	**–**	**–**	**This study**
***Cr. macrospora***	**China**	**FJAU69423**	**PZ269324**	**–**	**–**	**–**	**This study**
***Cr. macrospora***	**China**	**FJAU69422**	**PZ269323**	**–**	**–**	**PZ277494**	**This study**
***Cr. macrospora***	**China**	**FJAU69421**	**PZ269322**	–	**PZ277492**	**PZ277495**	**This study**
*Cr. malesiana*	Malaysia	TYS346	OR636628	–	–	–	Oliveira et al. 2024a
*Cr. malesiana*	–	BO AR491 Type	NR_119706	–	–	–	Schoch et al. 2012
*Cr. mezzanensis*	Italy	BRNM 766629	KP347691	–	–	–	Antonín et al. 2015
*Cr. nigricaulis*	–	CBM-FB-24125	FJ766094	–	–	–	Antonín et al. 2009
Cr. nigricaulis var. macrospora	–	BRNM 712569	FJ573196	–	–	–	Antonín et al. 2014c
*Cr. nigrolamellata*	France	LIP 0201684	MT946363	NG_074471	–	–	Direct Submission
*Cr. odorata*	India	CAL 1240 Type	NR_154802	–	–	–	Crous et al. 2016
*Cr. pallidipilus*	–	BRNM 751595	KF380833	–	–	–	Antonín et al. 2014c
*Cr. piceae*	–	DED7758	FJ167633	–	–	–	Kerekes and Desjardin 2009
***Cr. piceae***	**China**	**FJAU69424**	**PZ269325**	**PZ269346**	**–**	**–**	**This study**
*Cr. rhizomaticola*	–	BRNM 712570	FJ573198	MK277896	–	–	Antonín et al. 2014c
***Cr. rhizomaticola***	**China**	**FJAU67066**	**PZ269328**	**PZ269347**	**–**	**–**	**This study**
*Cr. scabella*	France	CBS 342.50	MH856653	MH868166	–	–	Vu et al. 2019
*Cr. setipes*	–	Bandala 4085	JF930643	JF930644	–	–	Bandala et al. 2012
*Cr. siamensis*	Thailand	CMUB40060	PP992079	–	–	–	Direct Submission
***Cr.* sp**.	**China**	**FJAU69426**	**PZ269327**	**PZ269345**	**–**	**–**	**This study**
***Cr.* sp**.	**China**	**FJAU69425**	**PZ269326**	**–**	**–**	**–**	**This study**
*Cr.* sp.	USA	iNat 140606160	OR987151	–	–	–	Canan et al. 2025
*Cr.* sp.	USA	iNaturalist 128812294	OP749082	–	–	–	Direct Submission
*Cr. subtomentosa*	Italy	GC 00143	OQ845979	–	–	–	Direct Submission
*Cr. tabtim*	–	CMU JFK129 Type	NR_119708	–	–	–	Schoch et al. 2012
*Cr. tabtim*	China	Li160629-31	OQ538294	–	–	–	Direct Submission
*Cr. wandoensis*	–	BRNM 751594 Type	NR_172747	NG_075187	–	–	Crous et al. 2020
***Cr. xiaoxinganlingensis* nom. prov**.	**China**	**FJAU69447**	**PZ280799**	**PZ274776**	**PZ277503**	**PZ277502**	**This study**
***Cr. xiaoxinganlingensis* nom. prov**.	**China**	**FJAU69448**	**PZ280800**	**–**	**–**	**–**	**This study**
*Cr. zonata*	–	KGC 253	FJ588911	–	–	–	Antonín et al. 2014c
*Cr. zonata*	–	DAOM176761	FJ167659	–	–	–	Kerekes and Desjardin 2009
*Gymnopus fusipes*	United Kingdom	Fruiting body 17	KF897021	–	–	–	Direct Submission
*Lentinula boryana*	Colombia	548	OM400526	–	–	–	Direct Submission
*Marasmius albisubiculosus*	Principe	DED 8277	KX953752	–	–	–	Shay et al. 2017
*Ma. aff. pallescens*	Thailand	NW424	EU935500	–	–	–	Wannathes et al. 2009
*Ma. albogriseus*	USA	iNaturalist 147797590	OR827183	–	–	–	Direct Submission
*Ma. albogriseus*	USA	iNaturalist 154704022	OR881234	–	–	–	Direct Submission
*Ma. albopurpureus*	–	N.K.Zeng2253	MT822924	–	–	–	Zeng and Jiang 2020
*Ma. albulus*	Thailand	NW627	MZ145122	–	–	–	Bhunjun et al. 2022
*Ma. altoribeirensis*	Brazil	JO532	KP635204	KP635158	OR896487	PP026056	Oliveira et al. 2020a
*Ma. apatelius*	Principe	DED8295	KX953762	–	–	–	Shay et al. 2017
*Ma. araucariae*	Thailand	NW364	EU935511	–	–	–	Wannathes et al. 2009
*Ma. auranticapitatus*	Brazil	SP445584 Type	NR_184982	–	–	–	Oliveira et al. 2022
*Ma. auranticapitatus*	Brazil	JO276	ON502667	ON502739	ON553941	ON553952	Oliveira et al. 2022
*Ma. aurantioferrugineus*	Republic of Korea	HCCN GBDS 2861	FJ904963	FJ904945	–	–	Antonín et al. 2010a
*Ma. auratus*	Thailand	NW175 Type	EU935502	–	–	–	Wannathes et al. 2009
*Ma. bambusiniformis*	Thailand	NW329	EU935521	–	–	–	Wannathes et al. 2009
*Ma. bellus*	Brazil	JO299	KP635208	KP635161	OR896483	PP026086	Oliveira et al. 2020a
*Ma. benghalensis*	India	SOUMITRA 240	MF189044	–	–	–	Boonmee et al. 2021
*Ma. berambutanus*	Malaysia	TYS503	FJ431226	–	–	–	Tan et al. 2009
*Ma. brunneolorobustus*	Australia	BRI AQ1018016 Type	NR_185427	–	–	–	Tan et al. 2022
*Ma. bulliardii*	Denmark	BRNM 705006	JN540056	–	–	–	Antonín et al. 2012a
*Ma. campestris*	China	NKZeng1243	KJ126767	–	–	–	Liang et al. 2017
*Ma. capillaris*	USA	TENN61532	FJ596827	–	–	–	Hughes et al. 2009
*Ma. castanocephalus*	Brazil	JO523	ON502679	–	ON553943	ON553955	Oliveira et al. 2022
*Ma. centrocinnamomeus*	South Korea	SFC 2018070402 Type	NR198336	–	–	–	Liu et al. 2024
*Ma. cf. fulvoferrugineus*	–	RA701031	MK217465	–	–	–	Alanbagi et al. 2019
*Ma. cf. grandisetulosus*	São Tomé	DED 8225	KX953743	–	–	–	Shay et al. 2017
*Ma. cf. occultatiformis*	China	MHHNU 30835	MK388150	–	–	–	Direct Submission
*Ma. chrysoblepharioides*	Argentina	SI-15-24	MF683956	–	–	–	Niveiro et al. 2018
***Ma. clavocystidiatus***	**China**	**FJAU69401**	**PZ271074**	**PZ269301**	**PZ023897**	**PZ028841**	**This study**
***Ma. clavocystidiatus***	**China**	**FJAU69402 Holotype**	**PZ271075**	**–**	**–**	**–**	**This study**
*Ma. coarctatus*		KD 1718	MG554638	–	–	–	Wang et al. 2018
*Ma. coasiaticus*	Brazil	SP445502 Type	NR_184984	–	–	–	Oliveira et al. 2022
*Ma. cohaerens*	Russia	LE253819	KF774173	–	–	–	Kiyashko et al. 2014
*Ma. cohaerens*	Germany	TENN 067916	KF774178	–	–	–	Kiyashko et al. 2014
*Ma. cohaerens* var. *mandshuricus*	Russia	LE 295986 Type	KF774171	–	–	–	Kiyashko et al. 2014
**Ma. cohaerens var. mandshuricus**	**China**	**FJAU69387**	**PZ271060**	**PZ269293**	**–**	**PZ361163**	**This study**
*Ma. coklatus*	Thailand	TYS301	EU935543	–	–	–	Wannathes et al. 2009
*Ma. colorimarginatus*	Principe	DED 8309	KX953745	–	–	–	Shay et al. 2017
*Ma. conchiformis*	Brazil	SP417471	JX424042	–	–	–	Oliveira et al. 2020a
*Ma. conchiformis*	–	JO45	KF741996	KF741997	–	PP026096	Oliveira et al. 2020a
**Ma. confertus var. tenuicystidiatus**	**China**	**FJAU69349**	**PZ271022**	–	–	–	**This study**
*Ma. congregatus*	Brazil	JO468	KP635168	KP635123	OR896478	–	Oliveira et al. 2020a
*Ma. corneri*	Thailand	NW269	OR636638	OR656963	–	–	Oliveira et al. 2024a
***Ma. coronatospinosus***	**China**	**FJAU69397 Holotype**	**PZ271070**	**PZ269298**	**PZ023876**	**PZ028858**	**This study**
***Ma. coronatospinosus***	**China**	**FJAU69398**	**PZ271071**	–	–	–	**This study**
***Ma. coronatospinosus***	**China**	**FJAU69399**	**PZ271072**	**PZ269299**	**PZ023873**	**PZ028859**	**This study**
***Ma. coronatospinosus***	**China**	**FJAU69400**	**PZ271073**	**PZ269300**	**PZ023870**	**PZ028860**	**This study**
*Ma. corrugatus*	Brazil	JO456	KP635171	KP635126	–	PP026085	Oliveira et al. 2020a
*Ma. crinipes*	Republic of Korea	BRNM 714695	FJ917628	–	–	–	Antonín et al. 2012b
*Ma. crinipes*	Republic of Korea	BRNM 714694 Type	FJ917629	–	–	–	Antonín et al. 2012b
***Ma. crinipes***	**China**	**FJAU69355**	**PZ271028**	–	–	–	**This study**
***Ma. crinipes***	**China**	**FJAU69358**	**PZ271031**	**PZ269276**	**PZ023878**	–	**This study**
*Ma. crinipes*	–	BRNM 714694	NR_172217	FJ917611	–	–	Antonín et al. 2012b
*Ma. crinis-equi*	Thailand	NW182	EU935564	–	–	–	Wannathes et al. 2009
*Ma. curreyi*	USA	iNat 230151571	PV911564	–	–	–	Canan et al. 2025
***Ma. curreyi***	**China**	**FJAU69419**	**PZ271092**	**PZ269313**	–	–	**This study**
*Ma. cystidiatus*	India	CAL 1669	MH216191	–	–	–	Sharafudheen and Manimohan 2018
***Ma. cystidiosus***	**China**	**FJAU69354**	**PZ271027**	**PZ269275**	**PZ023865**	**PZ028862**	**This study**
***Ma. cystidiosus***	**China**	**FJAU69445**	**PZ280802**	**–**	**–**	**–**	**This study**
***Ma. daqinggouensis***	**China**	**FJAU69391 Holotype**	**PZ271064**	**PZ269295**	**QB219565**	**PZ028844**	**This study**
***Ma. daqinggouensis***	**China**	**FJAU69392**	**PZ271065**	–	–	–	**This study**
***Ma. daqinggouensis***	**China**	**FJAU69393**	**PZ271066**	–	–	–	**This study**
*Ma. delectans*	United States	MycoMap 7736	MK532846	–	–	–	Direct Submission
*Ma. elaeocephaliformis*	São Tomé	DED8213 Type	KX953758	–	–	–	Shay et al. 2017
*Ma. elegans*	Australia	JAC11381	PP354966	–	–	–	Guard et al. 2024
*Ma. extremiorientalis*	–	IN06	OM473957	–	–	–	Direct Submission
*Ma. falcatipes*	–	RA8026	MK217464	–	–	–	Direct Submission
*Ma. falcatipes*	United States	DED5104	OR636644	OR656972	–	–	Oliveira et al. 2024a
*Ma. ferrugineodiscus*	South Korea	SFC 2016071457 Type	NR198337	–	–	–	Direct Submission
***Ma. ferrugineodiscus***	**China**	**FJAU69376**	**PZ271049**	**PZ269288**	**PZ023866**	PZ028852	**This study**
***Ma. ferrugineodiscus***	**China**	**FJAU69377**	**PZ271050**	–	–	–	**This study**
*Ma. ferrugineus*	Brazil	JO438	ON502682	ON502749	OR896403	PP026072	Oliveira et al. 2022
*Ma. ferruginoides*	Madagascar	JES209	KX148983	–	–	–	Shay et al. 2017
*Ma. fissuratus*	China	DC-2013 Type	KC191812	–	–	–	Li et al. 2015
***Ma. fissuratus***	**China**	**FJAU69370**	**PZ271043**	**PZ269284**	**PZ023858**	**PZ028867**	**This study**
*Ma. floridanus*	United States	DED5164	OR636645	OR656973	–	–	Li et al. 2015
*Ma. foliaceps*	Brazil	JO1146 Type	PP118111	–	–	–	Oliveira et al. 2024b
*Ma. galbinus*	China	GDGM 27251 Type	HQ709445	–	–	–	Deng and Li 2011
*Ma. gracilis*	Brazil	SP445425 Type	NR_177490	–	–	–	Oliveira et al. 2014
*Ma. graminicola*	China	HKAS134546	PP806301	–	–	–	Direct Submission
*Ma. graminipes*	Thailand	NW078 Type	EU935479	–	–	–	Wannathes et al. 2009
*Ma. graminum*	Denmark	NN005953	JN943595	–	JQ031102	–	Schoch et al. 2012
*Ma. griseopileatus*	KG232	KG232	KC415765	–	–	–	Antonín et al. 2014b
*Ma. guyanensis*	Brazil	JO344	MN714048	–	–	–	Oliveira et al. 2020a
*Ma. haematocephalus*	Brazil	JO533 Epitype	ON502673	ON502729	–	–	Oliveira et al. 2022
*Ma. haematocephalus*	Madagascar	JES193	KX148985	–	–	–	Shay et al. 2017
*Ma. haematocephalus*	India	AKD 202/2014	MF189055	–	–	–	Dutta 2017
*Ma. hinnuleus*	Madagascar	JES217	KX148988	–	–	–	Shay et al. 2017
*Ma. hobbitorum*	Brazil	SP 445484 Type	NR_177121	–	–	–	Oliveira et al. 2014
*Ma. hobbitorum*	–	JO309	KP635180	KP635135	–	PP026105	Oliveira et al. 2020b
***Ma. horqinensis***	**China**	**FJAU69325**	**PZ270998**	**PZ269257**	**PZ023887**	**PZ028853**	**This study**
***Ma. horqinensis***	**China**	**FJAU69326 Holotype**	**PZ270999**	–	–	–	**This study**
*Ma. hymenogloeus*	Ecuador	RLC38	OQ211606	–	–	–	Vandegrift et al. 2023
*Ma. hypochroides*	Thailand	NW405	EU935545	–	–	–	Wannathes et al. 2009
*Ma. imitarius*	Thailand	NW297	EU935496	–	–	–	Wannathes et al. 2009
*Ma. indojasminodorus*	India	AKD 135/2015 Type	KY785172	–	–	–	Phookamsak et al. 2019
*Ma. inflatus*	France	PB305/RA	AY571027	AY570990	–	–	Bodensteiner et al. 2004
*Ma. insolitus*	Russia	LE 289497 Type	KF774162	–	–	–	Kiyashko et al. 2014
***Ma. insolitus***	**China**	**FJAU69345**	**PZ271018**	–	–	–	**This study**
***Ma. insolitus***	**China**	**FJAU69347**	**PZ271020**	**PZ269271**	**PZ023896**	**–**	**This study**
*Ma. inthanonensis*	Thailand	NW353 Type	EU935514	–	–	–	Wannathes et al. 2009
*Ma. jasminodorus*	Thailand	NW294	EU935513	–	–	–	Wannathes et al. 2009
***Ma. jilinensis***	**China**	**FJAU69327 Holotype**	**PZ271000**	–	–	–	This study
*Ma. jinfoshanensis*	China	HGASMF014344 Type	MT556448	MW237220	–	–	Boonmee et al. 2021
***Ma. jinfoshanensis***	**China**	**FJAU69409**	**PZ271082**	**PZ269306**	**PZ023881**	–	**This study**
***Ma. jinfoshanensis***	**China**	**FJAU69411**	**PZ271084**	**PZ269308**	**PZ023885**	–	**This study**
***Ma. jinfoshanensis***	**China**	**FJAU69412**	**PZ271085**	**PZ269309**	**PZ023884**	–	**This study**
*Ma. kabakada*	Australia	Bransgrove	PP175853	PP175757	–	–	Guard et al. 2024
*Ma. katangensis*	Madagascar	JES 227	KX148991	–	–	–	Shay et al. 2017
*Ma. koae*	USA	DED6395 Type	OR636654	OR656989	–	–	Oliveira et al. 2024a
*Ma. laranja*	São Tomé	DED8231	KX953748	–	–	–	Shay et al. 2017
*Ma. laticlavatus*	Thailand	NW412	EU643511	–	–	–	Wannathes et al. 2009
*Ma. lentus*	China	110116MFBPL0385	MW554345	–	–	–	Direct Submission
*Ma. leucorotalis*	Brazil	JO498	MN714039	OR656992	OR896418	PP026114	Oliveira et al. 2020a
*Ma. leucorotalis*	Brazil	JO448	MN714040	–	–	–	Oliveira et al. 2020a
*Ma. lidaensis*	China	HKAS 148679	PX353111	–	–	–	Direct Submission
***Ma. lidaensis***	**China**	**FJAU69381**	**PZ271054**	**PZ269291**	**PZ023860**	**PZ028843**	**This study**
*Ma. longibasidiatus*	Brazil	JO444	MN714050	–	–	–	Oliveira et al. 2020a
*Ma. luteolus*	Thailand	NW138	EU935506	–	–	–	Wannathes et al. 2009
*Ma. luteoolivaceus*	Brazil	SP445574 Type	NR_177122	–	–	–	Oliveira et al. 2020b
*Ma. macrocystidiosus*	Russia	LE295996	KF774136	KF896246	–	–	Kiyashko et al. 2014
***Ma. macrocystidiosus***	**China**	**FJAU69383**	**PZ271056**	**–**	**–**	**PZ028832**	**This study**
*Ma. magnus*	–	Magnago_AC_1001	KX228846	–	–	–	Magnago et al. 2016
***Ma. maoershanensis***	**China**	**FJAU69414**	**PZ271087**	**PZ269311**	**PZ023869**	–	**This study**
***Ma. maoershanensis***	**China**	**FJAU69415 Holotype**	**PZ271088**	–	–	–	**This study**
***Ma. maoershanensis***	**China**	**FJAU69416**	**PZ271089**	–	–	–	**This study**
*Ma. margallensis*	Pakistan	LAH 37597 Type	NR_185783	–	–	–	Razzaq and Khalid 2023
*Ma. maximus*	China	FJAU69380	PZ271053	–	–	–	**This study**
***Ma. maximus***	**China**	**FJAU69379**	**PZ271052**	**PZ269290**	**PZ023864**	**PZ028870**	**This study**
*Ma. midnapurensis*	India	CUH AMT002 Type	KY785179	–	–	–	Dutta 2017
*Ma. nigrodiscus*	United States	DED5163	OR636667	OR657005	–	–	Oliveira et al. 2024a
*Ma. nivicola*	Republic of Korea	BRNM 714572	FJ904970	–	–	–	Antonín et al. 2010a
***Ma. occultatiformis***	**China**	**FJAU69342**	**PZ271015**	**PZ269267**	**PZ023891**	**PZ028837**	**This study**
***Ma. occultatiformis***	**China**	**FJAU69343**	**PZ271016**	**PZ269268**	**PZ023892**	**PZ028836**	**This study**
***Ma. occultatiformis***	**China**	**FJAU69344**	**PZ271017**	**PZ269269**	**PZ023888**	**PZ028835**	**This study**
*Ma. occultatus*	Thailand	BRNM 714698	FJ917623	–	–	–	Wannathes et al. 2009
*Ma. occultatiformis*	Russia	VLA M-21.215	KF774159	–	–	–	Kiyashko et al. 2014
*Ma. ochroleucus*	Thailand	NW299	EU935503	–	–	–	Wannathes et al. 2009
*Ma. oreades*	Serbia	BUNS1200735	MK226504	–	–	–	Direct Submission
***Ma. oreades***	**China**	**FJAU69378**	**PZ271051**	**PZ269289**	**PZ023874**	**PZ028869**	**This study**
*Ma. orientalis*	Thailand	NW315	EU935541	–	–	–	Wannathes et al. 2009
*Ma. pallidibrunneus*	Brazil	Marina_Capelari_4706	KP635186	NG_088034	OR896484	PP026059	Oliveira et al. 2020a
*Ma. pallidoaurantiacus*	Thailand	BKF10248 Type	MZ452673	–	–	–	Jayawardena et al. 2022
Ma. pallidocinctus var. latisporus	Brazil	JO164	MN714053	–	–	–	Oliveira et al. 2020a
*Ma. papyracea*	Colombia	NVE 228	KF937332	–	–	–	Vasco-Palacios et al. 2014
*Ma. pellucidus*	Thailand	NW321	EU935508	–	–	–	Wannathes et al. 2009
*Ma. pseudopellucidus*	Thailand	NW305 Type	EU935505	–	–	–	Wannathes et al. 2009
*Ma. puerariae*	Taiwan	R. Kirschner & C.-J. Chen 2139	JX470333	–	–	–	Kirschner et al. 2013
*Ma. pulcherripes*	Republic of Korea	BRNM 714692	FJ917615	–	–	–	Antonín et al. 2012b
*Ma. purpureostriatus*	Thailand	NW158	EU935539	–	–	–	Wannathes et al. 2009
*Ma. recreigardneri*	Brazil	JO491 Type	ON502683	ON502750	OR896404	PP026073	Oliveira et al. 2022
*Ma. roseobrunneus*	China	FH030	OQ450410	–	–	–	Direct Submission
*Ma. roseus*	Brazil	SP445510 Type	NR_184983	–	–	–	Oliveira et al. 2022
*Ma. rotalis*	Republic of Korea	BRNM 724479	JN003837	JN003843	–	–	Direct Submission
***Ma. rotalis***	**China**	**FJAU69404**	**PZ271077**	**PZ269303**	**PZ023882**	**PZ028842**	**This study**
***Ma. rotalis***	**China**	**FJAU69405**	**PZ271078**	–	–	–	**This study**
*Ma. rotula*	Canada	AW274	OR982383	OR657020	OR896415	–	Direct Submission
*Ma. rotula*	–	F26776	MZ310446	–	–	–	Direct Submission
*Ma. rotula*	USA	iNaturalist 236311577	PX306964	–	–	–	Direct Submission
*Ma. ruber*	Peru	FEB-351	ON426461	–	–	–	Direct Submission
*Ma. rubicundus*	Brazil	SP445549 Type	NR184981	–	–	–	Oliveira et al. 2022
*Ma. siccus*	Republic of Korea	BRNM 714680	FJ904985	–	–	–	Antonín et al. 2012b
*Ma. siccus*	United States	BRNM 552709	HQ607384	–	–	–	Sharafudheen and Manimohan 2018
*Ma. siccus*	United States	SFSUF029078	OR636675	OR657027	–	–	Oliveira et al. 2024a
***Ma. siccus***	**China**	**FJAU69388**	**PZ271061**	–	–	–	**This study**
***Ma. siccus***	**China**	**FJAU69389**	**PZ271062**	–	–	–	**This study**
***Ma. siccus***	**China**	**FJAU69390**	**PZ271063**	**PZ269294**	**–**	**PZ028848**	**This study**
*Ma. silvicola*	Brazil	JO366	KP635196	KP635150	OR896486	PP026061	Oliveira et al. 2024a
*Ma.* sp.	Thailand	NW232	EU935559	–	–	–	Wannathes et al. 2009
*Ma.* sp.	Japan	NOFFB6_F13	LC757621	–	–	–	Direct Submission
*Ma.* sp.	Japan	NOFFB7_F14	LC757623	–	–	–	Direct Submission
***Ma.* sp**.	**China**	**FJAU69337**	**PZ271010**	**PZ269263**	**PZ023893**	**PZ028833**	**This study**
***Ma.* sp**.	**China**	**FJAU69338**	**PZ271011**	–	–	–	**This study**
***Ma.* sp**.	**China**	**FJAU69403**	**PZ271076**	**PZ269302**	**PZ023895**	**–**	**This study**
*Ma.* sp.	China	110114MFBPL0022	MW554335	–	–	–	Direct Submission
***Ma.* sp**.	**China**	**FJAU69364**	**PZ271037**	**PZ269280**	**PZ023872**	**PZ028850**	**This study**
***Ma.* sp**.	**China**	**FJAU69365**	**PZ271038**	**–**	**–**	**–**	**This study**
*Ma.* sp.		T755	KC176355	–	–	–	Thorn et al. 1996
*Ma.* sp.		TMS2021647	OK586721	–	–	–	Direct Submission
*Ma.* sp.	–	BRNM 714697	FJ917619	–	–	–	Antonín et al. 2012b
*Ma.* sp.	–	BRNM 714700	FJ917620	–	–	–	Antonín et al. 2012b
*Ma.* sp.	–	58A_litter_C03	GU328594	–	–	–	Edwards and Zak 2010
*Ma.* sp.	–	B6	JX434662	–	–	–	Direct Submission
*Ma.* sp.	–	BUNS1200732	MK226496	–	–	–	Direct Submission
*Ma.* sp.	–	HBAU15684	MW871617	–	–	–	Direct Submission
*Ma.* sp.	–	TMS2021-6-62	OK586716	–	–	–	Direct Submission
*Ma.* sp.	–	SL1236	OQ147045	–	–	–	Direct Submission
*Ma.* sp.	–	YL220901	OQ147094	–	–	–	Direct Submission
*Ma.* sp.	–	JAC15803	OQ282814	–	–	–	Direct Submission
*Ma.* sp.	–	LAH35339	OQ998339	–	–	–	Direct Submission
*Ma.* sp.	–	iNaturalist 179090849	OR982383	–	–	–	Direct Submission
*Ma.* sp.	Cameroon	RAK 381	MN930630	–	–	–	Koch et al. 2018
*Ma.* sp.	China	FCATAS3406	PP622169	–	–	–	Direct Submission
***Ma.* sp**.	**China**	**FJAU69340**	**PZ271013**	**PZ269265**	**PZ023898**	**PZ028834**	**This study**
***Ma.* sp**.	**China**	**FJAU69406**	**PZ271079**	**PZ269304**	**PZ023880**	**PZ028840**	**This study**
*Ma.* sp.	Denmark	NN005958	JN943598	–	–	–	Schoch et al. 2012
*Ma.* sp.	Japan	07070	LC504806	–	–	–	Osono et al. 2021
*Ma.* sp.	Lithuania	clone_3993_1276	MW215412	–	–	–	Marčiulynienė et al. 2021
*Ma.* sp.	Madagascar	JES 150B	KX149001	–	–	–	Shay et al. 2017
*Ma.* sp.	Thailand	NW930	MN490094	–	–	–	Direct Submission
*Ma.* sp.	United States	TFB12243	HQ665550	–	–	–	Direct Submission
*Ma.* sp.	United States	DED5184	OR636646	OR656974	–	–	Oliveira et al. 2024a
***Ma.* sp**.	**China**	**FJAU69330**	**PZ271003**	**PZ269259**	**–**	**–**	This study
***Ma.* sp**.	**China**	**FJAU69352**	**PZ271025**	**–**	**PZ023863**	**–**	**This study**
***Ma.* sp**.	**China**	**FJAU69353**	**PZ271026**	**PZ269274**	**PZ023862**	**–**	**This study**
*Ma. spegazzinii*	Brazil	JO467	KP635197	–	OR896488	PP026075	Oliveira et al. 2022
*Ma. strobiluriformis*	Republic of Korea	BRNM 714915	GU266264	–	–	–	Antonín et al. 2010a
*Ma. subpurpureostriatus*	China	HFJAU4740	PV363556	–	–	–	Chen et al. 2025
***Ma. subpurpureostriatus***	**China**	**FJAU69420**	**PZ271093**	**PZ269314**	**PZ023871**	**PZ028851**	**This study**
*Ma. sullivantii*	United States	MycoMap 6672	MK564568	–	–	–	Direct Submission
*Ma. tageticolor*	Dominican Republic (DOM)	ANGE714	MT260147	–	–	–	Niveiro et al. 2022
*Ma. tenuilamellatus*	Russia	LE 295978	KF912952	–	–	–	Kiyashko et al. 2014
*Ma. torquescens*	Switzerland	Montri-228	MK028458	–	–	–	Hofstetter et al. 2019
*Ma. trinitatis*	Brazil	JO306	KP635200	KP635154	OR896472	–	Oliveira et al. 2020a
*Ma. tubulatus*	Thailand	NW239 Type	EU935560	–	–	–	Ramírez et al. 2021
*Ma. tucumanus*	Dominican Republic (DOM)	ANGE503	MT260145	–	–	–	Niveiro et al. 2022
***Ma. unicuspidatus***	**China**	**FJAU69384 Holotype**	**PZ271057**	**PZ269292**	–	–	**This study**
***Ma. unicuspidatus***	**China**	**FJAU69385**	**PZ271058**	–	–	–	**This study**
*Ma. vagus*	Australia	Ryan FLX-190425-F09	MT117840	–	–	–	Crous et al. 2019
*Ma. vallensis*	Republic of Korea	JS150904-16-1	KX963788	–	–	–	Lee et al. 2016
***Ma. variicystidiatus***	**China**	**FJAU69371**	**PZ271044**	**PZ269285**	**PZ023877**	**PZ028864**	**This study**
***Ma. variicystidiatus***	**China**	**FJAU69372**	**PZ271045**	**–**	**–**	**–**	**This study**
***Ma. variicystidiatus***	**China**	**FJAU69373 Holotype**	**PZ271046**	**PZ269286**	**PZ023857**	**PZ028863**	**This study**
***Ma. variicystidiatus***	**China**	**FJAU69374**	**PZ271047**	**–**	**–**	**–**	**This study**
***Ma. variicystidiatus***	**China**	**FJAU69375**	**PZ271048**	**PZ269287**	**PZ023879**	**PZ028865**	**This study**
*Ma. venatifolius*	Brazil	SP445485 Type	NR_177127	–	–	–	Oliveira et al. 2020a
*Ma. verrucosus*	Gabon	CBS 696.81	MH861439	MH873181	–	–	Vu et al. 2019
***Ma. vulgaris***	**China**	**FJAU69351**	**PZ271024**	**PZ269273**	**PZ023868**	**PZ028847**	**This study**
*Ma. wisteriae*	Republic of Korea	BRNM 718761 Type	JN003839	–	–	–	Antonín et al. 2012a
*Ma. wynneae*	United Kingdom	NFSG_20201020_01YM	OR999116	–	–	–	Direct Submission
*Ma. yunnanensis*	China	Dai 19857 Paratype	MW969679	–	–	–	Zhang et al. 2023
*Metacampanella coprophila*	Mongolia	FJAU69317	PP973102	PP973108	–	–	Hou et al. 2024
*Me. olivaceonigra*	Australia	AQ793973	JX444167	–	–	–	Direct Submission
*Me. tricolor*	China	FJAU69314	PP973103	PP973109	–	–	Hou et al. 2024
*Moniliophthora canescens*	–	FHMU3156	MW826222	–	–	–	Liu et al. 2021
*Mo. capitata*	Pakistan	LAH37907	OR387288	NG_243369	–	–	Haqnawaz et al. 2024.
*Mo. mayarum*	Pakistan	Lodge_BZ511	MT162718	–	–	–	Niveiro et al. 2020
*Mo. pakistanica*	Pakistan	LAH36969	MZ766451	OM033490	–	–	Izhar et al. 2022.
*Mo. purpurea*	Pakistan	LAH37902	OR387293	OR387123	–	–	Izhar et al. 2022
*Mo. sclerotium*	China	MHKMU L1-1	PV862919	–	–	–	Zhang et al. 2025
*Mo.* sp.	Mongolia	UBA23005	PQ732594	–	–	–	Direct Submission
***Mo.* sp**.	**China**	**FJAU69432**	**PZ269334**	–	–	–	**This study**
***Mo.* sp**.	**China**	**FJAU69431**	**PZ269333**	**PZ269348**	**PZ277489**	**PZ277498**	**This study**
***Mo.* sp**.	**China**	**FJAU69433**	**PZ269335**	**PZ269349**	**PZ277490**	**PZ277499**	**This study**
*Mo.* sp.	Argentina	CTES0568284	ON180675	–	–	–	Direct Submission
*Mo.* sp.	–	JZBHM006	KJ545432	–	–	–	Direct Submission
*Mo. ticoi*	–	NY00511157	MT162721	–	–	–	Niveiro et al. 2020
***Mo. vaginicola***	**Mongolia**	**FJAU69435**	**PZ269337**	**PZ269350**	**PZ277491**	**PZ277500**	**This study**
***Mo. vaginicola***	**China**	**FJAU69463 Holotype**	**QB209693**	**–**	**–**	**–**	**This study**
*Mycetinis alliaceus*	Italy	AMB 18771	ON263155	–	–	–	Consiglio et al. 2022
*Paramarasmius mesosporus*	–	AC13717	OQ282801	–	–	–	Direct Submission
***P. mesosporus***	**China**	**FJAU69436**	**PZ269338**	**PZ269351**	–	–	**This study**
*P. mesosporus*	New Zealand	JAC15622	PP407537	–	–	–	Direct Submission
*P. palmivorus*	–	DED6519-SFSU	–	AY639434	–	–	Wilson and Desjardin 2005
*Paragymnopus perforans*	France	CBS 209.47	MH856221	–	–	–	Vu et al. 2019
*Peckorumyces umbonatus*	China	HMJAU 60396	OP894912	OP889548	–	–	Li et al. 2024
*Pe. umbonatus*	–	PBM 2711 (CUW)	DQ486708	–	–	–	Matheny et al. 2006
*Tetrapyrgos atrocyanea*	Brazil	INPA259611	KT287095	–	–	–	Komura et al. 2020
*T. atrocyanea*	Brazil	INPA259597	KT287096	–	–	–	Komura et al. 2020
*T. brevicystidiata*	Brazil	INPA259604	KT287088	–	–	–	Komura et al. 2020
*T. cerebrata*	Brazil	INPA259594	KT287090	–	–	–	Komura et al. 2020
*T. crassicystidiata*	Brazil	INPA259606	KT287092	–	–	–	
*T. griseibrunnea*	Brazil	INPA259608	KT287097	–	–	–	Komura et al. 2020
*T. griseibrunnea*	Brazil	INPA259609	KT287099	–	–	–	Komura et al. 2020
*T. nigripes*	USA	TFB12137	DQ449941	–	–	–	Hughes et al. 2009
*T. nigripes*	USA	iNaturalist 128220417	OP743473	–	–	–	Direct Submission
***T. nigripes***	**China**	**FJAU69438**	**PZ269340**	**PZ269353**	–	**PZ277501**	**This study**
***T. nigripes***	**China**	**FJAU69437**	**PZ269339**	**PZ269354**	–	–	**This study**
***T. nigripes***	**China**	**FJAU69439**	**PZ269341**	–	–	–	**This study**
***T. nigripes***	**China**	**FJAU69440**	**PZ269342**	–	–	–	**This study**
*T. subcinerea*	–	AHH129	EF175526	–	–	–	Honan et al. 2015
*T. subcinerea*	–	AR505	EF175530	–	–	–	Honan et al. 2015

**Note**: Sequences generated in this study are indicated in bold. ‘-’ indicates that no sequence was obtained in this study.

The phylogenetic tree of *Marasmiaceae* was reconstructed with selected species of *Omphalotaceae* as outgroups. The phylogenetic tree of *Marasmius* was reconstructed using species of *Crinipellis* and *Paramarasmius* as outgroups. These selections of outgroups followed the study by Oliveira et al. (2024a).

Phylogenetic trees were visualized, and data were analyzed using ChiPlot (https://www.chiplot.online/gene_cluster.html).

### Abbreviations

For Latin names: *Ca.* = *Campanella*; *Ch.* = *Chaetocalathus*; *Co.* = *Collybiopsis*; *Cr.* = *Crinipellis*; *Ma.* = *Marasmius*; *Me.* = *Metacampanella*; *Mo.* = *Moniliophthora*; *P.* = *Paramarasmius*; *Pe.* = *Peckorumyces*; *T.* = *Tetrapyrgos*.

## Results

### Phylogeny

A total of 531 sequences were used in the phylogenetic analyses, comprising 332 ITS, 95 nrLSU, 54 *rpb*2, and 50 *tef*1-α sequences (sequences reused across different datasets were counted only once). Of these, 209 sequences were newly generated, including 84 ITS, 49 nrLSU, 40 *rpb*2, and 36 *tef*1-α sequences. Three multilocus datasets were assembled for phylogenetic analyses.

The first dataset, used to construct the phylogenetic tree of *Marasmius* subg. *Globulares*, included 296 sequences, comprising 173 ITS, 51 nrLSU, 37 *rpb*2, and 35 *tef*1-α sequences. The concatenated alignment contained 2,904 characters, including 991 bp for ITS, 907 bp for nrLSU, 450 bp for *rpb*2, and 556 bp for *tef*1-α. The ML and BI analyses were performed under the best-fit model GTR+I+G4+F. The ASDSF was 0.0095. The average effective sample size (avg ESS) for all parameters was 1,014.99.

The second dataset, used to construct the phylogenetic tree of *Marasmius* subg. *Marasmius* sect. *Marasmius*, included 79 sequences, comprising 53 ITS, 12 nrLSU, 10 *rpb*2, and four *tef*1-α sequences. The concatenated alignment contained 2,788 characters, including 903 bp for ITS, 891 bp for nrLSU, 450 bp for *rpb*2, and 544 bp for *tef*1-α. ML phylogenies were inferred under the best-fit model GTR+I+G4+F. BI phylogenies were inferred under the GTR+I+G+F model. The ASDSF was 0.0094. The average effective sample size (avg ESS) for all parameters was 765.1.

The third dataset, used to reconstruct the phylogenetic framework to infer relationships within *Marasmiaceae* and related lineages, included 165 sequences, comprising 114 ITS, 32 nrLSU, 7 *rpb*2, and 12 *tef*1-α sequences. The concatenated alignment contained 2,883 characters, including 966 bp for ITS, 915 bp for nrLSU, 447 bp for *rpb*2, and 555 bp for *tef*1-α. Phylogenetic frameworks were reconstructed using both ML and BI methods. ML analyses employed the GTR+I+G4+F substitution model. BI analyses were performed under the GTR+F+I+G4 model. The MCMC analysis converged successfully with an ASDSF value of 0.0080. The average effective sample size (avg ESS) for all parameters was 516.43.

ML and BI analyses generated largely congruent topologies. Therefore, the ML trees are presented, with ML bootstrap values and Bayesian posterior probabilities indicated at the nodes.

### Phylogenetic relationships within *Marasmiaceae*

**Figure 1.**
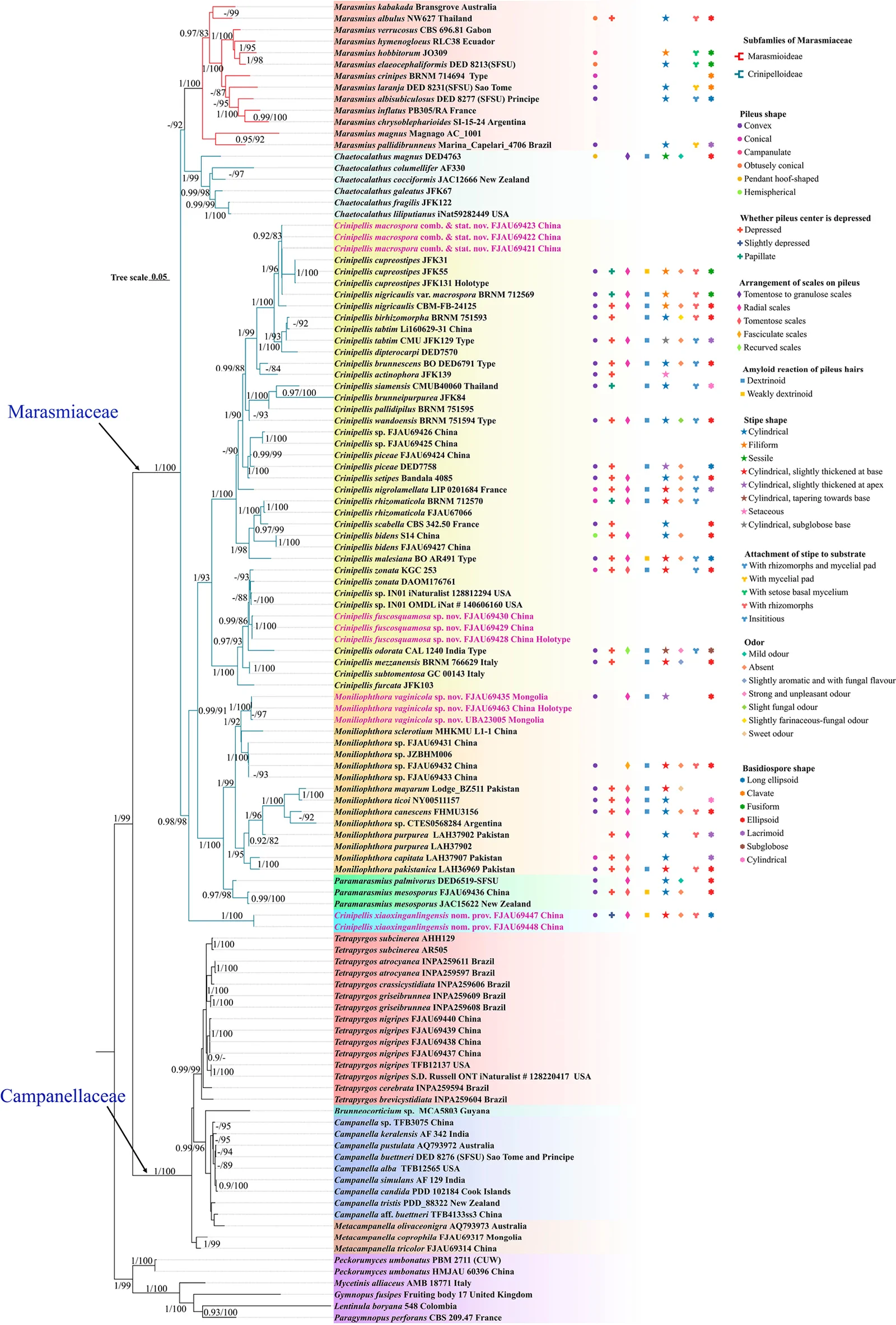
Maximum likelihood phylogeny of *Marasmiaceae* and related lineages inferred from a concatenated four-locus dataset (ITS, nrLSU, *rpb*2, and *tef*1-α). The topology shown is the ML tree. Bayesian posterior probabilities (PP) and maximum likelihood ultrafast bootstrap support values (UFBoot) are shown at the nodes in the order PP/UFBoot. Only PP values ≥ 0.90 and UFBoot values ≥ 70% are indicated. The scale bar represents 0.05 substitutions per site. Colored blocks indicate genus-level taxonomic units. Newly identified taxa are highlighted in pink type. A matrix summarizing selected morphological characters of representative *Marasmiaceae* taxa included in the phylogeny is shown to the right of the tree. The symbols and color coding used in the matrix are defined in the accompanying key.

*Campanellaceae* was recovered as a basal clade sister to *Marasmiaceae* with strong support (PP = 1.00, UFBoot = 99%). Within *Marasmiaceae*, two major clades were recovered (Fig. 1). The first major clade (UFBoot = 92%) accommodated the genera *Marasmius* and *Chaetocalathus*. The second major clade (PP = 0.98, UFBoot = 98%) contained two sister clades: the *Crinipellis*–*Moniliophthora*–*Paramarasmius* clade (PP = 0.99, UFBoot = 91%) and the *Cr. xiaoxinganlingensis* nom. prov. clade (PP = 1.00, UFBoot = 100%). Within the *Crinipellis*–*Moniliophthora*–*Paramarasmius* clade, the genus Crinipellis (PP = 1.00, UFBoot = 93%) was recovered as the sister group to a clade consisting of *Moniliophthora* (PP = 1.00, UFBoot = 99%) and *Paramarasmius* (PP = 0.97, UFBoot = 98%).

Taxa currently classified under *Crinipellis* were split into two independent clades. The first clade included most *Crinipellis* species, among which *Cr. fuscosquamosa* T. Bau & W.N. Hou, sp. nov. formed a well-supported monophyletic clade (PP = 1, UFBoot = 100%). *Cr. macrospora* (Antonín, Ryoo & H.D. Shin) T. Bau & W.N. Hou, stat. nov. and *Cr. cupreostipes* Kerekes, Desjardin & Lumyong (JFK131 holotype, JFK31, and JFK55) formed a well-supported clade (PP = 0.92, UFBoot = 83%), which was recovered as sister to the clade containing *Cr. nigricaulis* Har. Takah. (CBM-FB-24125). The second *Crinipellis* clade (PP = 1, UFBoot = 100%) was represented by sequences derived from the specimens FJAU69447 and FJAU69448, corresponding to *Cr. xiaoxinganlingensis* nom. prov. However, the phylogenetic affinity of this taxon with other *Crinipellis* members lacked robust statistical support, and its taxonomic position remains ambiguous and requires further verification. *Mo. vaginicola* T. Bau & W.N. Hou, sp. nov. (UBA23005, FJAU69435, and FJAU69463) formed a well-supported clade (PP = 1.00, UFBoot = 100%) and was recovered as sister to another clade (PP = 1, UFBoot = 92%) that comprised *Mo.* sp. (FJAU69431, FJAU69432, and FJAU69433), *Mo. sclerotium* (MHKMU L1-1), and an unidentified *Mo.* sp. (JZBHM006).

### Phylogenetic tree of *Marasmius*

**Figure 2.**
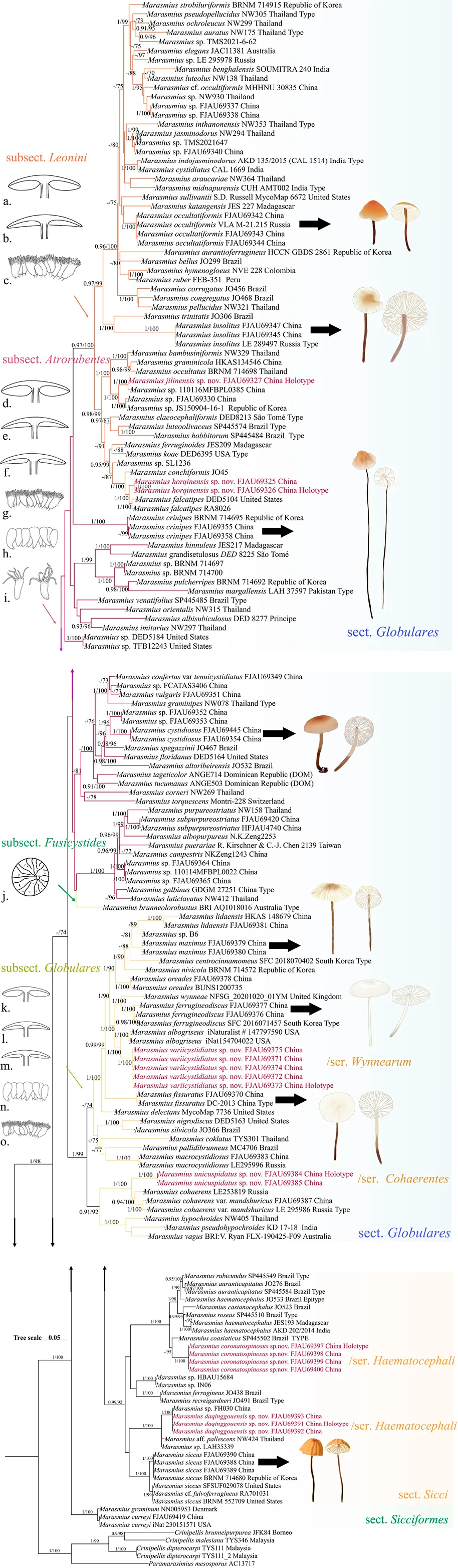
Maximum likelihood phylogeny of *Marasmius* subg. *Globulares* inferred from a concatenated four-locus dataset (ITS, nrLSU, *rpb*2, and *tef*1-α). The topology shown is the ML tree. Bayesian posterior probabilities (PP) and maximum likelihood ultrafast bootstrap support values (UFBoot) are indicated at the nodes in the order PP/UFBoot. Only PP values ≥ 0.90 and UFBoot values ≥ 70% are shown. The scale bar represents 0.05 substitutions per site. Newly identified taxa are highlighted in pink type. Colored branch blocks indicate section-level taxonomic units, and different branch colors indicate different subsection-level taxonomic units. Annotations on the left side of the tree summarize lamellar attachment, stipe insertion, and pileipellis cell types for each subsection within sect. *Globulares*. Partial illustrations of basidiomata of selected species, represented by specimens collected in this study, are shown on the right side of the tree. Subsect. *Leonini*: stipe centrally inserted; lamellae (a) adnexed and (b) adnate; pileipellis cells (c) *Siccus*-type. Subsect. *Atrorubentes*: stipe centrally inserted; lamellae (d) adnexed, (e) adnate, and (f) free; pileipellis cells predominantly (h) *Siccus*-type, less commonly (i) *Globulares*-type, and very rarely *Amyloflagellula*-type. Subsect. *Fusicystides*: (j) stipe eccentrically inserted and lamellae intervenose. Subsect. *Globulares*: stipe centrally inserted; lamellae (k) adnexed, (l) adnate, and (m) free; pileipellis cells predominantly (n) *Globulares*-type, with (o) *Siccus*-type cells occurring less frequently.

The phylogenetic tree was constructed for *Marasmius* subg. *Globulares* (Fig. 2), with *Marasmius* subg. *Marasmius* sect. *Sicciformes* resolved as the sister clade. Two sections were identified within *Marasmius* subg. *Globulares*: sect. *Sicci* (PP = 0.99, UFBoot = 92%) and sect. *Globulares* (UFBoot = 74%).

Sect. *Globulares* consists of four subsections: subsect. *Leonini*, subsect. *Atrorubentes*, subsect. *Globulares*, and subsect. *Fusicystides*. Among them, subsect. *Atrorubentes* was a monophyletic group, while the other three subsections each formed independent monophyletic clades. Within subsect. *Leonini*, *Ma. jilinensis* T. Bau & W.N. Hou, sp. nov. was recovered as sister to the unidentified *Marasmius* collection (110116MFBPL0385). These two taxa collectively formed a well-supported clade (PP = 1, UFBoot = 100%), which was sister to another fully supported clade (PP = 1, UFBoot = 100%) containing *Ma. bambusiniformis* Singer, *Ma. graminicola* Speg., and *Ma. occultatus* Har. Takah. *Ma. horqinensis* T. Bau & W.N. Hou, sp. nov. (FJAU69325, FJAU69326) also formed a well-supported clade and was recovered as the sister species of *Ma. falcatipes* Desjardin. Within subsect. *Globulares*, *Ma. variicystidiatus* T. Bau & W.N. Hou, sp. nov. and *Ma. unicuspidatus* T. Bau & W.N. Hou, sp. nov. were each placed in separate, well-supported clades. The former belongs to ser. *Wynnearum*, whereas the latter was nested within ser. *Cohaerentes*.

Within sect. Sicci, *Ma. coronatospinosus* T. Bau & W.N. Hou, sp. nov. and *Ma. daqinggouensis* T. Bau & W.N. Hou, sp. nov. each formed a distinct, well-supported monophyletic clade (PP = 1, UFBoot = 100%). These two species were assigned to ser. *Haematocephali*.

**Figure 3.**
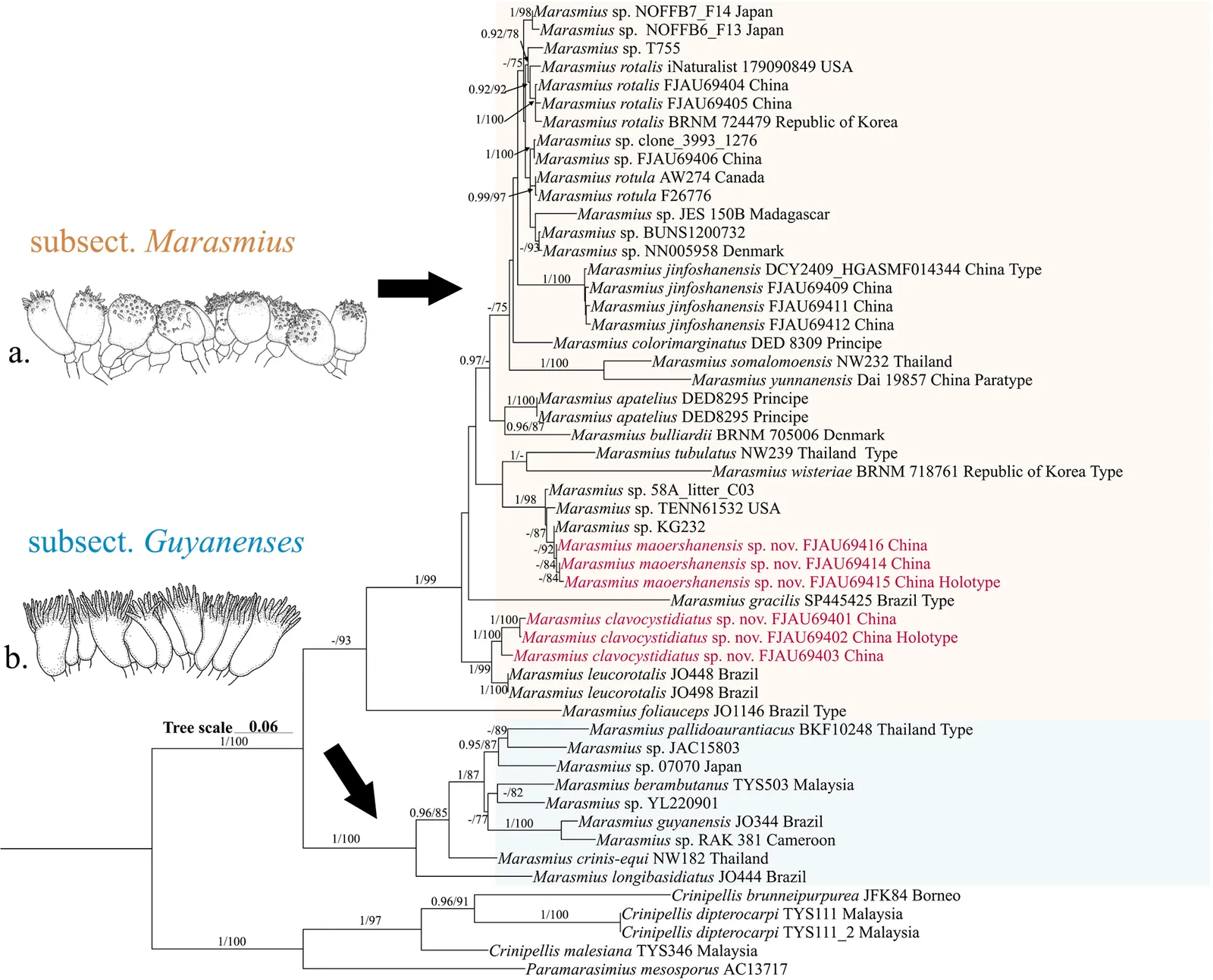
Maximum likelihood phylogeny of *Marasmius* subg. *Marasmius* sect. *Marasmius* inferred from a concatenated four-locus dataset (ITS, nrLSU, *rpb*2, and *tef*1-α). The topology shown is the ML tree. Bayesian posterior probabilities (PP) and maximum likelihood ultrafast bootstrap support values (UFBoot) are indicated at the nodes in the order PP/UFBoot. Only PP values ≥ 0.90 and UFBoot values ≥ 70% are shown. The scale bar represents 0.06 substitutions per site. Newly identified taxa are highlighted in pink type. Colored branch blocks indicate subsection-level taxonomic units. Subsection names are shown to the left of the tree, with text colors corresponding to the associated branch blocks. Illustrations of diagnostic morphological features characteristic of the two subsections are provided: (a) *Rotalis*-*type* pileipellis cells and (b) *Siccus*-type pileipellis cells.

The second dataset yielded a phylogenetic tree of *Marasmius* subg. *Marasmius* sect. *Marasmius* (Fig. 3). Two major clades were recovered within this section, corresponding to subsect. *Marasmius* (UFBoot = 93%) and subsect. *Guyanenses* (PP = 1, UFBoot = 100%). *Ma. maoershanensis* T. Bau & W.N. Hou, sp. nov. and *Ma. clavocystidiatus* T. Bau & W.N. Hou, sp. nov. were both nested within subsect. *Marasmius*. *Ma. maoershanensis* (FJAU69416, FJAU69414, and FJAU69415, holotype) and three unidentified *Marasmius* collections (58A_litter_C03, TENN61532, KG232) formed a well-supported clade (PP = 1, UFBoot = 98%). This clade was recovered as sister to the clade comprising *Ma. tubulatus* Petch and *Ma. wisteriae* Antonín, Ryoo & H.D. Shin. *Ma. clavocystidiatus* (FJAU69403, FJAU69401, and FJAU69402, holotype) formed a well-supported clade and was recovered as sister to *Ma. leucorotalis* Singer (JO448, JO498).

### Taxonomy

#### Marasmiaceae

**Revision of subfamilies of *Marasmiaceae***

**Notes**. Oliveira et al. (2024a) originally proposed the names *Marasmieae* and *Crinipellieae* at the subfamily rank. According to the International Code of Nomenclature for algae, fungi, and plants (ICN), however, subfamily names should bear the suffix -oideae, while the suffix -eae applies exclusively to tribal-rank names and is thus invalid for subfamily names (ICN, Art. 19.7) (Turland et al. 2018). Accordingly, these two names are corrected to *Marasmioideae* and *Crinipelloideae*, respectively. Author citations and publication dates remain unchanged in compliance with the Code (see ICN, Art. 32.2) (Turland et al. 2018). The circumscription and diagnostic definitions of these two revised subfamilies are identical to the original concepts proposed by Oliveira et al. (2024a).

***Crinipelloideae* J.S. Oliveira, Desjardin & Moncalvo, Fungal Diversity 127: 27 (2024) [MB 865521 ] originally published as ' *Crinipellieae* '; published with an improper Latin termination, corrected under Art. 19.7 (Madrid), see Art. 32.2**

**Type-genus**. *Crinipellis* Pat.

**Notes**. In the present analysis, the phylogenetic placements of *Crinipellis*, *Moniliophthora*, and *Paramarasmius* are clearly resolved, whereas the taxonomic position of *Chaetocalathus* remains ambiguous.

***Marasmioideae* J.S. Oliveira, Desjardin, Jenkinson, Margaritescu, Capelari & Moncalvo, Fungal Diversity 127: 27 (2024) [MB 865520 ] originally published as ' *Marasmieae* '; published with an improper Latin termination, corrected under Art. 19.7 (Madrid), see Art. 32.2**

**Type-genus**. *Marasmius* Fr.

**Notes**. The circumscription and definition of this revised subfamily retain the original taxonomic concept, and this subfamily remains monotypic.

##### Key to the genera of *Marasmiaceae*

**Table:** 

1	Basidiomata sessile or subsessile, laterally attached to the substrate	*** Chaetocalathus ***
–	Basidiomata stipitate, stipe central, eccentric, or lateral	**2**
2	Pileipellis of the hymeniderm type	*** Marasmius ***
–	Pileipellis of the cutis type	3
3	Pileipellis lacking setae	*** Paramarasmius ***
–	Pileipellis with dense setae	4
4	Pileus margin usually hairy	*** Crinipellis ***
–	Pileus margin usually smooth, glabrous	*** Moniliophthora ***

#### Marasmius

**Sections of *Marasmius* subg. *Globulares***

**Notes**. *Marasmius* subg. *Globulares* currently accommodates two sections, sect. *Globulares* and sect. *Sicci*. Sect. *Sicci* is morphologically close to sect. *Globulares*, and several diagnostic features of sect. *Sicci*, including marasmioid basidiomata and Siccus-type pileipellis cells, are also present in species traditionally assigned to sect. *Globulares*. Owing to the extensive morphological variation within sect. *Globulares*, its circumscription covers most of the morphological characteristics observed in sect. *Sicci*, rendering morphological delimitation between the two sections unreliable. As such, sect. *Sicci* is recognized as a distinct clade based on phylogenetic evidence, with its morphological delimitation defined by a suite of combined traits: small, thin basidiomata, filiform chitinous stipes, elongate basidiospores, and generally well-developed refractive pleurocystidia.

##### Key to subsections of *Marasmius* subg. *Globulares* sect. *Globulares*

**Table:** 

1	Pleurocystidia with resinous secretion	**subsect. *Fusicystides***
–	Pleurocystidia lacking resinous secretion	**2**
2	Caulocystidia absent in most species	**subsect. *Atrorubentes***
–	.Caulocystidia present in most species	**3**
3	Pileipellis mainly composed of *Siccus*-type cells	**subsect. *Leonini***
–	Pileipellis mainly composed of *Globulares*-type cells	**subsect. *Globulares***

##### Key to *Marasmius* subg. *Globulares* sect. *Globulares* subsect. *Leonini* (partial)

**Table:** 

1	Basidiospores with maximum length > 15 µm	**ser. *Graminicolae***
–	Basidiospores with maximum length ≤ 15 µm	**ser. *Leonini***

**Notes**. Four series-level taxa are currently recognized within subsect. *Leonini*. Nevertheless, morphological boundaries among these series remain poorly resolved. ser. *Leonini* and ser. *Luteoli* exhibit substantial overlap in diagnostic characters and cannot be reliably distinguished by morphology alone. Similarly, ser. *Graminicolae* and ser. *Bambusiniformis* show considerable overlap in their core morphological traits. Variations among these series appear to correlate with geographical distribution patterns, with ser. *Leonini* and ser. *Graminicolae* predominantly comprising tropical species.

###### Marasmius jilinensis

Taxon classificationFungiAgaricalesMarasmiaceae

T. Bau & W.N. Hou sp. nov.

55C1A17D-AD2C-5208-9CF9-7571C610BD97

863737

Figs 4A–C, 6

####### Diagnosis.

It is similar to *Ma. brunneoaurantiacus* Antonín & Buyck but differs in having a non-umbilicate pileus center, sinuate lamellae, and lacrymoid basidio­spores [*Q* = (2.58) 2.74–4.02 (4.29)].

**Figure 4.**
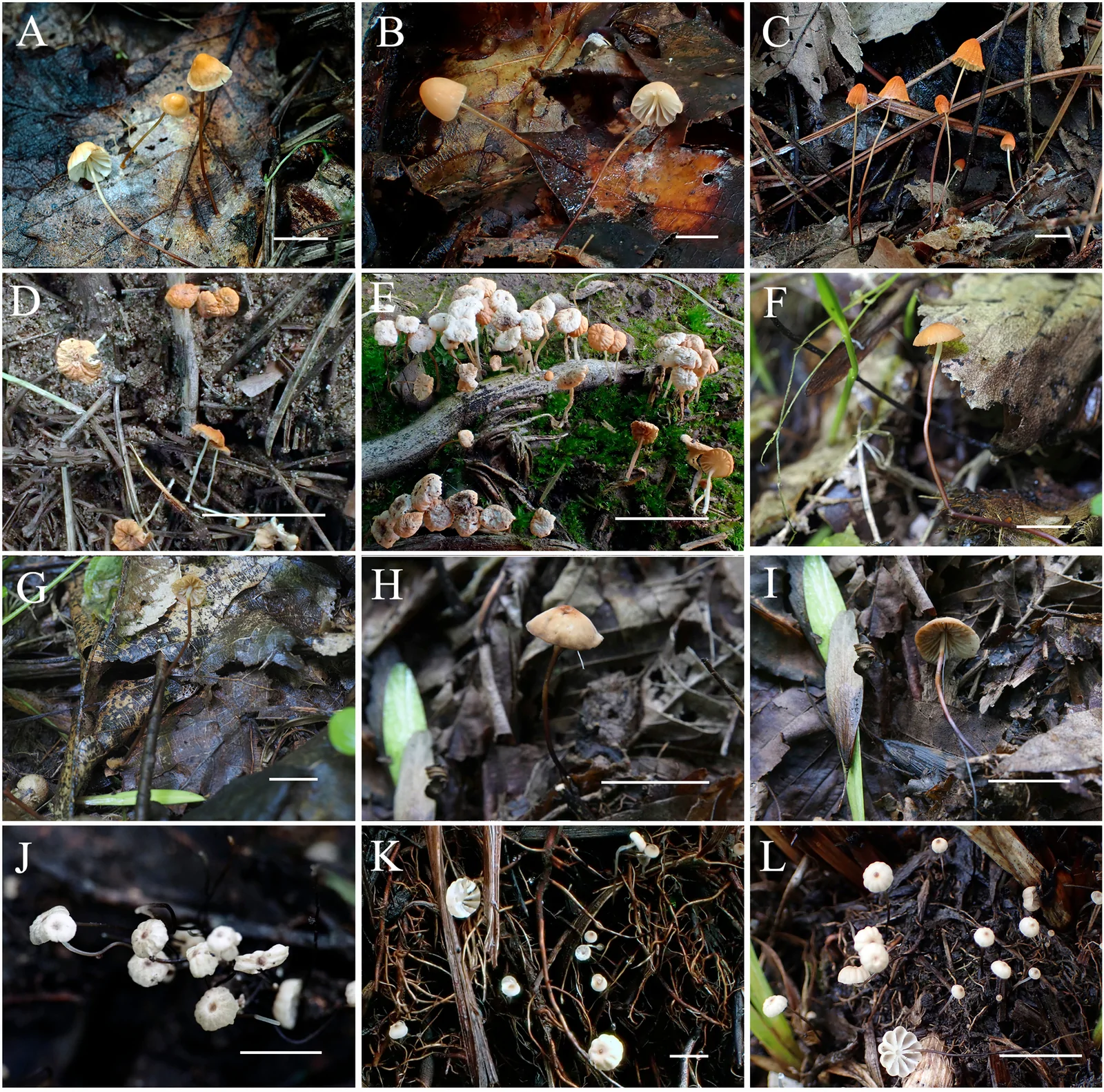
Basidiomata of *Marasmius* species (part 1). *Ma. jilinensis*, **A** FJAU69462 (H26070807); **B** FJAU69329 (Q247718); **C** FJAU69327 (E230797), holotype!. *Ma. horqinensis*, **D** FJAU69326 (gf241), holotype!; **E** FJAU69325 (C2481401). *Ma. unicuspidatus*, **F, G** FJAU69384 (H25070602), holotype!; **H, I** FJAU69385 (Y2572031). *Ma. clavocystidiatus*; **J** FJAU69455 (H26060013); **K** FJAU69401 (C2472801); **L** FJAU69402 (S24072803), holotype!. Scale bars: 1 cm (**A–L**).

**Figure 5.**
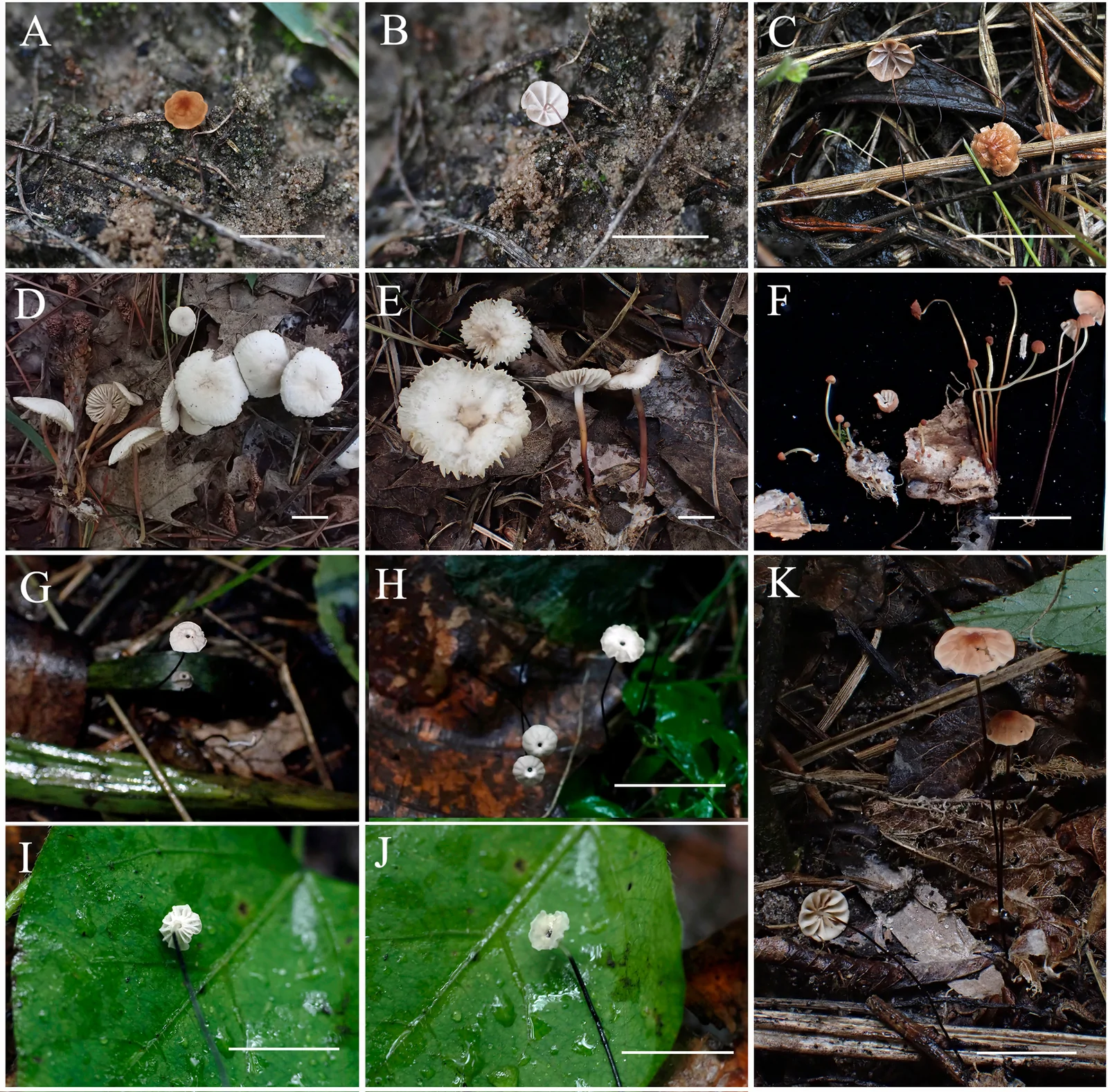
Basidiomata of *Marasmius* species (part 2). *Ma. coronatospinosus*, **A, B** FJAU69400 (H25080103). **C** FJAU69397 (H2472818), holotype!. *Ma. variicystidiatus*. **D** FJAU69371 (H220787). **E** FJAU69373 (m05), holotype!. *Ma. daqinggouensis*. **F** FJAU69391 (H2307121), holotype!. **K** FJAU69392 (Y2371637). *Ma. maoershanensis*. **G** FJAU69414 (H230790). **H** FJAU69457 (H26070802). **I, J** FJAU69415 (H25070510), holotype!. Scale bars: 1 cm (**A–K**).

**Figure 6.**
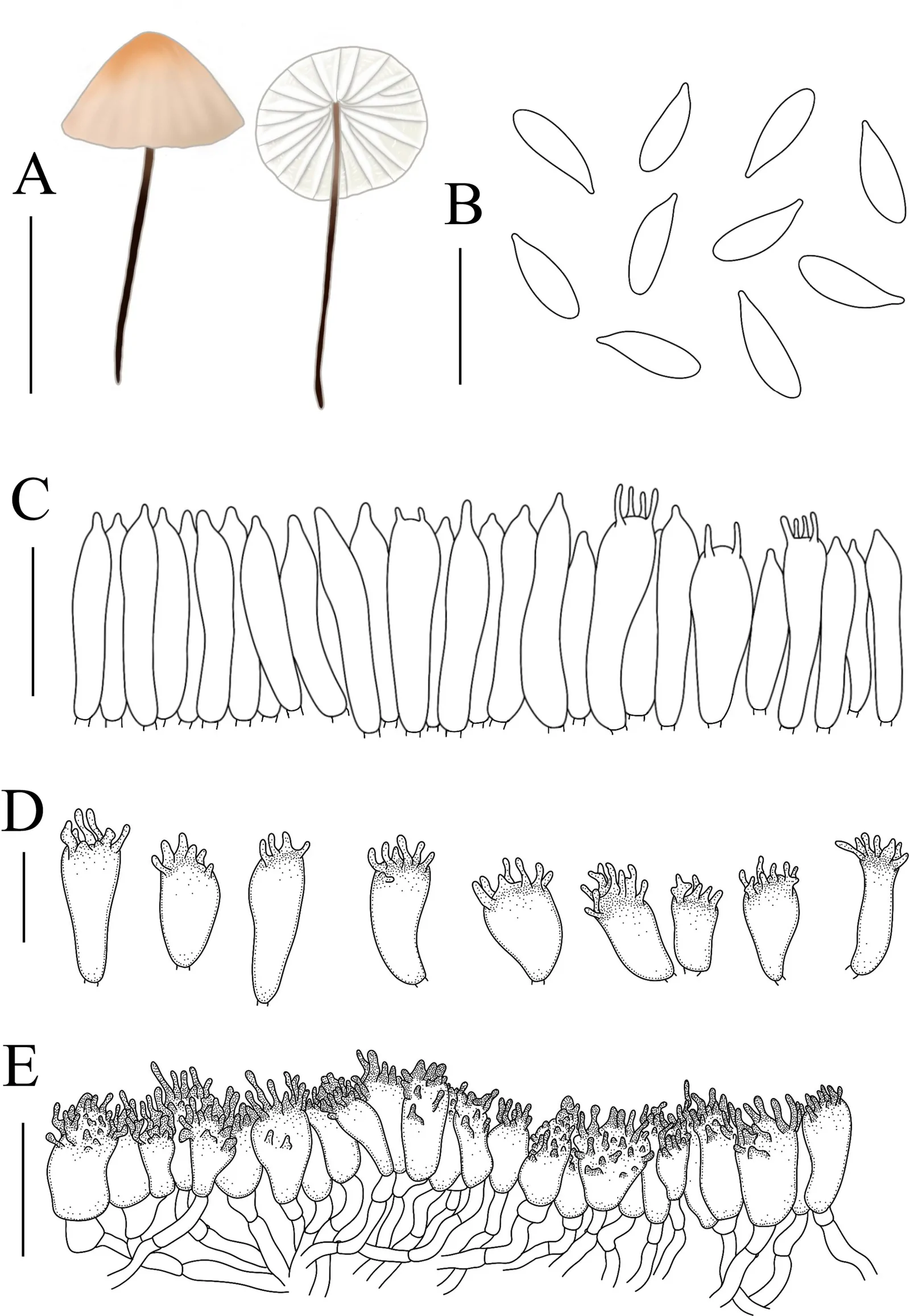
Microscopic structures of *Ma. jilinensis*. **A** Basidiomata. **B** Basidiospores. **C** Basidia and basidioles. **D** Cheilocystidia. **E** Pileipellis. Scale bars: 1 cm **(A)**; 15 μm **(B–E)**.

####### Etymology.

“jilinensis” refers to Jilin Province, China, where the type specimen was collected.

####### Description.

Basidiomata small to medium-sized. Pileus 0.4–1.4 cm diam, initially convex to parabolic, becoming obtusely conical at maturity; surface pale orange-yellow (RAL2000), smooth, slightly darker at the disc, orange (RAL2010); margin entire, not grooved; texture pliant and easily shriveling. Context very thin, white with a warm yellow tint (RAL1015); odor indistinct. Lamellae sinuate, distant, equal, narrow, 1–2 mm wide, *L* = 15, cream (RAL9001). Stipe 2–5 cm long, 0.5–1 mm thick, filamentous, equal; apex creamy white (RAL9001), middle pale reddish-brown (RAL3012) to reddish-brown (RAL8012), base olive-brown (RAL8011) to black (RAL9004); surface smooth; basal mycelium white (RAL9003).

Basidiospores (8.7) 8.9–11 (12) × (2.4) 2.9–3.2 (3.6) µm, *Q* = (2.58) 2.74–4.02 (4.29) [3.37 ± 0.39], lacrymoid, thin-walled, hyaline, inamyloid. Basidia 15–26 × 4.3–6.9 µm, clavate, thin-walled, hyaline, 4- (rarely 2-) sterigmate; sterigmata 1.8–4.8 µm long. Basidioles 15–25 × 3.8–6.9 µm, fusiform, apex with a blunt mucro, thin-walled, hyaline. Cheilocystidia *Siccus*-type, 8–15 × 4.2–8 µm; main body short-cylindrical when young, becoming long-cylindrical to long-clavate at maturity, thin-walled, hyaline; apex with 4–10 small spines, spines 1.7–4.7 µm long, hyaline to pale brown in 5% KOH. Pleurocystidia not observed. Lamellar trama irregular, interwoven; hyphae 3.5–7.3 µm wide, thin-walled, hyaline, dextrinoid. Pileipellis hymeniform, composed of *Siccus*-type cells, 7.4–14 × 4.3–13 µm; main body clavate, hyaline in 5% KOH; apex with 4–12 spines, spines 2.6–4.4 µm long, brown in 5% KOH. Pileus trama composed of irregular, interwoven, hyaline hyphae 3.5–5.5 µm wide. Stipitipellis composed of parallel, smooth, thick-walled, pale brown, dextrinoid hyphae. Clamp connections present in all tissues.

####### Typus.

CHINA • Jilin Province, Changchun City, Nanguan District, the campus of Jilin Agricultural University, on dead leaves of broad-leaved trees, 12 July 2023, 43°48'15"N, 125°25'10"E, alt. 243 m, S.E. Wang, FJAU69327 (E230797), holotype!.

####### Additional materials examined.

CHINA • Jilin Province, Changchun City, Nanguan District, Jingyuetan, on dead leaves of broad-leaved trees, 7 July 2024, 43°48'18"N, 125°29'02"E, alt. 348 m, Z.Q. Chen, FJAU69329 (Q247718). • Changchun City, Nanguan District, Jingyuetan, on dead leaves of broad-leaved trees, 7 July 2024, 43°48'05"N, 125°28'48"E, alt. 312 m, Q.R. Liu, FJAU69328 (R24709); • Changchun City, Nanguan District, Jingyuetan, on dead leaves of broad-leaved trees, 7 August 2026, 43°48'14"N, 125°28'49"E, alt. 365 m, W.N. Hou, FJAU69462 (H26070807).

####### Notes.

Phylogenetically, *Ma. jilinensis* forms a clade with two unidentified *Marasmius*. sequences, and this clade is sister to a group comprising *Ma. bambusiniformis*, *Ma. graminicola*, and *Ma. occultatus*. However, *Ma. bambusiniformis* differs by its umbilicate pileus center and a pileus surface covered with a pruinose layer (Singer 1976). *Ma. occultatus* differs by its finely tomentose pileus surface and a distinctly lignicolous habit (Takahashi 2000). *Ma. graminicola* is characterized by a pileus center that is slightly to strongly umbonate and a striate pileus surface with striations becoming more pronounced upon drying (Oliveira et al. 2020a).

*Ma. jilinensis* resembles *Ma. siccus* (Schwein.) Fr. and *Ma. brunneoaurantiacus* in possessing marasmioid basidiomata and an orange-toned pileus. *Ma. siccus* is widely distributed across China, whereas *Ma. brunneoaurantiacus* has been reported only from Shandong Province in China. *Ma. jilinensis* differs from *Ma. siccus*, which has free lamellae with a distinct collarium and pleurocystidia that are capitate-lageniform, lageniform, or subfusiform. *Ma. brunneoaurantiacus* was originally described from Madagascar by Antonín and Buyck (2006), based on the type specimen Buyck 99.450 collected from dead leaves of *Uapaca ferruginea* Baill. *Ma. brunneoaurantiacus* differs from the present species by its umbilicate pileus center, free lamellae, and ellipsoid basidiospores measuring 9.5–11 × 4.5–6 µm, with *Q* = 1.9–2.1 (Antonín and Buyck 2006; Shay et al. 2017). Although *Ma. crinipes* Antonín, Ryoo & H.D. Shin belongs to a different subsection, it nonetheless resembles this species in pileus color. *Ma. crinipes* can be distinguished by its pileus bearing a bluntly rounded center with fine wrinkles, while the remaining pileus surface shows conspicuous deep radial sulcations. Basidiospores of *Ma. crinipes* are elongate-clavate to fusiform (20–27 × 3.5–5.0 µm), and its cheilocystidia possess a rostrate or papillate apex, occasionally with an irregular morphology, and contain strongly refractive contents (Antonín et al. 2012b). By contrast, *Ma. jilinensis* has a smooth pileus center, with the pileus surface lacking striations or exhibiting only indistinct ones; its basidiospores are lacrymoid, measuring 9.8–11 × 2.9–3.2 µm, and its cheilocystidia are of the *Siccus*-type. *Ma. clocca* F.E. Guard, Dearnaley & T. Lebel is a recently described species that is morphologically close to *Ma. jilinensis* (Crous et al. 2026). Nevertheless, *Ma. clocca* has a shallowly sulcate pileus surface across its outer two-thirds, and its basidiospores (14.7–17 × 3.5–4 µm, *Q* = 3.64–4.64) are distinctly longer. In addition, its basidioles are narrowly fusoid to clavate and lack a blunt mucro at the apex, which distinguishes it from *Ma. jilinensis*.

###### Marasmius horqinensis

Taxon classificationFungiAgaricalesMarasmiaceae

T. Bau & W.N. Hou sp. nov.

C73AF13A-A287-5BA4-8454-65C83C3A918C

863743

Figs 4D, 4E, 7

####### Diagnosis.

It is similar to *Ma. elaeocephalus* but differs in having adnate, distant (*L* = 8–10, I = 1–2) lamellae and *Rotalis*-type cheilocystidia.

**Figure 7.**
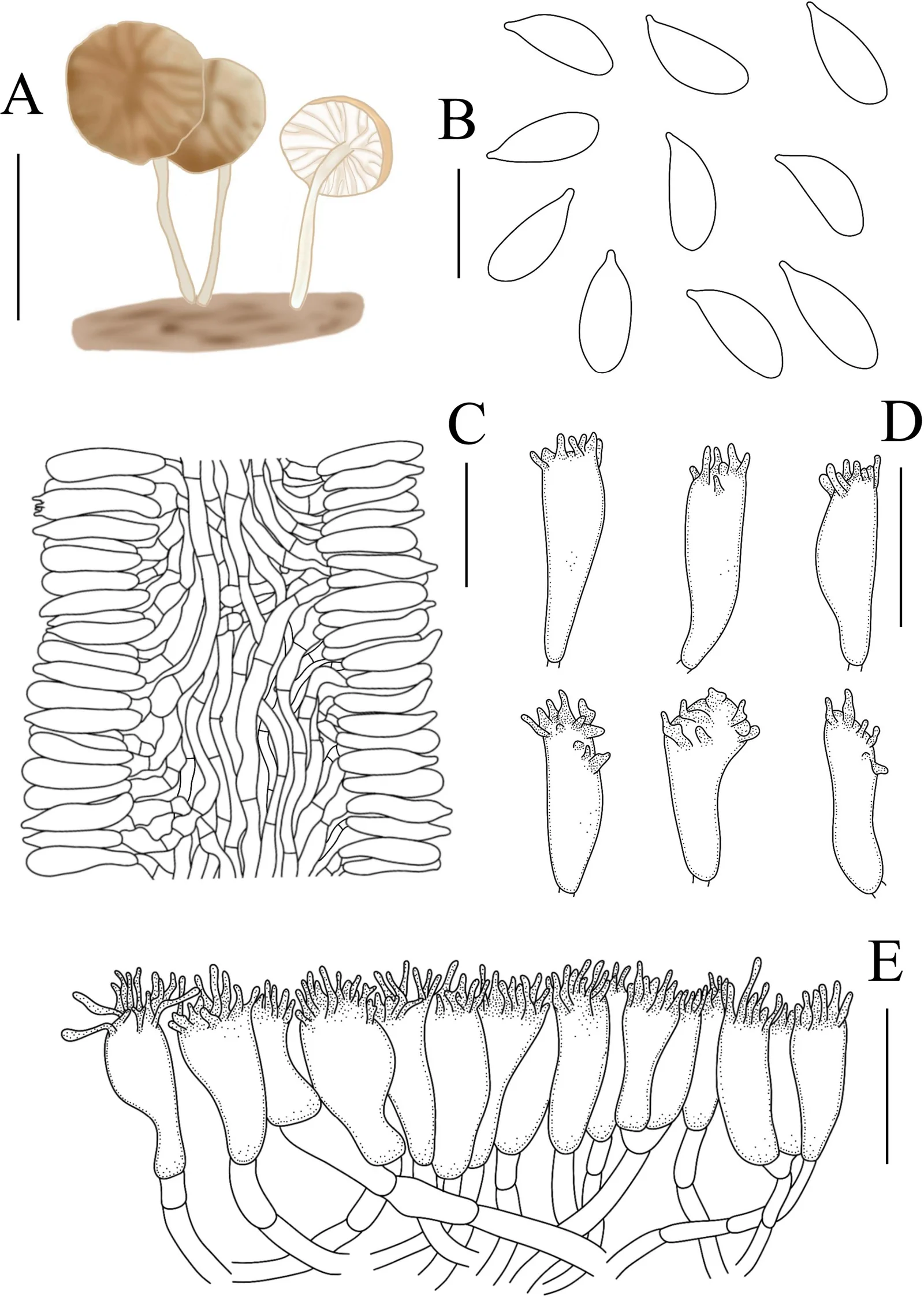
Microscopic structures of *Ma. horqinensis*. **A** Basidiomata. **B** Basidiospores. **C** Basidia, basidioles and lamellar trama. **D** Cheilocystidia. **E** Pileipellis. Scale bars: 0.5 cm **(A)**; 10 μm (**B)**; 15 μm (**C–E)**.

####### Etymology.

*“horqinensis”* refers to the type locality, Horqin Left Wing Rear Banner, Tongliao City, Inner Mongolia Autonomous Region, China.

####### Description.

Basidiomata small. Pileus 0.2–0.5 cm diam, initially plano-convex, becoming nearly plane at maturity; surface tough, smooth, cream-yellow (RAL1002) to ochre (RAL1024), with radial grooves, slightly depressed at center, often wrinkled in the depression; margin entire, straight. Context cream (RAL9001), very thin; odor indistinct. Lamellae adnate, distant, unequal, very narrow, 0.5–1 mm wide, *L* = 8–10, *I* = 1–2, cream (RAL9001). Stipe 0.3–1 cm long, 0.5–1 mm thick, cylindrical, equal or slightly thicker at base, cream (RAL9001), smooth, hollow, tough; base directly attached to habitat.

Basidiospores (8) 8–12 (13) × (3) 3.4–4.8 (5.2) µm, *Q* = (2.05) 2.12–3.02 (3.24) [2.52 ± 0.31], lacrymoid, thin-walled, hyaline, inamyloid. Basidia 16–26 × 4.2–9.4 µm, clavate, thin-walled, hyaline, 4- (rarely 2-) sterigmate; sterigmata 1.5–4.3 µm long. Basidioles 15–26 × 3.7–6.5 µm, mostly fusiform, occasionally clavate, thin-walled, hyaline. Cheilocystidia *Rotalis*-type, 12–24 × 4.5–8.3 µm; main body elongated-cylindrical to elongated-clavate; apex bearing 4–13 projections, projections 1.8–3.9 µm high. Pleurocystidia not observed. Lamellar trama irregular; hyphae 3.2–5.3 µm wide, thin-walled, hyaline, dextrinoid. Pileipellis hymeniform, composed of *Siccus*-type cells, 11–20 × 5.6–10 µm; main body spherical to broadly clavate; apex bearing 5–15 small spines, spines 3.4–5.7 µm long, pale brown in 5% KOH. Pileus trama composed of irregular, interwoven, thin-walled, hyaline, dextrinoid hyphae 3.2–4.9 µm wide. Stipitipellis composed of parallel, smooth, thin-walled hyphae 3.9–6.7 µm wide. Clamp connections present in all tissues.

####### Typus.

CHINA • Inner Mongolia Autonomous Region, Tongliao City, Horqin Left Wing Rear Banner, Nugustai Town, on dead branches of coniferous trees, 14 July 2022, 43°23'48"N, 122°24'53"E, alt. 311 m, F. Guo & T. Bau, FJAU69326 (gf241), holotype!.

####### Additional materials examined.

CHINA • Inner Mongolia Autonomous Region, Tongliao City, Horqin Left Wing Rear Banner, Nugustai Town, on dead branches of coniferous trees, 14 July 2022, 43°23'48"N, 122°24'53"E, alt. 311 m, F. Guo, FJAU69324 (B04). • Heilongjiang Province, Greater Khingan Range Prefecture, Huma County, Guqigu National Wetland Park, on dead branches of broad-leaved trees, 13 August 2024, 52°19'49"N, 124°43'10"E, alt. 358 m, H. Cheng, FJAU69325 (C2481401).

####### Notes.

It is most closely related to *Ma. falcatipes. Ma. falcatipes* is distinguished by its generally eccentric, cylindrical, equal stipe, which is curved to geniculate, and by its *Siccus*-type pileipellis, the main bodies of which are cylindrical to clavate, often lobed and diverticulate (Desjardin and Petersen 1989). *Ma. falcatipes* typically grows on senescent leaves or stems of broad-leaved trees, herbaceous vines, or *Poaceae* plants, whereas this species grows on dead branches of coniferous or broad-leaved trees or on decaying wood. And *Ma. elaeocephalus* occurs solitarily on woody debris of undetermined dicotyledonous trees within coastal secondary forests or cacao/banana groves and is known from Africa (Grace et al. 2019). *Ma. horqinensis* occupies a habitat similar to that of *Collybiopsis koreana* (Antonín, Ryoo & H.D. Shin) J. Sung Kim & Y.W. Lim. Nevertheless, *Co. koreana* can be readily differentiated during field surveys by its finely tomentose pileus, particularly at the center, with distinct radial rugulose-sulcate features (Antonín et al. 2010b; Kim et al. 2022). This new species is also closely related to *Ma. conchiformis*. However, *Ma. conchiformis* bears reniform to shell-shaped basidiomata, lacks a stipe, and produces clavate to naviculate or fusoid basidiospores measuring 8–11.8 × 3–5 µm (Oliveira et al. 2014).

##### Key to series of *Marasmius* subg. *Globulares* sect. *Globulares* subsect. *Globulares*

**Table:** 

1	Pleurocystidia absent or present as setae	2
–	Pleurocystidia present as well-developed cystidia, not setiform	4
2	Pileipellis composed of *Globulares*-type cells	**ser. *Wynnearum***
–	Pileipellis composed of *Siccus*-type cells	3
3	Setae present in the hymenium or pileipellis	**ser. *Cohaerentes***
–	Setae absent from both hymenium and pileipellis	**ser. *Hypochroides***
4	Pileus center deeply pigmented, margin somewhat translucent, gradually fading to a lighter color towards the edge	**ser. *Silvicolae***
–	Pileus surface uniformly pigmented, margin not translucent	**ser. *Brunneospermi***

###### Marasmius unicuspidatus

Taxon classificationFungiAgaricalesMarasmiaceae

T. Bau & W.N. Hou sp. nov.

97A57C69-75DC-5630-AF6D-31A0784A3291

863739

Figs 4F–I, 8

####### Diagnosis.

It is characterized by a pileus surface with faint longitudinal grooves, emarginate to sinuate lamellae, and mostly unbranched setiform cheilocystidia and pleurocystidia.

**Figure 8.**
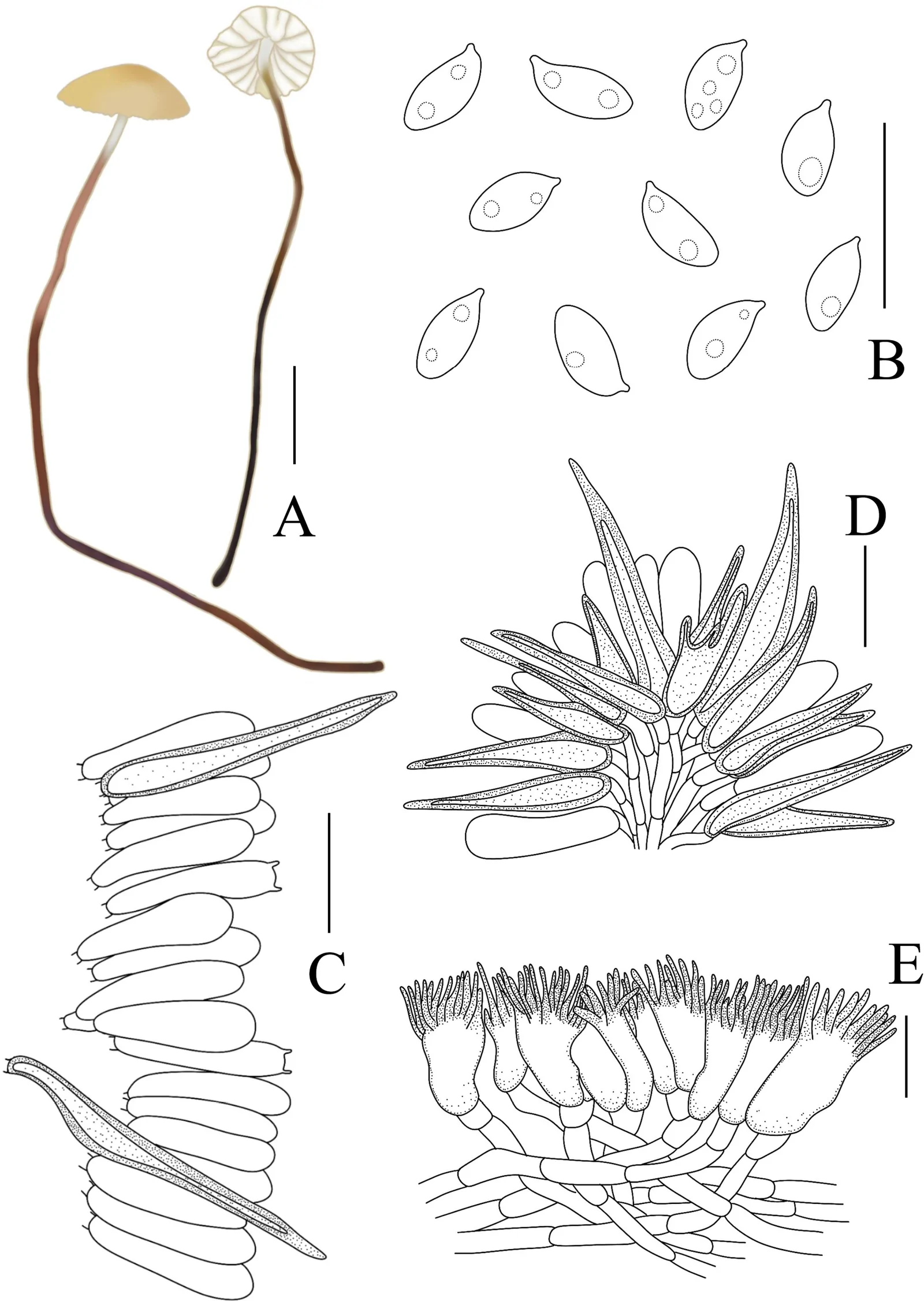
Microscopic structures of *Ma. unicuspidatus*. **A** Basidiomata. **B** Basidiospores. **C** Basidia, basidioles and setae. **D** Cheilocystidia. **E** Pileipellis. Scale bars: 0.5 cm **(A)**; 15 μm **(B–E)**.

####### Etymology.

“unicuspidatus” refers to the setiform cheilocystidia and pleurocystidia of this species, which are unbranched at the apex, with most bearing only a single pointed tip.

####### Description.

Basidiomata small. Pileus 0.6–0.8 cm diam., convex; surface pale orange-yellow (RAL1017), orange-brown (RAL8023) at the disc, smooth, with indistinct, shallow longitudinal grooves; margin entire; apex flat. Context thin, greyish-white (RAL1013); odor indistinct. Lamellae emarginate to sinuate, sparse, unequal, narrow, 1–1.5 mm wide, *L* = 10, *I* = 1–2, cream-colored, densely covered with ochre (RAL1024) tomentum; margin wavy. Stipe 4.5 cm long, 0.5–1 mm thick, cylindrical, slightly thicker at apex; apex greyish-white (RAL9002), middle pale orange-brown (RAL8023) to ochre-brown (RAL8001), base deep reddish-brown (RAL8012); hollow; surface smooth; base attached to habitat with a small amount of cream-colored (RAL9001) mycelium.

Basidiospores (5.6) 6.01–8.8 (8.9) × (3.1) 3.4–5.3 (5.3) μm, *Q* = (1.39) 1.45–1.95 (2.07) [1.69 ± 0.16], ovoid, smooth, thin-walled, hyaline, inamyloid. Basidia 18–25 × 4.8–6.3 μm, clavate, thin-walled, hyaline, 2-sterigmate; sterigmata 1.5–2.9 μm long. Basidioles 18–33 × 4.6–8.9 μm, clavate, smooth, thin-walled, hyaline. Cheilocystidia 28–43 × 3.2–6.3 μm, setiform, mostly unbranched, with a few bearing two or three apical branches, thick-walled, pale brown in 5% KOH. Pleurocystidia 19–60 × 5.1–11 μm, setiform, thick-walled, pale brown in 5% KOH. Lamellar trama irregular; hyphae 3.4–5.8 μm wide, thin-walled, hyaline, dextrinoid. Pileipellis hymeniform, composed of *Siccus*-type, 13–25 × 4.6–9.3 μm; main body clavate to elongate-cylindrical, apex bearing 3–17 small spines; spines 5.6–12 μm long, weakly dextrinoid. Pileus trama composed of interwoven, thin-walled, smooth, hyaline, dextrinoid hyphae 3.9–6.3 μm wide. Stipitipellis composed of parallel, slightly thick-walled, smooth hyphae 3.3–5.6 μm wide, pale yellowish-brown in 5% KOH. Clamp connections present in all tissues.

####### Typus.

CHINA • Heilongjiang Province, Harbin City, Shangzhi City, Mao’er Mountain, on decaying leaves of broad-leaved trees, 6 July 2025, 45°18'47"N, 127°33'21"E, alt. 302 m, W.N. Hou, FJAU69384 (H25070602), holotype!.

####### Additional materials examined.

CHINA • Jilin Province, Changchun City, Jingyuetan National Forest Park, on decaying leaves of broad-leaved trees, 20 July 2025, 43°46'56"N, 125°26'57"E, alt. 208 m, X.Y. Zhou, FJAU69385 (Y2572031).

####### Notes.

This species, together with *Ma. cohaerens* (Pers.) Cooke & Quél., belongs to *Marasmius* subg. *Globulares* sect. *Globulares* subsect. *Globulares* ser. *Cohaerentes*. Species within this series show a typical temperate distribution pattern. The series includes *Ma. cohaerens*, *Ma. cohaerens* var. *lachnophyllus* (Berk.) Gilliam, and *Ma. cohaerens* var. *mandshuricus* Kiyashko, E.F. Malysheva & Antonín. *Ma. cohaerens* var. *lachnophyllus* differs from the typical by the absence of caulocystidia, while *Ma. cohaerens* var. *mandshuricus* is recognized by its larger pleurocystidia (Kiyashko et al. 2014). *Ma. cohaerens*, a species widely distributed across China, differs from *Ma. unicuspidatus* in having a distinctly umbonate pileus with a tomentose surface, abundant setiform pileocystidia, and cheilocystidia and pleurocystidia that are mostly, and often profusely, branched. In contrast, *Ma. unicuspidatus* has a flat pileus apex and a smooth surface, lacks setiform pileocystidia, and possesses setiform cheilocystidia and pleurocystidia that are almost entirely unbranched at the apex (Cooke and Quélet 1878; Deng 2012). *Ma. delectans* Morgan is similar in overall macromorphology and basidiospore size, but differs through its predominantly North American distribution, brown-edged lamellae, and setiform cheilocystidia and pleurocystidia that are usually constricted one or more times toward the apex (Gilliam 1976). Furthermore, *Ma. unicuspidatus* differs from *Ma. crinipes*, another member of sect. *Globulares* widely distributed across the Northeast China Ecological Region. Specifically, *Ma. crinipes* possesses free or shortly adnexed lamellae that occasionally form a false collarium, together with clavate basidiospores (20–27 × 3.5–5.0 µm, *Q* = 5.0–6.1) (Antonín et al. 2012b).

###### Marasmius variicystidiatus

Taxon classificationFungiAgaricalesMarasmiaceae

T. Bau & W.N. Hou sp. nov.

2C47A064-5ACB-5B70-9CCA-D5CF5A71FD4A

863740

Figs 5D, 5E, 9

####### Diagnosis.

It is similar to *Ma. oreades*, but differs in having a thin, cream-colored context and a faintly sweet odor.

**Figure 9.**
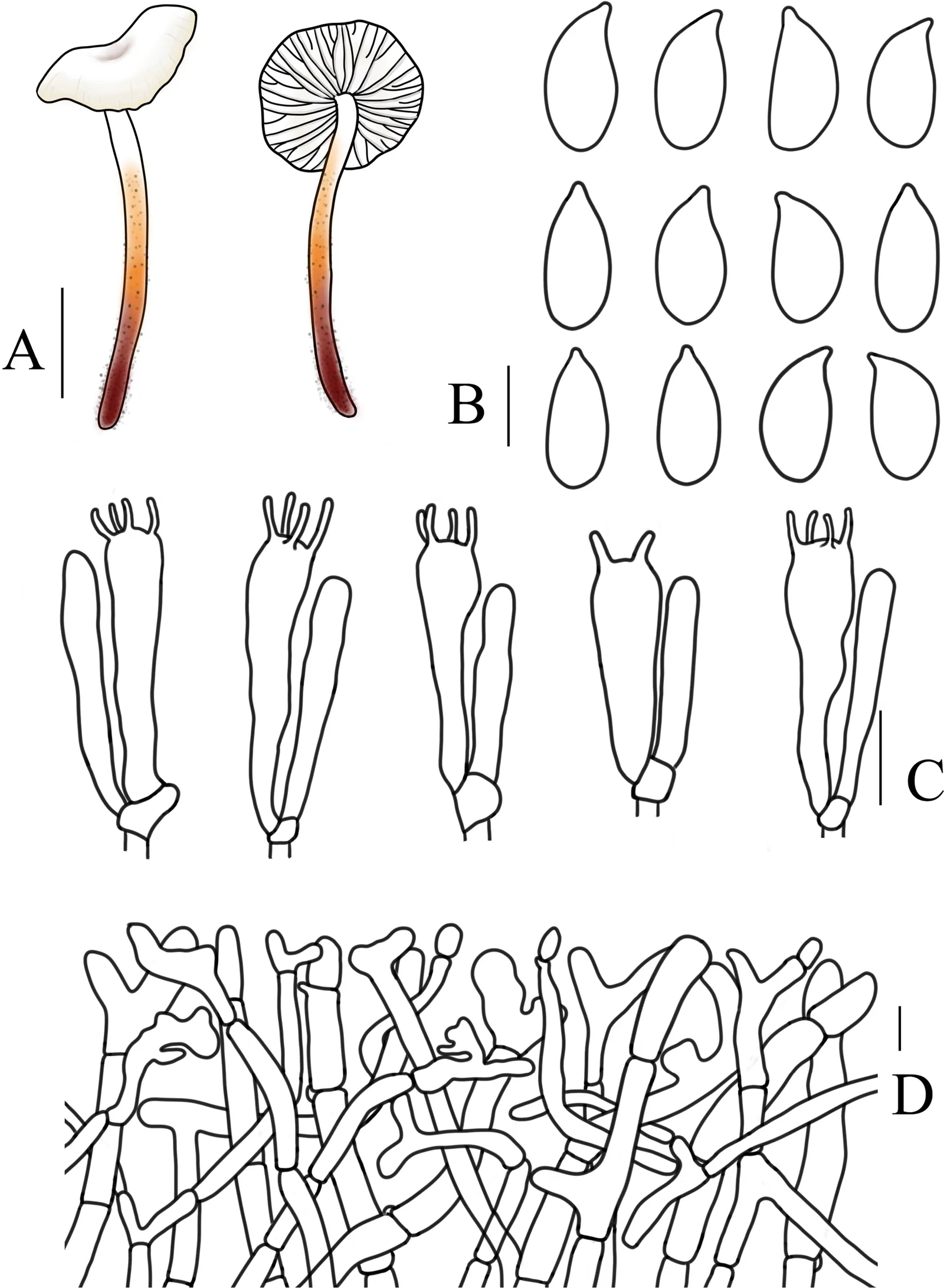
Microscopic structures of *Ma. variicystidiatus*. **A** Basidiomata. **B** Basidiospores. **C** Basidia and basidioles. **D** Pileipellis. Scale bars: 1 cm **(A)**; 10 μm **(B)**; 5 μm **(C, D)**.

####### Etymology.

“variicystidiatus” refers to pileipellis cells with irregular terminal branches.

####### Description.

Basidiomata small to medium-sized. Pileus 0.6–3 cm diam., initially plano-convex, becoming nearly plane with age, with a shallow central depression; surface white (RAL9003), smooth, pale brown (RAL8025) at center; margin slightly reflexed, membranous, shallowly grooved, becoming water-soaked, translucent, and distinctly striate when moist. Context cream (RAL9001), thin; odor faintly sweet. Lamellae sinuate to adnate, sparse, moderately broad, 2–3 mm wide, *L* = 20–23, *I* = 10–15, cream (RAL9001); edges smooth and entire. Stipe 3.6–6 cm long, 1–2 mm thick, subcylindrical, equal or tapering downwards, hollow, fibrous; apex cream (RAL9001) to milky white with a greyish tint (RAL7044), middle clay brown (RAL8001) to warm brown (RAL8003), base beige-brown (RAL8024) to reddish brown (RAL8012); surface covered with a cream-colored (RAL9001), pruinose mycelial layer; base attached to habitat with white (RAL9003) mycelium.

Basidiospores (6.7) 6.9–10.6 (10.9) × (2.5) 3.5–4.7 (4.7) µm, *Q* = (1.79) 1.82–2.54 (2.86) [2.15 ± 0.26], lacrymoid, smooth, thin-walled, hyaline, inamyloid. Basidia 23–30 × 3.5–6.1 µm, elongate-clavate, smooth, thin-walled, hyaline, 4-sterigmate, rarely 2-sterigmate, with a basal clamp connection. Cheilocystidia absent. Pleurocystidia absent. Lamellar trama irregular; hyphae weakly dextrinoid, with clamp connections. Pileipellis hymeniform, composed of *Globulares*-type cells, 4.8–23 × 2.5–9.2 µm, subglobose to ellipsoid, with irregular terminal branches, thin- to slightly thick-walled, inamyloid. Pileus trama composed of interwoven, cylindrical, dextrinoid hyphae 5.7–10.4 µm wide, with clamp connections. Stipitipellis hyphae 3.1–5 µm wide, elongate-cylindrical, pale yellow; subpellis hyphae parallel, 4–5.5 µm wide, cylindrical, yellowish brown to dark yellowish brown. Stipe trama hyphae parallel, 4.3–5 µm wide, cylindrical, hyaline. Caulocystidia absent. Clamp connections present in all tissues.

####### Typus.

CHINA • Inner Mongolia Autonomous Region, Tongliao City, Horqin Left Wing Middle Banner, Wusitu National Nature Reserve, on litter in broad-leaved forests, 10 July 2022, 43°47'47"N, 123°17'40"E, alt. 145 m, T. Bau & L. S. Mu, FJAU69373 (m05), holotype!.

####### Additional materials examined.

CHINA • Inner Mongolia Autonomous Region, Tongliao City, Horqin Left Wing Rear Banner, Yihuta, on decaying leaves of broad-leaved trees, 14 July 2022, 43°13'21"N, 122°24'58"E, alt. 222 m, Tolgor & W.N. Hou, FJAU69371 (H220787). • Tongliao City, Horqin Left Wing Rear Banner, Wudantala Forest Farm, on decaying leaves of broad-leaved trees, 16 July 2022, 42°59'55"N, 122°46'08"E, alt. 205 m, Tolgor & W.N. Hou, FJAU69443 (H2207154).

####### Notes.

It belongs to *Marasmius* subg. *Globulares* sect. *Globulares* subsect. *Globulares* ser. *Wynnearum*. *Ma. torquescens* Quél. and *Ma. wynneae* Berk. & Broome are also similar to *Ma. variicystidiatus*, sharing a shallowly grooved pileus margin and comparable stipe coloration. *Ma. torquescens* differs by bearing a blunt fulvous umbo at the pileus center (Quélet 1872). *Ma. wynneae* can be distinguished by its grayish-white pileus with a purplish tint and lamellae that also exhibit a purplish tint (Antonín et al. 2014a; Berkeley and Broome 1859).

*Ma. variicystidiatus* forms a distinct clade with strong support and shows a close relationship to *Ma. albogriseus* (Peck) Singer. The two species are largely similar in pileus shape, color, and marginal striations. However, *Ma. albogriseus* differs from the present species by having more distant lamellae, with whitish lamellae tinged orange-gray and conspicuous intervenose veins, and clavate pileipellis cells (Deng 2012). The stipe of *Ma. albogriseus* is paler in color, and its basidia are slightly shorter and broader relative to those of this species. In addition, *Ma. albogriseus* possesses polymorphic cheilocystidia, which may be cylindrical, subbasidioid, or irregularly inflated at one end, whereas such structures are not observed in the present species. Comparisons with its original description indicate that *Ma. wynneae* has a pale lilac-brown pileus and a hollow, nearly concolorous stipe, features that readily distinguish it from *Ma. variicystidiatus* (Berkeley and Broome 1859). The pileus and stipe colors reported for *Ma. wynneae* by Læssøe and Petersen (2019) differ from those given in the original description. The pileus has a grayish-white tone.

In general, early collections and descriptions of *Ma. oreades* were primarily concentrated in Europe and North America. Later, the distribution of this species was reported from many regions across the globe. Nevertheless, numerous researchers have found that the true taxonomic status of specimens identified as “*Ma. oreades*” is questionable. A large number of sequences deposited in the NCBI database under the name “*Ma. oreades*” are scattered across different phylogenetic lineages within *Marasmius*, creating substantial confusion for taxonomic studies of this genus. According to the most recent taxonomic revision, the true “*Ma. oreades*” should be circumscribed within *Marasmius* subg. *Globulares* sect. *Globulares* subsect. *Globulares* (Thorén et al. 2025). According to Oliveira et al. (2024a), *Ma. oreades* specimens (e.g., BUNS12-00735) from Europe (e.g., Denmark and Switzerland) and North America (e.g., the United States and Canada) may represent the core clade of true *Ma. oreades*, which may correspond to the typical evolutionary clade of this species in Europe and North America.

The species under discussion is easily confused with *Ma. oreades*. However, *Ma. oreades* differs in having a thick, pale-yellow context, a stipe that is white in the upper part and pale brown at the base, short clavate pileipellis cells, and an odor reminiscent of bitter almonds. Macroscopically, *Ma. variicystidiatus* can also be easily confused with *Ma. bellus* Berk. In contrast, *Ma. bellus* can be effectively distinguished by its pileipellis, which contains abundant *Siccus*-type cells (Oliveira et al. 2020b).

##### Key to series of *Marasmius* subg. *Globulares* sect. *Sicci*

**Table:** 

1	Pileus predominantly reddish in color, stipe subinsititious or with a scanty basal mycelium	**ser. *Haematocephali***
–	Pileus predominantly yellowish or brownish in color, stipe non-insititious, with a tomentose basal mycelium	**2**
2	Lamellae free, with a collar-like structure	**ser. *Sicci***
–	Lamellae free, without a collar-like structure	**ser. *Ferruginei***

###### Marasmius daqinggouensis

Taxon classificationFungiAgaricalesMarasmiaceae

T. Bau & H.Y. Bao sp. nov.

F0F5C863-955B-54D4-BB76-2003E65F48FD

863748

Figs 5F, 5K, 10

####### Diagnosis.

It differs from *Ma. castanocephalus* J.S. Oliveira in possessing more slender basidiospores and pleurocystidia with apices that are constricted or bear cylindrical projections.

**Figure 10.**
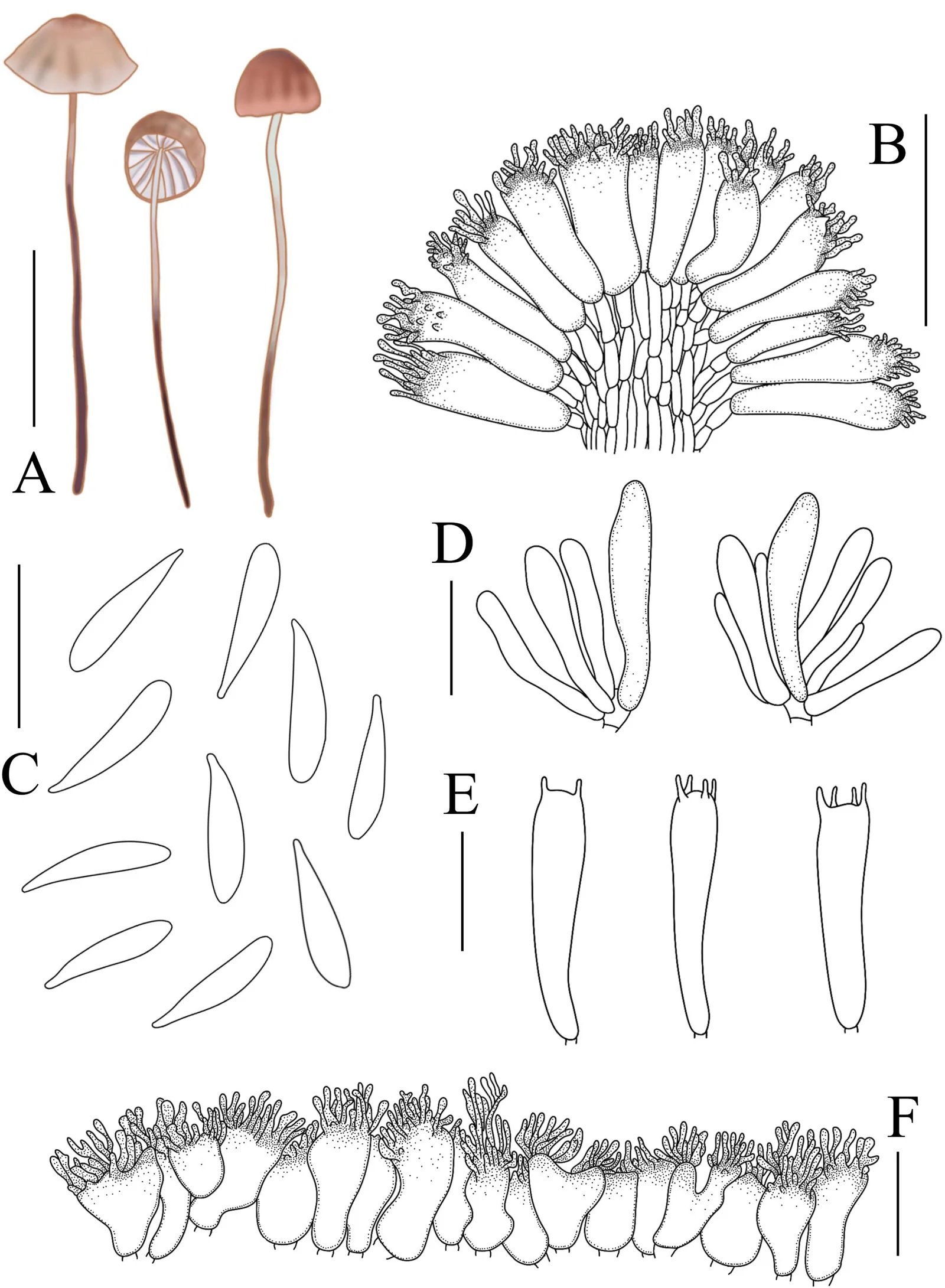
Microscopic structures of *Ma. daqinggouensis*. **A** Basidiomata. **B** Cheilocystidia. **C** Basidiospores. **D** Basidioles and pleurocystidia. **E** Pileipellis. Scale bars: 1 cm **(A)**; 15 μm **(B–E)**.

####### Etymology.

“daqinggouensis” refers to Daqinggou National Nature Reserve, where the type specimen was collected.

####### Description.

Basidiomata small. Pileus 0.2–0.8 cm diam., initially hemispherical to convex, becoming plano-convex at maturity, center slightly raised; surface pale reddish-brown (RAL3012), smooth, soft, fragile, with very shallow longitudinal grooves; margin white (RAL9003), initially involute, later slightly recurved. Context white (RAL9003), very thin; odor indistinct. Lamellae free, sparse, equal, *L* = 12, white (RAL9003) to cream (RAL9001), sometimes tinged pale reddish-brown (RAL3012). Stipe 1.4–3.6 cm long, less than 1 mm thick, filiform, nearly equal; apex grayish-white (RAL9002), translucent, middle gray (RAL7004), base olive-brown (RAL8008) to reddish-brown (RAL8012), darkening with maturity; surface smooth; base with white (RAL9003) mycelium attached to habitat.

Basidiospores (11) 12–15 (16) × (2) 2.4–3.5 (3.7) µm, *Q* = (3.78) 4.09–6.03(6.67) [4.90 ± 0.61], subfusiform, apex acute, base rounded, smooth, thin-walled, hyaline, inamyloid. Basidia 23–29 × 5.3–7.5 µm, elongate-clavate, thin-walled, hyaline, 4- (rarely 2-) sterigmate; sterigmata 2.2–5.8 µm long. Basidioles 20–26 × 4.8–6.6 µm, fusiform to clavate, thin-walled. Cheilocystidia *Siccus*-type, main body 6.4–10 × 3.2–7.7 µm, elongate-cylindrical; apex bearing 6–25 small spines, spines 2.3–3.9 µm long, pale brown in 5% KOH. Pleurocystidia 20–35 × 6–9.2 µm, main body narrowly cylindrical, with the apex constricted or bearing cylindrical projections, 3.1–8.3 µm long; thin-walled, refractive. Lamellar trama interwoven; hyphae 2.6–4.5 µm wide, thin-walled, hyaline, dextrinoid. Pileipellis hymeniform, composed of *Siccus*-type, main body 8–15 × 3.4–6.7 µm, subcylindrical to clavate; apex bearing 6–14 small spines, which are borne directly on the apical surface when projections or branches are absent, or on the 1–3 projections/branches when these are present; spines 1.6–8.5 µm long. Pileus trama composed of interwoven, weakly dextrinoid hyphae 2.4–3.8 µm wide. Stipitipellis composed of parallel, slightly thick-walled hyphae 3.8–5 µm wide.

####### Typus.

CHINA • Inner Mongolia Autonomous Region, Tongliao City, Horqin Left Wing Rear Banner, Daqinggou National Nature Reserve, on rotten wood of broad-leaved trees, 16 July 2023, 42°48'09"N, 122°10'23"E, alt. 220 m, T. Bau & H.Y. Bao, FJAU69391 (H2307121), holotype!.

####### Additional materials examined.

CHINA • Inner Mongolia Autonomous Region, Tongliao City, Horqin Left Wing Rear Banner, Daqinggou National Nature Reserve, 16 July 2023, 42°48'08"N, 122°10'22"E, alt. 223 m, on rotten wood of broad-leaved trees, T. Bau & W.N. Hou, FJAU69393 (H2307122). • Tongliao City, Horqin Left Wing Rear Banner, Daqinggou National Nature Reserve, on litter in broad-leaved forests, 42°48'03"N, 122°10'32"E, alt. 218 m, 16 July 2023, T. Bau & X. Y. Zhou, FJAU69392 (Y2371637).

####### Notes.

It is assigned to ser. *Haematocephali* based on its narrowly cylindrical pleurocystidia with short cylindrical projections. Historically, numerous specimens of this series collected worldwide were identified as *Ma. haematocephalus* (Mont.) Fr. Recent studies have confirmed that *Ma. haematocephalussensu lato* is not a single pantropical species, but a species complex consisting of several morphologically similar taxa (Oliveira et al. 2022). Within this complex, *Ma. haematocephalus* s.s. appears to be restricted to the Neotropics, while its closely related taxa are distributed in tropical Africa and the Indo-Malayan region. With its unique geographic location and extensive climatic gradient spanning tropical to temperate zones, China serves as a key region for exploring the diversity, distribution patterns, and evolutionary history of this species complex (Li et al. 2015). To date, only two species within this group have been recorded in China: *Ma. haematocephalus* and *Ma. asiaticus* Mešić & Tkalčec. No verified records of species in this group have yet been documented from the Northeast China Ecological Region.

*Ma. haematocephalus* is characterized by a purplish-red pileus with a flat center and a distinctly deeply sulcate surface, as well as cheilocystidia with clavate to obpyriform main bodies (Oliveira et al. 2022). In comparison, *Ma. daqinggouensis* has a pale reddish-brown pileus with extremely shallow striations or sulcations, and cheilocystidia with elongate-cylindrical main bodies. *Ma. asiaticus* is easily confused with *Ma. daqinggouensis* due to their similar pileus color. However, *Ma. asiaticus* produces elongate lacrymoid basidiospores, measuring 18.5–23 × 3.5–5 µm, (*Qm* = 5.5 ± 0.7), and pleurocystidia with fusoid to clavate main bodies. In contrast, *Ma. daqinggouensis* has subfusiform basidiospores measuring 13–14 × 2.7–2.9 µm and pleurocystidia with generally slender cylindrical main bodies (Tan et al. 2009). *Ma. fulvoferrugineus* Gilliam represents another morphologically similar species, but it can be differentiated by its yellowish-brown pileus with a broad central depression or truncation, a farinaceous odor, and longer basidiospores (Fries 1836). The phylogenetic tree reconstructed in this study indicates that *Ma. daqinggouensis* is most closely related to *Ma. aff. pallescens*. *Ma. aff. pallescens*, however, has a ruby-red pileus, a pale ruby interlamellar hymenium, and pleurocystidia that typically lack apical projections and constrictions. Moreover, when projections are present, they are mostly capitate (Oliveira et al. 2022). In addition, *Ma. daqinggouensis* has pleurocystidia with apices that are constricted or possess cylindrical projections. *Ma. crinipes* differs from *Ma. daqinggouensis* in having clavate basidiospores (20–27 × 3.5–5.0 μm, *Q* = 5.0–6.1) and fusiform, broadly cylindrical, or clavate pleurocystidia, with rostrate projections or small apical warts and refractive cytoplasmic contents (Antonín et al. 2012b). In general, *Ma. castanocephalus* J.S. Oliveira is mainly characterized by a buff brown to brownish beige pileus, such that it can be readily distinguishable from *Ma. daqinggouensis* in the field (Oliveira et al. 2022).

###### Marasmius coronatospinosus

Taxon classificationFungiAgaricalesMarasmiaceae

T. Bau & W.N. Hou sp. nov.

1CE949B1-54FD-58EE-BCD7-957D74C2BC15

863747

Figs 5A–C, 11

####### Diagnosis.

It is similar to *Ma. siccus*, but the latter has a golden-yellow pileus, a subcylindrical stipe, and pileipellis cells with subcylindrical main bodies bearing long, acute apical spines. However, *Ma. coronatospinosus* has a tomato-red pileus, a filiform stipe, and pileipellis cells with short clavate main bodies bearing short, blunt apical spinules arranged in a crown-like pattern.

**Figure 11.**
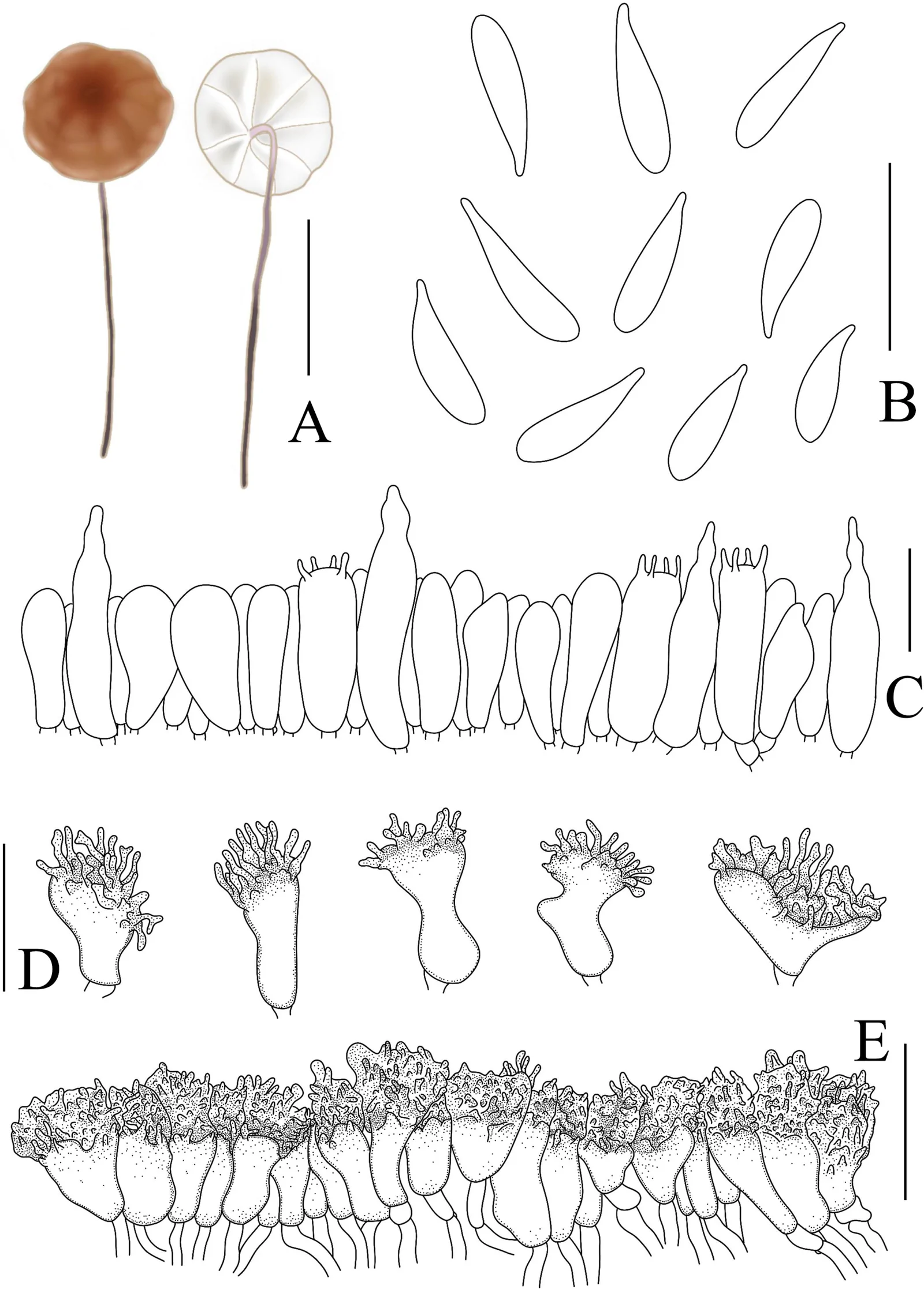
Microscopic structures of *Ma. coronatospinosus*. **A** Basidiomata. **B** Basidiospores. **C** Basidia, basidioles and pleurocystidia. **D** Cheilocystidia; **E** Pileipellis. Scale bars: 0.5 cm **(A)**; 15 μm **(B–E)**.

####### Etymology.

“coronatospinosus” refers to the short spines arranged in a crown-like pattern at the apex of the pileipellis cells.

####### Description.

Basidiomata small. Pileus 0.4–0.6 cm diam., initially convex to campanulate, becoming plano-convex at maturity, with a mammillate disc; surface smooth, glossy when wet, with shallow longitudinal grooves and a concentric bulge at the center; disc tomato-red (RAL3013), fading toward the margin to clay brown (RAL8001) to orange-brown (RAL8023); margin grayish-white (RAL1013), reflexed. Context off-white (RAL7047), very thin; odor indistinct. Lamellae free, with a collar-like structure, sparse, equal, 1–2 mm broad, *L* = 8, white (RAL9003), translucent and tinged brown (RAL7047) when wet. Stipe 1.6–3.2 cm long, 0.5 mm thick, filiform, equal or slightly thicker at apex; apex grayish-white (RAL1013), translucent, darkening downwards; middle clay brown (RAL8001) to olive-brown (RAL8008), base dark brown (RAL8011) to blackish-brown (RAL8022); surface smooth, glossy; base directly attached to habitat.

Basidiospores (10) 11–14 (14) × (2.5) 2.9–4.6 (4.9) µm, *Q* = (2.61) 2.83–4.2 (4.8) [3.47 ± 0.49], fusiform, smooth, thin-walled, hyaline, inamyloid. Basidia 21–26 × 5.8–8.9 µm, subcylindrical, equal in width, thin-walled, hyaline, 4- (rarely 2-) sterigmate; sterigmata 2.8–3.7 µm long. Basidioles 17–29 × 3.9–8.4 µm, clavate to fusiform, thin-walled, hyaline. Cheilocystidia 12–22 × 4.8–7.3 µm, with a subclavate main body, bearing 6–16 apical spines, 2.4–5.1 µm high. Pleurocystidia 24–36 × 7.3–10 µm, with subfusiform main body; apex apices with one or more constrictions, usually globose, moniliform with 1–3 similar elements in a chain, sometimes digitate; apical portion 2.9–14 µm long; smooth, thin-walled, hyaline, refractive or faintly refractive. Lamellar trama irregular; hyphae 3.2–3.8 µm wide, thin-walled, hyaline, weakly dextrinoid. Pileipellis hymeniform, composed of *Siccus*-type cells, main body 7.9–16 × 6–12 µm, short-clavate; apices unbranched or with 1–3 branches, bearing 9–17 short, blunt spinules, often adhering to one another and arranged in a crown-like pattern, thus difficult to count accurately; spines 2.4–4.9 µm long, pale brown. Pileus trama composed of interwoven, smooth, thin-walled, hyaline, weakly dextrinoid hyphae 3.3–4.8 µm wide. Stipitipellis composed of smooth, thick-walled, pale yellowish-brown hyphae 3.2–4.2 µm wide. Clamp connections present in all tissues.

####### Typus.

CHINA • Heilongjiang Province, Greater Khingan Mountains Region, Jiagedaqi District, roadside near National Highway G111, on dead grass, 28 July 2024, 50°17'17"N, 124°14'44"E, alt. 422 m, W.N. Hou, FJAU69397 (H2472818), holotype!.

####### Additional materials examined.

CHINA • Inner Mongolia Autonomous Region, Tongliao City, Naiman Banner, Mengjiaduan National Wetland Park, 43°22'23"N, 120°58'52"E, alt. 278 m, on dead grass, 1 August 2025, W.N. Hou, FJAU69399 (H25080102). • Inner Mongolia Autonomous Region, Tongliao City, Naiman Banner, Mengjiaduan National Wetland Park, 43°22'24"N, 120°58'52"E, alt. 278 m, on dead grass, 1 August 2025, W.N. Hou, FJAU69400 (H25080103).

####### Notes.

It belongs to *Marasmius* subg. *Globulares* sect. *Sicci*. It is assigned to ser. *Haematocephali* due to the moniliform apex of its pleurocystidia. It resembles *Ma. haematocephalus* s.s. and *Ma. asiaticus*, two species recorded in China, in possessing a reddish pileus. However, *Ma. haematocephalus* s.s. exhibits an incurved to straight pileus margin and pileipellis cells with apices that are partially capitate, papillate, or rarely short-mucronate. *Ma. asiaticus* features red lamellar edges and pleurocystidia morphologically similar to those of *Ma. haematocephalus* s.s. In contrast, the present species has a reflexed pileus margin and subfusiform pleurocystidia. Their apices usually bear one or more constrictions and are arranged in a moniliform manner, sometimes digitate (Mešić and Tkalčec 2010; Tan et al. 2009).

In the phylogenetic tree, *Ma. coronatospinosus* shows the closest relationship to *Ma. coasiaticus* J.S. Oliveira. *Ma. coasiaticus*, however, has a pileus with a flat to shallowly depressed center and pleurocystidia that are broadly clavate, rod-like to subovoid. It is noteworthy that the present species possesses a mammillate pileus center and subfusiform pileipellis cells. *Ma. coronatospinosus* is also phylogenetically related to *Ma. roseus* J.S. Oliveira, whereas *Ma. roseus* differs by having a grayish-pink to pale violet pileus with a flat or slightly rugulose center and pleurocystidia with apices devoid of projections or bearing only a single globose to short-mucronate projection (Oliveira et al. 2022).

*Ma. curreyi* Berk. & Broome and *Ma. coronatospinosus* both preferentially grow on dead grass leaves. However, *Ma. curreyi* can be distinguished by its orange-tinged pileus with a brown central umbo, free lamellae, and the presence of a collarium (Berkeley and Broome 1879; Antonín 2007).

##### Key to sections of *Marasmius* subg. *Marasmius*

**Table:** 

1	Pileus center slightly depressed or umbilicate	**2**
–	Pileus center umbilicate and the depression contains a central dot or papilla-like projection	**4**
2	Hymenium well-developed as lamellae	**3**
–	Hymenium lamella-like, with longitudinal ridges, sometimes reticulate	**sect. *Sicciformes***
3	Pileus pale orange to brown	**sect. *Crinis-equi***
–	Pileus white to strong colors, predominantly reddish-brown to purplish-brown	**sect. *Sanguirotales***
4	Pileipellis composed of *Siccus*-, *Rotalis*-, or *Chrysochaetes*-type cells	**sect. *Marasmius***
–	Pileipellis composed of *Siccus*-type cells	**sect. *Variabilispori***

##### Key to subsections of *Marasmius* subg. *Marasmius* sect. *Sicciformes*

**Table:** 

1	Basidiomata phlebioid	**subsect. *Neosessiles***
–	Basidiomata marasmioid	**2**
2	Lamellae with a collar or collar-like structure	**subsect. *Sicciformes***
–	Lamellae lacking a collar	**subsect. *Leveilleani***

##### Key to subsections of *Marasmius* subg. *Marasmius* sect. *Marasmius*

**Table:** 

1	Pileipellis primarily composed of *Chrysochaetes*-type cells	**subsect. *Horriduli***
–	Pileipellis not composed of *Chrysochaetes*-type cells, but composed of either *Rotalis*-type or *Siccus*-type cells	**2**
2	Pileipellis primarily composed of *Siccus*-type	**subsect. *Guyanenses***
–	Pileipellis primarily composed of *Rotalis*-type	**subsect. *Marasmius***

###### Marasmius clavocystidiatus

Taxon classificationFungiAgaricalesMarasmiaceae

T. Bau & W.N. Hou sp. nov.

6D723DA5-0B12-5725-8C81-358C6FB1D2D6

864478

Figs 4J–L, 12

####### Diagnosis.

It is characterized by a white pileus with tea-brown to deep reddish-brown protrusions and radial grooves, translucent from the rim of the central depression to the pileus margin when moist, cheilocystidia *Rotalis*-type, clavate, pileipellis cells *Siccus*- and *Rotalis*-type, cylindrical to clavate.

**Figure 12.**
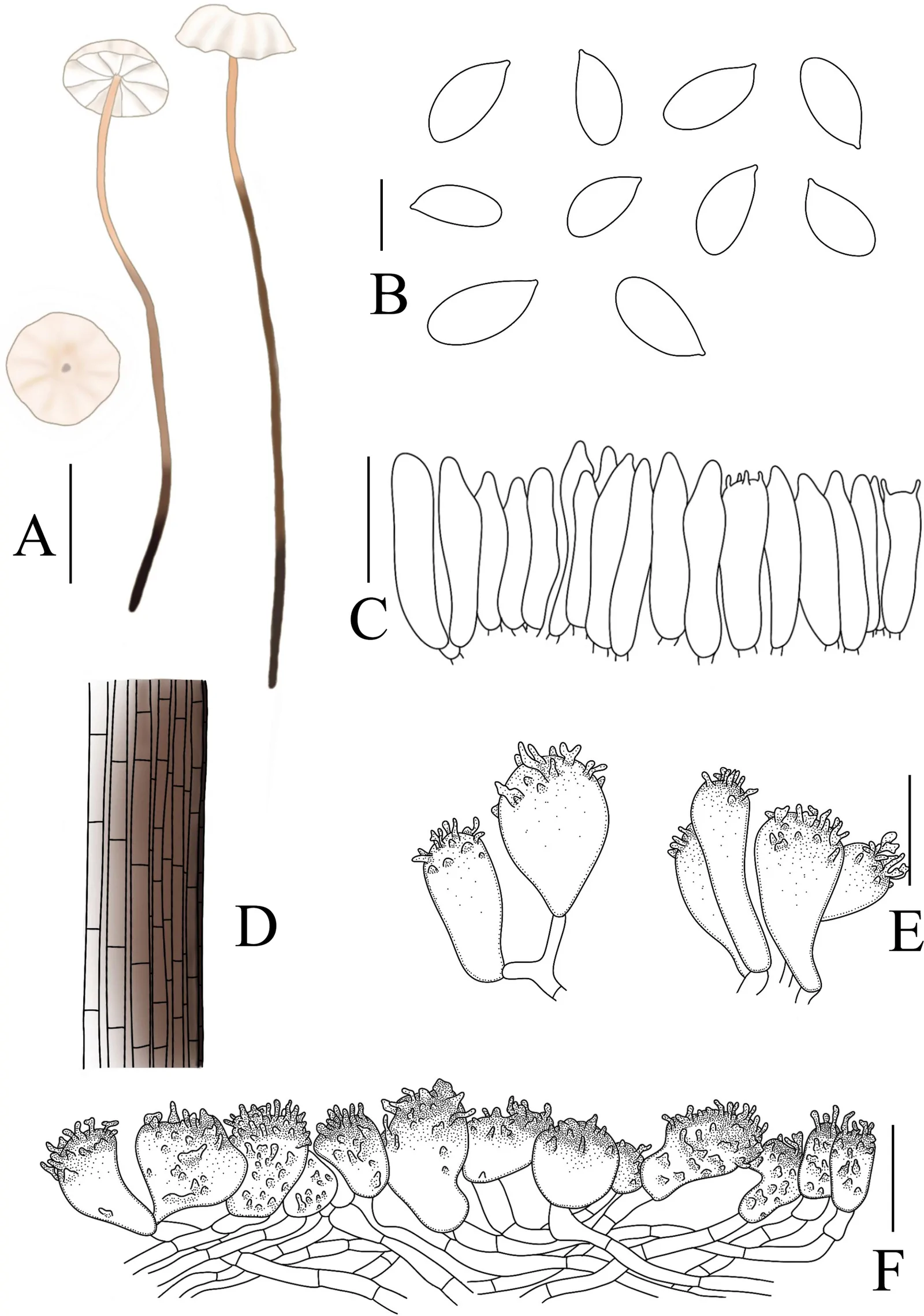
Microscopic structures of *Ma. clavocystidiatus*. **A** Basidiomata. **B** Basidiospores. **C** Basidia and basidioles. **D** Stipitipellis. **E** Cheilocystidia. **F** Pileipellis. Scale bars: 0.5 cm **(A)**; 5 μm **(B)**; 15 μm **(C–F)**.

####### Etymology.

“clavocystidiatus” refers to clavate cheilocystidia.

####### Description.

Basidiomata small. Pileus 4–7 mm diam., initially campanulate, becoming convex at maturity, apex truncate and centrally depressed, bearing a prominent, pointed papilla within the depression, surface white (RAL 1013), radially grooved, translucent from the rim of the central depression to the pileus margin when moist, papilla tea-brown (RAL 8003) to deep reddish-brown (RAL 8004), margin wavy. Context creamy white (RAL9001), thin; odor indistinct. Lamellae free, with a collar-like structure, sparse, equal, *L* = 10–12, dirty white. Stipe 0.8–1.7 cm long, 0.5 mm thick, filiform; entirely translucent when young; apex white (RAL9001) or nearly translucent at maturity and moist, middle pale brown (RAL3011) to blackish-brown (RAL3004), base black (RAL9004); surface smooth; base directly attached to habitat.

Basidiospores (6.5) 6.6–8.9 (8.9) × (3) 3.1–4.4 (4.5) µm, *Q* = (1.69) 1.88–2.44 (2.47) [2.13 ± 0.19], lacrymoid, smooth, hyaline, inamyloid. Basidia 17–23 × 4.3–5.9 µm, clavate, inamyloid, 4- (rarely 2-) sterigmate; sterigmata 2.3–3.1 µm long. Basidioles of two types: narrowly clavate, 14–22 × 2.6–4.9 µm, and narrowly fusiform, 22–28 × 3.4–4.8 µm, slightly protruding beyond the hymenium, smooth, thin-walled. Cheilocystidia *Rotalis*-type, main body 11–20 × 2.8–13 µm, clavate; surface bearing 10–25 projections concentrated at the apex; projections 2.1–4 µm high. Pleurocystidia absent. Lamellar trama irregular; hyphae 3.2–4.2 µm wide, inamyloid. Pileipellis hymeniform, composed of *Siccus*- and *Rotalis*-type cells, main body 8.4–20 × 8–13 µm, cylindrical to clavate, sometimes with a short basal stalk; projections 2–4.4 µm high, dark brown in 5% KOH, extremely dense to confluent, concentrated at the cystidial apex. Pileus trama composed of irregular, thin-walled, weakly dextrinoid hyphae 2.6–4.2 µm wide. Stipitipellis composed of regular, thick-walled hyphae 3.7–5.5 µm wide, dark brown in 5% KOH.

####### Typus.

CHINA • Heilongjiang Province, Greater Khingan Mountains Region, Jiagedaqi District, roadside on both sides of National Highway G111, on piles of dead grass in meadows, 28 July 2024, 50°19'05"N, 124°14'20"E, alt. 470 m, H.B. Song, FJAU69402 (S24072803), holotype!.

####### Additional materials examined.

CHINA • Heilongjiang Province, Greater Khingan Mountains Region, Jiagedaqi District, roadside on both sides of National Highway G111, on piles of dead grass in meadow, 28 July 2024, 50°19'10"N, 124°14'18"E, alt. 470 m, H. Cheng, FJAU69401 (C2472801). • Jilin Province, Changchun City, the campus of Jilin Agricultural University, 17 July 2024, 43°48'26"N, 125°25'12"E, alt. 229 m, T. Bau & H.Y. Bao, FJAU69403 (H247206). • Jilin Province, Changchun City, the campus of Jilin Agricultural University, on decaying leaf litter of broad-leaved trees, 16 June 2026, 43°48'48"N, 125°24'58"E, alt. 395 m, W.N. Hou, FJAU69455 (H26060013). • Jilin Province, Changchun City, the campus of Jilin Agricultural University, on decaying walnut fruits, 18 June 2026, 43°48'27"N, 125°25'00"E, alt. 234 m, X.Y. Zhou, FJAU69456 (Y2661810).

####### Notes.

It resembles *Ma. leucorotalis*, as both display a pileus with an umbilicate depression and a slightly elevated central papilla. However, the type specimen of *Ma. leucorotalis* (B 1414 LIL) is distinguished by a small black spot within the central umbilicate depression of the pileus, basidiospores measuring 7–7.8 × 2.7–3 μm, dextrinoid lamellar trama, and globose to subcystidioid *Rotalis*-type cheilocystidia and pileipellis cells (Singer 1965). Tan et al. (2009) described a Malaysian specimen identified as *Ma. leucorotalis* (TYS 486, SFSU), but this material deviates from the original description, most prominently in having inamyloid lamellar trama; thus, its identification requires further verification. In contrast, Brazilian specimens identified as *Ma. leucorotalis* largely conform to Singer’s (1976) description, with only minor differences in basidiospore size. Consistent with the examined specimens, the Malaysian specimen TYS 486 (SFSU) also exhibits inamyloid lamellar trama. It differs, however, in the morphology of both cheilocystidia and pileipellis cells. Furthermore, the available sequences of the Malaysian specimen are of poor quality, preventing reliable comparisons with the sequences generated from the specimens examined in this study. Sequences from Brazilian *Ma. leucorotalis* specimens were included in the analyses. These specimens feature a white to grayish-white depressed area at the pileus center when fresh, while the region outside the depression is grayish brown to pale gray-brown, darkening and ultimately turning brown upon drying. They also have dextrinoid lamellar trama, with pyriform, vesiculose, subglobose to globose cheilocystidia and pileipellis cells. In comparison, *Ma. clavocystidiatus* presents a white pileus surface with a tea-brown to deep reddish-brown papilla, inamyloid lamellar trama, *Rotalis*-type clavate cheilocystidia, and cylindrical to clavate *Siccus*- and *Rotalis*-type pileipellis cells. These morphological differences clearly separate this new species from *Ma. leucorotalis* (Oliveira et al. 2020b). In addition, the difference between *Ma. clavocystidiatus* and *Ma. rotalis* Berk. & Broome should be considered. *Ma. rotalis* is an early-described species with relatively limited morphological information in its protologue, and numerous distinct taxa have historically been assigned to this name. Comparison with the original description indicates that *Ma. clavocystidiatus* differs from *Ma. rotalis* primarily in pileus surface characteristics: *Ma. rotalis* has a pileus covered with dark brown pruina, whereas *Ma. clavocystidiatus* has a white pileus surface bearing a tea-brown to deep reddish-brown papilla (Berkeley and Broome 1873).

In the phylogenetic tree, specimens FJAU69402, FJAU69401, and FJAU69403 clustered together within a distinct major clade with strong support (BS = 99%, UFBoot = 1). A certain degree of genetic distance was still observed between FJAU69402 and the other two specimens, FJAU69401 and FJAU69403. Sequence alignment suggested that the variation observed in FJAU69402 may be attributable to intraspecific polymorphism, a phenomenon that appears relatively common in fungi (Cedeño-Sanchez et al. 2024).

Because the basidiomata of FJAU69403 were already dry at the time of collection, some key morphological characters may have been lost. For this reason, FJAU69403 was not designated as the type specimen; instead, the better-preserved specimen FJAU69402 was selected as the holotype of *Ma. clavocystidiatus*.

###### Marasmius maoershanensis

Taxon classificationFungiAgaricalesMarasmiaceae

T. Bau & W.N. Hou sp. nov.

9ECC15FB-B57E-5E94-93E2-EE25723BEEDE

863753

Figs 5G–J, 13

####### Diagnosis.

It is characterized by a grayish-white pileus with a black central depression and shallow or indistinct grooves, lacrymoid basidiospores, and *Rotalis*-type cheilocystidia and pileipellis cells.

**Figure 13.**
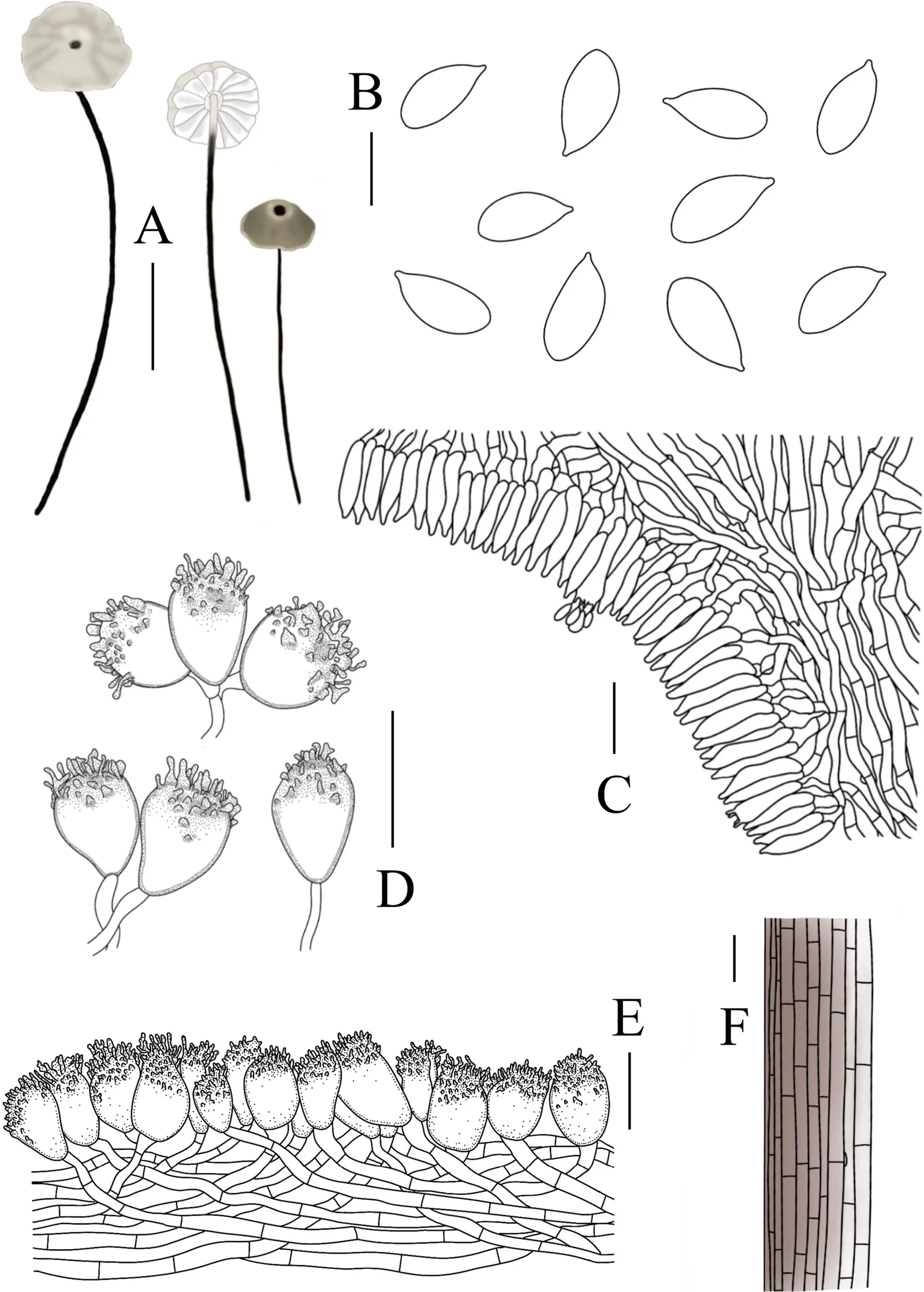
Microscopic structures of *Ma. maoershanensis*. **A** Basidiomata. **B** Basidiospores. **C** Basidia and basidioles. **D** Cheilocystidia. **E** Pileipellis. **F** Stipitipellis. Scale bars: 0.5 cm **(A)**; 5 μm **(B)**; 15 μm **(C–F)**.

####### Etymology.

“maoershanensis” refers to the type locality, Mao’er Mountain, Shangzhi City, Harbin City, Heilongjiang Province, China.

####### Description.

Basidiomata small. Pileus 0.3–0.6 cm diam., initially subconical, becoming plano-convex to nearly plane; center with a distinct umbilicate depression; depression margin grayish-white (RAL7044), slightly raised and forming a ring; depression interior black (RAL9004); surface initially dark gray (RAL7045), becoming pale gray (RAL7035), roughened, with shallow or indistinct grooves; margin straight to slightly upturned. Context white (RAL9003), very thin. Lamellae free, with a collar-like structure, sparse, equal, narrow, 1–2 mm broad, L = 12–15, white (RAL9003). Stipe 1–2.4 cm long, 0.5 mm thick, filiform; surface black (RAL9004), nearly smooth; base directly attached to habitat with black (RAL9004) rhizomorphs, some rhizomorph apices dark gray (RAL7047).

Basidiospores (5.2) 5.8–7.2 (7.5) × (2.6) 2.8–4 (4.2) µm, *Q* = (1.61) 1.61–2.34 (2.5) [1.87 ± 0.22], lacrymoid, thin-walled, hyaline, inamyloid. Basidia 15–23 × 4.6–6.9 µm, short-clavate, smooth, thin-walled, hyaline, 4- (rarely 2-) sterigmate. Basidioles 16–20 × 5–6.8 µm, subfusiform, with acute apex, smooth, thin-walled, hyaline. Cheilocystidia *Rotalis*-type, 11–17 × 5.3–14 µm; main body spherical to short-clavate, hyaline; apex bearing 6–12 short spines, spines 3.5–4.5 µm long, pale brown. Pleurocystidia not observed. Lamellar trama interwoven; hyphae 3.8–5.4 µm wide, thin-walled, hyaline, weakly dextrinoid. Pileipellis hymeniform, composed of *Rotalis*-type cells, 12–20 × 9–15 µm; main body subglobose, hyaline to brown; apex bearing 7–10 small spines, spines 1.4–2.7 µm long, yellowish-brown. Pileus trama composed of interwoven, smooth, thin-walled, hyaline, dextrinoid hyphae 3–3.9 µm wide. Stipitipellis composed of smooth, thick-walled, dark brown hyphae 4–4.8 µm wide. Clamp connections present in all tissues.

####### Typus.

CHINA • Heilongjiang Province, Harbin City, Shangzhi City, Mao’er Mountain, on rotten leaves in broad-leaved forests, 5 July 2025, 45°18'46"N, 127°33'18"E, alt. 300 m, W.N. Hou, FJAU69415 (H25070510), Holotype.

####### Additional materials examined.

CHINA • Inner Mongolia Autonomous Region, Tongliao City, Horqin Left Wing Rear Banner, Daqinggou National Nature Reserve, on rotten leaves in broad-leaved forests, 16 July 2023, 42°48'06"N, 122°10'28"E, alt. 229 m, T. Bau & Q.R. Liu, FJAU69416 (R23779). Inner Mongolia Autonomous Region, Tongliao City, Horqin Left Wing Rear Banner, Daqinggou National Nature Reserve, on rotten leaves in broad-leaved forests, 16 July 2023, 42°48'02"N, 122°10'36"E, alt. 222 m, T. Bau & W.N. Hou, FJAU69414 (H230790). Jilin Province, Changchun City, Jingyuetan National Forest Park, on rotten leaves in broad-leaved forest, 12 July 2026, 43°45'31"N, 125°28'11"E, alt. 309 m, W.N. Hou, FJAU69457 (H26070802). Jilin Province, Changchun City, Jingyuetan National Forest Park, on rotten leaves in broad-leaved forest, 12 July 2026, 43°45'31"N, 125°28'10"E, alt. 309 m, W. Sun, FJAU69458 (SW26070801).

####### Notes.

*Ma. maoershanensis* and *Ma. capillaris* Morgan both present a convex pileus with an umbilicate depression. However, the pileus of the present species is uniformly pale gray to grayish-white, whereas that of *Ma. capillaris* is pale brown with a grayish-white central area. Furthermore, the grooves on the pileus surface of this species are shallow or indistinct, differing from the prominent, deep grooves of *Ma. capillaris* (Harper et al. 1883; Gilliam 1976; Mustafabayli and Aghayeva 2019). This species is closely related to *Ma. tubulatus* and *Ma. wisteriae*. It differs from *Ma. tubulatus*, which has a pileus distinctly radially sulcate toward the center and pruinose, a stipe surface with fine longitudinal striations, and larger basidiospores (7–9 × 3–4 µm) (Petch 1947). *Ma. wisteriae* can be readily distinguished by its brownish-orange to whitish pileus (Antonín et al. 2014b). In addition, the two species exhibit clear differences in micromorphology. *Ma. wisteriae* produces clavate to fusiform basidiospores up to 14 μm in length, whereas the present species has lacrymoid basidiospores with a maximum length of only 7.5 μm. This species can be readily confused with *Ma. rotula* (Scop.) Fr., which differs by possessing 12 prominent grooves on the pileus surface (Fries 1838).

#### Crinipellis

##### Crinipellis macrospora

Taxon classificationFungiAgaricalesMarasmiaceae

(Antonín, Ryoo & H.D. Shin) T. Bau & W.N. Hou stat. nov.

7E946BEE-F91F-5EE6-B68D-BCDE516CF5E3

863756

Figs 14A–C, 15

###### Basionym.

Crinipellis nigricaulis var. macrospora Antonín, Ryoo & H.D. Shin, Mycotaxon 108: 431. 2009.

**Figure 14.**
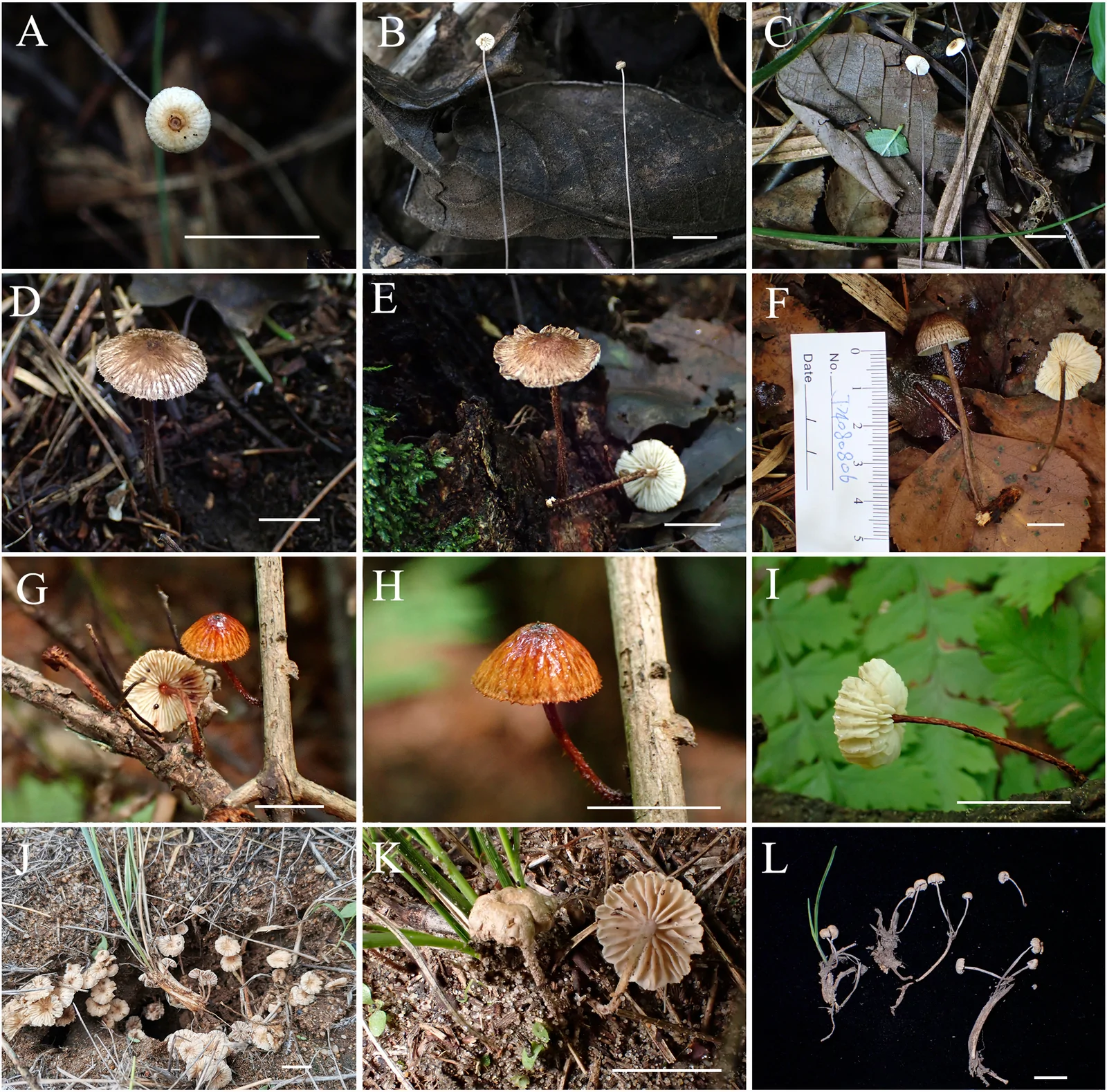
Basidiomata of *Crinipellis* and *Moniliophthora* species. *Cr. macrospora*, **A, C** FJAU69421 (J24071308); **B** FJAU69422 (H2470708). *Cr. fuscosquamosa*, **D** FJAU69428 (ZTY249619), holotype!; **E** FJAU69429 (ZTY249516); **F** FJAU69460 (J24080806). *Cr. xiaoxinganlingensis* nom. prov. **G** FJAU69447 (H2307344). **H** I FJAU69447 (H23073442). *Mo. vaginicola*. **J** FJAU69435 (B23005). **K** FJAU69463 (H230758), Holotype!. **L** FJAU69464 (H2307166). Scale bars: 1 cm (**A–F, J–L**), 0.5 cm (**G–I**).

**Figure 15.**
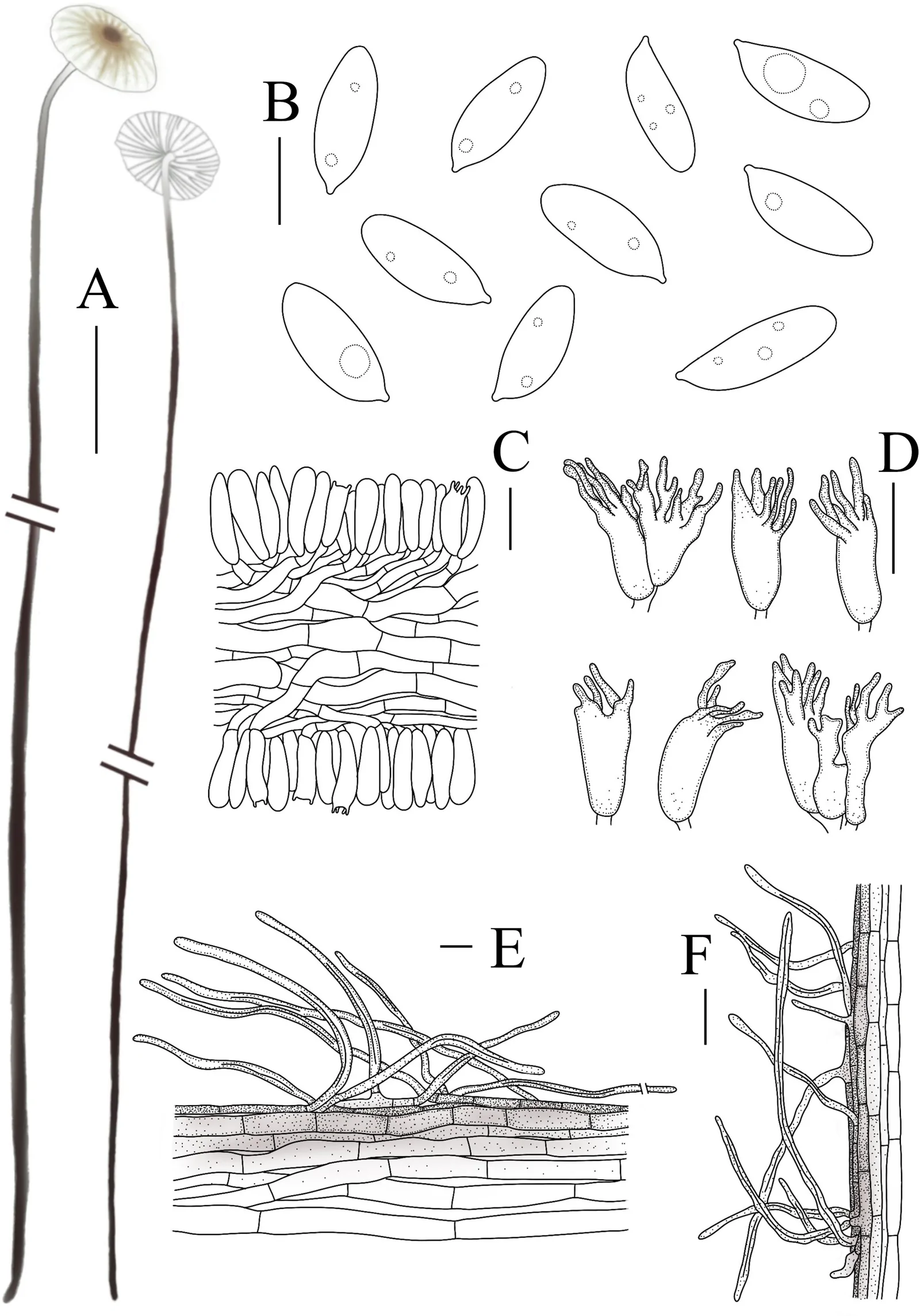
Microscopic structures of *Cr. macrospora*. **A** Basidiomata. **B** Basidiospores. **C** Basidia and basidioles. **D** Cheilocystidia. **E** Pileipellis. **F** Stipitipellis. Scale bars: 0.5 cm **(A)**; 5 μm **(B)**; 15 μm **(C–F)**.

###### Description.

Basidiomata small. Pileus 0.4–1.1 cm diam., convex, centrally depressed and disc-shaped, with a single protuberance in the disc; surface cream (RAL9001), with shallow grooves at the margin, covered with beige-brown (RAL8024), tomentose scales arranged radially; central depression brown (RAL8002), lacking concentric zones. Context cream (RAL9001), thin; odor indistinct. Lamellae free, moderately dense, unequal, *L* = 18–20, *I* = 1–2, cream (RAL9001). Stipe 4–15 cm long, 0.1 cm thick, central, extremely long, cylindrical, equal; base blackish-brown (RAL8022), gradually lightening upwards, apex cream (RAL9001), slightly enlarged; surface minutely tomentose; base with rhizomorphs.

Basidiospores (7) 7–9.5 (10.3) × (3.1) 3.5–4.9 (5.8) µm, *Q* = (1.55) 1.79–2.44 (3) [2.07 ± 0.27], oblong-ellipsoid, smooth, thin- to slightly thick-walled, hyaline, containing oil droplets, inamyloid. Basidia 15–26 × 3.4–7 µm, clavate, thin-walled, hyaline, 4- (rarely 2-) sterigmate; sterigmata 1.4–4.3 µm long, inamyloid. Basidioles 17–25 × 4–6.8 µm, clavate to fusiform, thin-walled, hyaline, inamyloid. Cheilocystidia clustered, 14–22 × 4–8.6 µm; main body subcylindrical to clavate, thin-walled, hyaline; apex bearing 4–6 branches, branches 4–12 × 1–3 µm. Pleurocystidia not observed. Lamellar trama regular. Pileus trama a cutis, brown in water, unchanged in 5% KOH; terminal cells dextrinoid. Pileus hairs 136–375 × 6–9 µm, cylindrical, setose, thick-walled, dextrinoid, brown in water, unchanged in 5% KOH, with rare and inconspicuous secondary septa. Stipe hairs 65–299 × 2.6–5.4 µm, cylindrical, setose, thick-walled. Stip trama composed of cylindrical cells 3.7–6.9 µm wide, appearing emerald green in KOH. Clamp connections present.

###### Typus.

Republic of Korea • Chuncheon, Dongsan-myeon, Bongmyeong-ri, 22.VI1I.2007, leg. V. Antonin 07.96 et R. Ryoo (holotypus in herbario BRNM 712569 preservatur).

###### Materials examined.

**CHINA** • Jilin Province, Jilin City, Huadian City, Hongshi National Forest Park, on leaf litter in broad-leaved forests, 27 August 2023, 42°49'44"N, 127°08'49"E, alt. 465 m, T. Bau & S.Y. Li, FJAU69423 (L23082707). • Jilin Province, Baishan City, Fusong County, Songjianghe National Forest Park, on leaf litter in broad-leaved forests, 13 July 2024, 42°10'17"N, 127°30'52"E, alt. 762 m, J. L. Wei, FJAU69421 (J24071308). • Liaoning Province, Dalian City, Zhuanghe City, Tianmenshan National Forest Park, on leaf litter in broad-leaved forests, 7 July 2024, 40°09'51"N, 122°54'05"E, alt. 267 m, W.N. Hou, FJAU69422 (H2470708).

###### Notes.

Based on the phylogenetic tree constructed in this study, sequences of the collected specimens FJAU69421, FJAU69422 and FJAU69423 cluster with those of Cr. nigricaulis var. macrospora. Cr. nigricaulisvar. macrospora was described by Antonín et al. based on the holotype BRNM 712569 (Korea) (Antonín et al. 2009). According to the original description, Cr. nigricaulis var. macrospora is characterized by a small pileus that is entirely brown to dark brown and later whitish at the margin, a very long filiform stipe, and moderately large basidiospores [8.5–11.5 × 3.75–4.75 (–5.0) µm], as well as clavate, (sub)cylindrical to subfusoid cheilocystidia bearing digitate projections up to 8 (–10) × 2.0 µm with obtuse to subacute apices (Antonín et al. 2009). Overall, the examined material aligns closely with the original description in all observed morphological characters. However, the KOH reaction of pileus hairs exhibits minor differences between the two groups. The pileus hairs of the examined specimens remain brown in 5% KOH. The hairs do not turn green. However, the original description reported an olive-green color change in pileus hairs in KOH. The cause of the aforementioned discrepancy requires further investigation. Combined with phylogenetic evidence, these specimens are deemed conspecific.

Antonín et al. (2009) stated that Cr. nigricaulis var. macrospora (*E* = 2.2–3.0, *Q* = 2.4–2.6, cheilocystidia 12–20 × 4–7 μm) differs from Cr. nigricaulisvar. nigricaulis (*E* = 1.6–2.2, *Q* = 1.8–1.9, cheilocystidia 15–23 × 6–11 μm) (Takahashi 2000) primarily in the *E* and *Q* values of the basidiospores and the width of the cheilocystidia. Here, *E* represents the quotient of length and width of an individual spore. Beyond these documented differences, reevaluation of Takahashi’s original description of *Cr. nigricaulis*, combined with the material examined in the present study, reveals several additional morphological characters that support their taxonomic separation. *Cr. nigricaulis* possesses a slightly umbilicate pileus center, radially fibrillose-strigose surface scales along plicate ridges, and pileus hairs with secondary septa that form scalariform structures. Phylogenetic analysis further demonstrates that Cr. nigricaulis var. macrospora and *Cr. nigricaulis* form two distinct, well-supported clades. Therefore, the elevation of Cr. nigricaulis var. macrospora to species rank is validated (Takahashi 2000). This species is sister to *Cr. cupreostipes*. Morphologically, this species (stipe hairs 65–299 µm long) differs from(stipe hairs 72–160 µm long, as reported in the protologue) in the length range of the stipe hairs (Kerekes and Desjardin 2009; Dutta et al. 2015). Although their ranges partially overlap, this character can currently be used to distinguish the two species. Further evidence is needed to identify and evaluate additional diagnostic characters.

##### Crinipellis fuscosquamosa

Taxon classificationFungiAgaricalesMarasmiaceae

T. Bau & W.N. Hou sp. nov.

5D1E27EC-11AC-585D-A211-B5F6874C1D5E

863760

Figs 14D–F, 16

###### Diagnosis.

This species differs from *Cr. bidens* T. Bau, in having a pileus with a dark brown, radially arranged, tomentose scales, stipe hairs with fusiform, ellipsoid, or subglobose swellings, and black-brown rhizomorphs at the stipe base.

**Figure 16.**
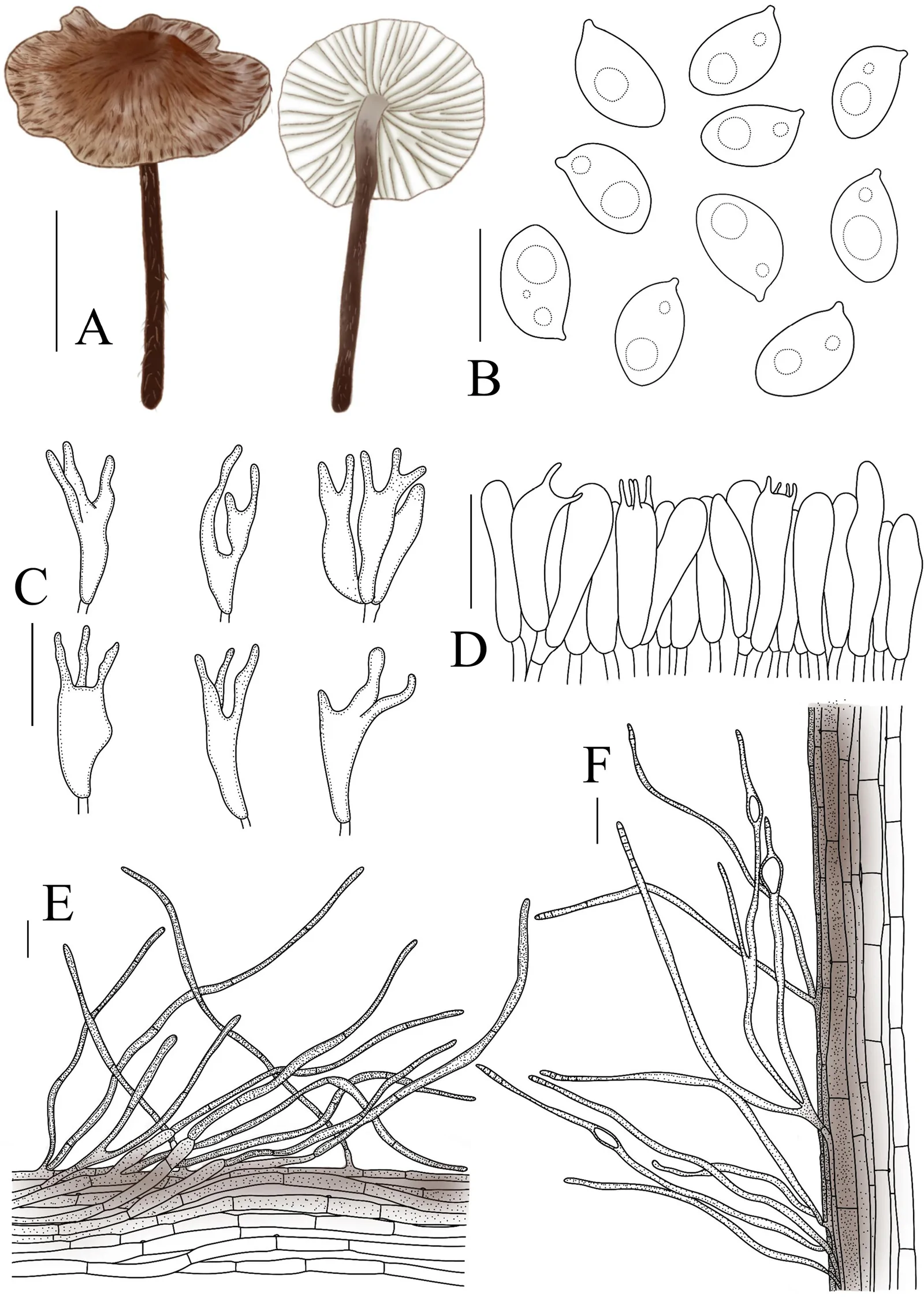
Microscopic structures of *Cr. fuscosquamosa*. **A** Basidiomata. **B** Basidiospores. **C** Cheilocystidia. **D** Basidia and basidioles. **E** Pileipellis. **F** Stipitipellis. Scale bars: 5 cm **(A)**; 5 μm **(B)**; 15 μm **(C–F)**.

###### Etymology.

“fuscosquamosa” refers to the brown scales densely covering the pileus and stipe surfaces.

###### Description.

Basidiomata medium-sized. Pileus 1.7–1.9 cm diam., initially subhemispherical, becoming applanate, center slightly raised; surface brown (RAL8002), with dark brown (RAL8011), entire scales at center and radially arranged, tomentose scales toward the margin. Context cream (RAL9001), thin. Lamellae free, dense, cream (RAL9001). Stipe 5.4–5.9 cm long, 1–2 mm thick, cylindrical, hollow; surface clay brown (RAL8001), densely covered with fibrous, dark brown (RAL8011) scales; base blackish-brown (RAL8022), slightly enlarged; with black-brown (RAL8022) rhizomorphs.

Basidiospores (4.7) 5–6.4 (6.6) × (3) 3.1–4.15 (4.5) µm, *Q* = (1.11) 1.25–1.78 (2.07) [1.5 ± 0.18], ellipsoid, slightly thick-walled, nearly hyaline, inamyloid. Basidia 19–25 × 5–6.3 µm, clavate, smooth, thin-walled, hyaline, 4- (rarely 2-) sterigmate. Basidioles 17–24 × 5–6.6 µm, clavate, thin-walled, hyaline. Cheilocystidia abundant, thin-walled, hyaline; main body clavate, 10–22 × 3–7.5 µm, usually with two slender cylindrical, finger-like branches at apex, branches 7–14 × 1.7–3.7 µm; some cheilocystidia with three branches, base 17–21 × 5–8.2 µm, branches 4.7–15 × 1.2–3.6 µm; occasionally with four branches. Pleurocystidia not observed. Lamellar trama regular; hyphae 4.5–6.7 µm wide, with clamp connections. Pileus hairs 503–627 × 3–5 µm, setose, thick-walled, septate, dextrinoid, deep brownish-brown. Pileus trama cutis-type; hyphae 6.2–9.8 µm wide, reddish-brown, thin-walled, unchanged in 5% KOH. Stipe hairs composed of cylindrical, dark brown, thick-walled hyphae 5–7.3 µm wide, with fusiform, ellipsoid, or subglobose swellings 35–48 × 16–30 µm; terminal hyphae subfusiform, septate, unchanged in 5% KOH. Clamp connections present in all tissues.

###### Typus.

CHINA • Jilin Province, Jilin City, Jiaohe City, Qianjin Forest Farm, on leaf litter in coniferous forests, 6 September 2024, 43°57'21"N, 127°42'53"E, alt. 409 m, T. Bau, FJAU69428 (ZTY249619), holotype.

###### Additional materials examined.

CHINA • Jilin Province, Baishan City, Fusong County, Ancient Tree Park, on leaf litter in coniferous forests, 20 August 2021, 42°27'48"N, 127°52'43"E, alt. 801 m, T. Bau & Q.Q. Dong, FJAU69452 (DQQ21). • Jilin Province, Baishan City, Fusong County, Lushuihe National Forest Park, growing on dead branches of broad-leaved trees, 42°29'38"N, 127°52'08"E, alt. 781 m, 8 August 2024, J. L. Wei, FJAU 69460 (J24080806). • Jilin Province, Baishan City, Fusong County, Songjianghe National Forest Park, on dead branches in coniferous forests, 42°10'23"N, 127°30'59"E, alt. 747 m, 10 August 2024, T.Y. Zhang, FJAU69461 (ZTY2481012). • Jilin City, Jiaohe City, Qianjin Forest Farm, on leaf litter in coniferous forests, 6 September 2024, 43°57'18"N, 127°42'44"E, alt. 408 m, Q.R. Liu, FJAU69430 (R24914). • Jilin City, Jiaohe City, Lafashan Mountain, on leaf litter in broad-leaved forests, 5 September 2024, 43°48'21"N, 127°23'50"E, alt. 349 m, T.Y. Zhang, FJAU69429 (ZTY249516).

###### Notes.

Kerekes and Desjardin (2009) indicated that the diagnostic taxonomic characteristics of *Crinipellis* species primarily include pileus color, the presence or absence of rhizomorphs, habitat type, cheilocystidial shape, the presence or absence of pleurocystidia, basidiospore shape and size, and color changes of pileipellis and stipitipellis hyphae in KOH solution. The key diagnostic features of the present species are as follows: a brown pileus surface covered with radially arranged dark brown tomentose scales; cream-colored lamellae; basidiospores measuring 4.7–6.4 × 3–4.5 µm; absent pleurocystidia; cheilocystidia bearing 2–4 digitate branches; and stipe hairs with fusiform or flask-shaped swellings that remain unchanged in 5% KOH. This species is morphologically similar to *Cr. rhizomaticola* Antonín, Ryoo & H.D. Shin, which also possesses a chestnut-brown to brown pileus covered with tomentose scales, and white to pale cream lamellae (Antonín et al. 2009). *Cr. rhizomaticola* differs by producing abundant pleurocystidia, clavate cheilocystidia with irregular, coarse branches at the apex, and stipitipellis hyphae that turn bright olive-green in 5% KOH. Phylogenetically, this species is most closely related to *Cr. zonata* (Peck) Pat., which has relatively small basidiospores (6.5–7.25 × 3.0–4.0 µm) within this genus. However, *Cr. zonata* exhibits zonate-radial pileal scale arrangement, whereas the present species has longitudinally and radially arranged scales without a zonate pattern (Patouillard 1900). Furthermore, *Cr. zonata* possesses clavate or subfusiform cheilocystidia with irregularly curved, rostrate apical projections, while the present species has cheilocystidia with slender bases and typically 2–4 long finger-like apical branches. In addition, the stipitipellis hyphae of the present species frequently form fusiform or flask-shaped swellings, which distinguishes it from *Cr. zonata* (Patouillard 1900; Antonín et al. 2009; Liu et al. 2018).

##### Moniliophthora vaginicola

Taxon classificationFungiAgaricalesMarasmiaceae

T. Bau & W.N. Hou sp. nov.

2E1EAE46-0290-518A-89E9-2EE7D42A2A15

864485

Figs 14J–N, 17

###### Diagnosis.

It is characterized by its cream pileus with a warm-brown center, flocculose-scaly stipe, and growth on withered *Poaceae* leaf sheaths.

**Figure 17.**
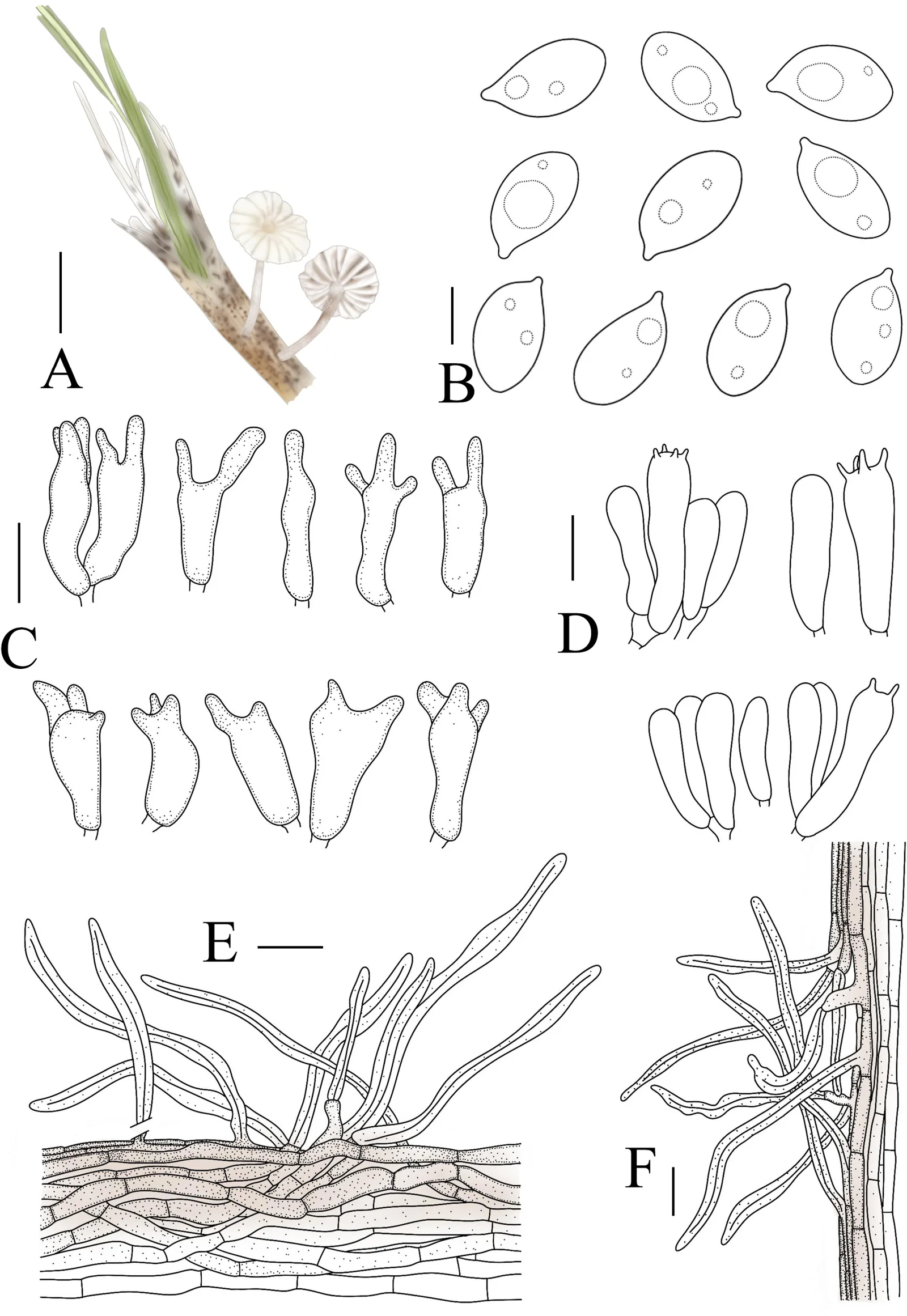
Microscopic structures of *Mo. vaginicola*. **A** Basidiomata. **B** Basidiospores. **C** Cheilocystidia. **D** Basidia and basidioles. **E** Pileipellis. **F** Stipitipellis. Scale bars: 0.5 cm **(A)**; 5 μm **(B)**; 15 μm **(C–F)**.

###### Etymology.

“vaginicola” refers to its growth on withered, yellowing leaf sheaths near the base of living *Poaceae* plants.

###### Description.

Basidiomata small to medium-sized. Pileus 0.5–1.2 cm diam., initially convex, becoming nearly plane, centrally depressed; surface creamy white to cream-colored (RAL9001), roughened; center warm brown (RAL8003); margin with deep, longitudinal, radial grooves. Context thin, beige (RAL1001). Lamellae adnate, relatively sparse, *L* = 13–16, I = 1, beige (RAL1001) with a pinkish tint. Stipe 1.4–1.8 cm long, 1–2 mm thick, cylindrical, slightly thicker at apex, hollow; apex beige (RAL1001) with a pinkish tint, middle clay brown (RAL8001), base warm brown (RAL8003); surface densely covered with cream-colored (RAL9001), flocculent, fine scales.

Basidiospores (8.4) 8.7–11 (12) × (5.1) 5.5–6.4 (6.7) µm, *Q* = (1.47) 1.48–1.93 (2.04) [1.66 ± 0.14], subellipsoid, thin-walled, hyaline, inamyloid. Basidia 35–41 × 8–10 µm, clavate, thin-walled, hyaline, inamyloid, 4- (rarely 2-) sterigmate; sterigmata 2.4–6.2 µm long. Basidioles 26–38 × 7–10 µm, clavate to cylindrical, smooth, thin-walled, hyaline, inamyloid. Cheilocystidia 16–29 × 5.8–11 µm, cylindrical, clavate to irregular, smooth, thin-walled, hyaline; apex bearing 1–3 digitate projections, projections 1.7–12 µm high. Pleurocystidia not observed. Lamellar trama irregular; hyphae 5–7 µm wide, thin-walled, hyaline, inamyloid. Pileus hairs 80–258 × 5.1–7.9 µm, setose, inamyloid, densely arranged, thick-walled to solid, apex slightly acute, sometimes bearing a single digitate projection. Pileus trama a cutis; hyphae 4–6.3 µm wide, slightly thick-walled, smooth, inamyloid, pale brown in water, unchanged in 5% KOH. Stipe hairs 58–145 × 4.5–7.1 µm, setose, thick-walled to solid, apex acute. Stipe trama composed of parallel, thick-walled, smooth hyphae 3.4–5.4 µm wide. Clamp connections present in all tissues.

###### Typus.

CHINA • Inner Mongolia Autonomous Region, Tongliao City, Horqin Left Back Banner, Wudantala Forest Farm, on yellowing leaf sheaths, *Poaceae* plants, 43°00'59"N, 122°44'36"E, alt. 217 m, 15 July 2023, T. Bau & W. N. Hou, FJAU69463 (H230758), holotype!.

###### Additional materials examined.

CHINA • Inner Mongolia Autonomous Region, Tongliao City, Hure Banner, on withered leaf sheaths, *Cyperaceae* plants, 42°45'47"N, 121°46'17"E, alt. 352 m, 17 July 2023, T. Bau & W. N. Hou, FJAU69464 (H2307166). • Inner Mongolia Autonomous Region, Tongliao City, Horqin Left Middle Banner, Wusitu Nature Reserve, on yellowing leaf sheaths, *Poaceae* plants, 43°47'47"N, 123°17'43"E, 148 m asl, 21 July 2023, T. Bau & W. N. Hou, FJAU69465 (H2307246). MONGOLIA • Dornod Province, Matad, on yellowing leaf sheaths, *Poaceae* plants. 47°40'09"N, 116°54'27"E, alt. 626 m, 23 December 2024, T. Bau & B. Ganbaatar, FJAU69435 (B23005). • Dornod Province, Matad, on withered, yellowing leaf sheaths near the base of living *Poaceae* plants, 47°40'09"N, 116°54'27"E, alt. 626 m, 23 December 2024, T. Bau & B. Ganbaatar, FJAU69434 (B23020).

###### Notes.

Phylogenetic analyses show that the GenBank sequence PQ732594, labeled as *P. palmivorus* (Sharples) Antonín & M. Kolařík and derived from specimen UBA23005, forms a well-supported independent clade with the examined specimens (PP = 1, UFBoot = 100%). Examination of specimen UBA23005, collected from Mongolia by Burenbaatar Ganbaatar, reveals that its morphological characters are consistent with those of the examined materials. Based on both phylogenetic evidence and morphological comparison, these specimens are therefore regarded as conspecific. Furthermore, the presence of a pileipellis with dense setae, together with its placement within the *Moniliophthora* clade (PP = 1, UFBoot = 99%), indicates that this taxon is better assigned to *Moniliophthora* rather than *Paramarasmius*.

Basidiomatal pileus color is variable and often relatively vivid among the 15 currently recognized species of *Moniliophthora*. To be specific, *Mo. pakistanica* Izhar, Asif, Niazi & Khalid and *Mo. ticoi* (Halling) Niveiro, N.A. Ramírez, Lodge & Aime exhibit orange tones, *Mo. mayarum* Lodge, Aime & Niveiro displays reddish tones, and *Mo. purpurea* Fatima & Khalid presents purplish tones (Izhar et al. 2022; Haqnawaz et al. 2024; Niveiro et al. 2020). The nearly cream-colored pileus surface of this species clearly differs from those of other known species in the genus. As indicated by the above findings, basidiomatal pigmentation may provide useful auxiliary evidence for species delimitation, although it should not be considered a shared diagnostic character of *Moniliophthora*.

The phylogenetic analyses involved sequence data from as many *Moniliophthora* species as possible. The resulting tree indicates that the recently described *Mo. sclerotium* is closely related to this species. Nevertheless, *Mo. sclerotium* is most readily distinguished by the production of subglobose, ellipsoid to irregular sclerotia, whereas sclerotia are absent in *Mo. vaginicola* (Zhang et al. 2025). At present, *Mo. sclerotium* usually occurs from scattered to gregarious on decaying wood and is known only from Yunnan Province.

##### Crinipellis xiaoxinganlingensis

Taxon classificationFungiAgaricalesMarasmiaceae

nom. prov.

D4341EB6-DDC3-5A6B-9834-9633A6C284B1

Figs 14G–I, 18

###### Etymology.

“xiaoxinganlingensis” refers to the type locality, Xiaoxinganling, the Lesser Khingan Range in Northeast China.

**Figure 18.**
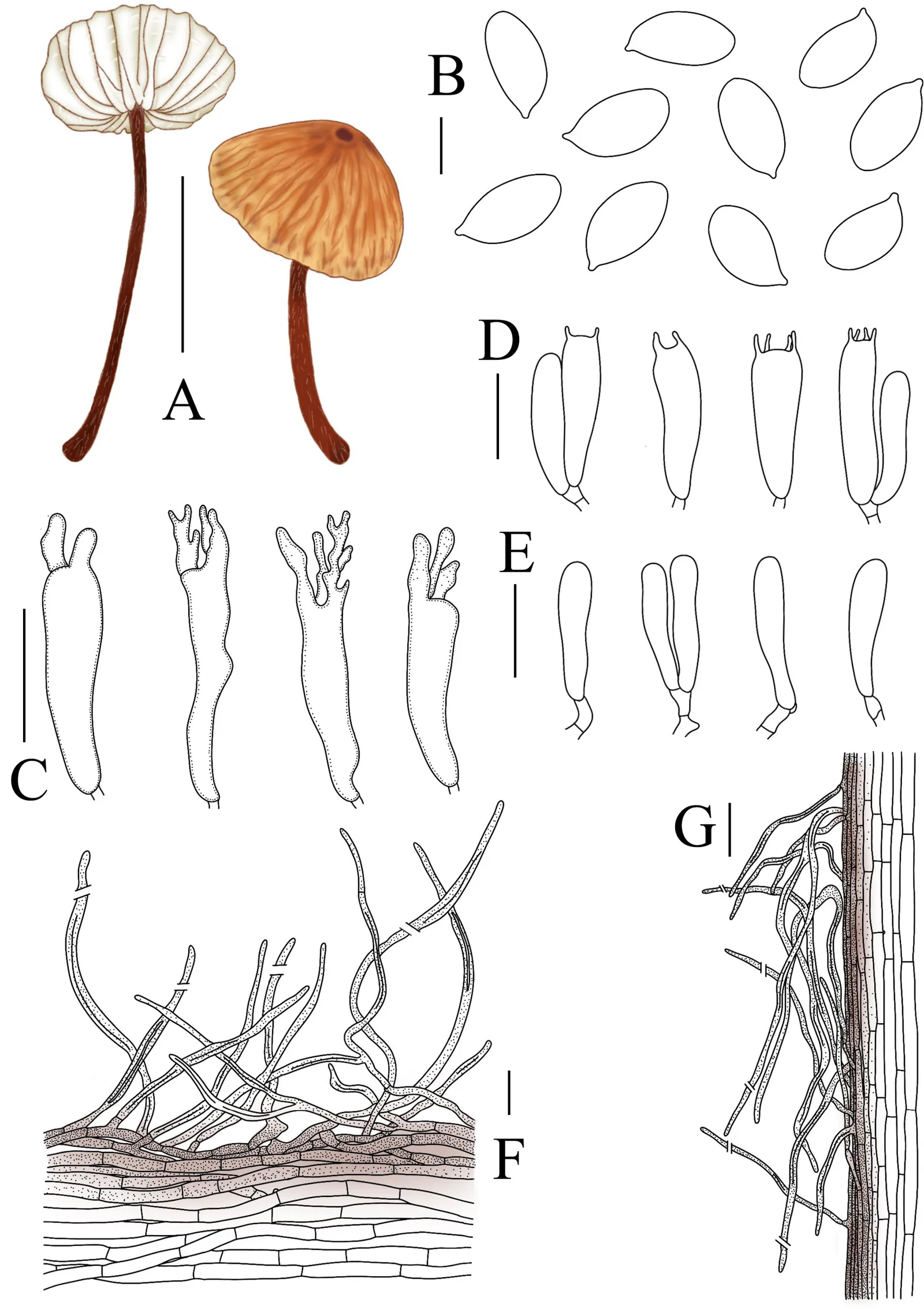
Microscopic structures of *Cr. xiaoxinganlingensis* nom. prov. **A** Basidiomata. **B** Basidiospores. **C** Cheilocystidia. **D** Basidia. **E** Basidioles. **F** Pileipellis. **G** Stipitipellis. Scale bars: 0.5 cm **(A)**; 5 μm **(B)**; 15 μm **(C–G)**.

###### Description.

Basidiomata small. Pileus 0.3–0.6 cm diam., convex to plane, slightly depressed at center; surface cream-yellow (RAL1034), covered with reddish-brown (RAL3011) to clay-brown (RAL8011), radially arranged tomentose scales; scales denser at center, dark copper-brown (RAL8004), becoming sparse toward margin. Context cream (RAL9001), thin; odor indistinct. Lamellae free, moderately dense, unequal, *L* = 20–26, *I* = 1–3, cream (RAL9001), bruising reddish-brown (RAL3011), but showing no immediate or only very faint color change when cut with a sharp knife; color unchanged when dry. Stipe 1.2–1.9 cm long, 1–3 mm thick, cylindrical, equal or slightly thicker at base, hollow, fibrous; surface clay-brown (RAL8001), rough, densely covered with reddish-brown (RAL3011) tomentose scales; base directly attached to substrate, with black rhizomorphs, but not arising from the rhizomorphs.

Basidiospores (6.2) 6.7–9.8 (9.9) × (3.7) 3.8–5.5 (5.6) µm, *Q* = (1.24) 1.36–2.1 (2.11) [1.72 ± 0.24], oblong-ellipsoid, smooth, thin-walled, hyaline, containing oil droplets, inamyloid. Basidia 23–29 × 6–9 µm, clavate, thin-walled, hyaline, inamyloid, 4- (rarely 2-) sterigmate. Basidioles 16–28 × 4.4–7.5 µm, clavate to cylindrical, hyaline, smooth, thin-walled, inamyloid. Cheilocystidia 19–26 × 6–9 µm, clavate, thick-walled, hyaline; apex bearing 2–4 digitate branches, branches 4–8 µm long. Pleurocystidia not observed. Lamellar trama subregular; hyphae 3–5 µm wide, thin-walled, hyaline, inamyloid. Pileus hairs 125–327 × 3–6 µm, setose, thick-walled, pale brown, dextrinoid. Pileus trama a cutis, inamyloid, brown in water, unchanged in 5% KOH. Stipe hairs 98–361 × 4–6 µm, setose, thick-walled. Stipe trama composed of cylindrical cells 3–5 µm wide, yellowish-brown with a faint greenish tint in 5% KOH. Clamp connections present.

###### Materials examined.

CHINA • Heilongjiang Province, Yichun City, Fenglin County, Wuying National Forest Park, scattered on dead twigs of shrubs, 26 July 2023, 48°14'10"N, 129°12'39"E, alt. 392 m, W.N. Hou & L.Y. Zhu, FJAU69447 (H2307344). • Heilongjiang Province, Yichun City, Fenglin County, Wuying National Forest Park, scattered on dead twigs of shrubs, 26 July 2023, 48°14'07"N, 129°12'51"E, alt. 390 m, W.N. Hou & L.Y. Zhu, FJAU69448 (H23073442).

###### Notes.

The morphological characters of *Cr. xiaoxinganlingensis* nom. prov. conform to the generic concept of *Crinipellis* established by Patouillard (Patouillard 1889; Kerekes and Desjardin 2009). Its diagnostic features, including a cream-yellow pileus covered with reddish-brown, radially arranged tomentose scales, clavate cheilocystidia, inamyloid lamellar trama, and a yellowish-brown stipitipellis with a faint greenish tint in 5% KOH, also match the supplementary characters proposed for *Crinipellis* by Kerekes and Desjardin (2009). On morphological grounds, the assignment of this species to *Crinipellis* is therefore reasonable. Phylogenetic evidence, however, complicates its generic placement. Although the two specimens form a well-supported clade outside the main *Crinipellis* clade, their morphological characters are consistent with those of *Crinipellis*, leaving their generic placement uncertain.

During field observations, *Cr. xiaoxinganlingensis* nom. prov. exhibited a distinct color change in the lamellae upon bruising. Despite this prominent diagnostic character and its unusual phylogenetic position (Fig. 1), the species is not formally described as new at this stage. This decision is based primarily on two considerations. First, only two collections originating from the same geographic locality have been examined to date, which is insufficient for evaluating the full range of morphological variation within the taxon. Second, the generic placement of *Cr. xiaoxinganlingensis* nom. prov. remains unresolved. Prematurely publishing it as a new species of *Crinipellis* could further complicate genus circumscription and render the current generic circumscription non-monophyletic. Phylogenetic analyses also indicate that the clade containing this taxon may constitute an independent genus-level clade. Even so, erecting a new genus is not appropriate at present for several reasons. A clear suite of shared generic characters cannot yet be defined for this lineage. Beyond the bruising reaction and its unique habitat, no additional key morphological characters have been identified that clearly separate it from closely related genera. These two features alone are insufficient to justify the recognition of a new genus. It also remains uncertain whether the discovery of this taxon points toward a broader systematic range for *Crinipellis*.

Accordingly, a detailed description, illustrations of diagnostic characters, and sequence data are provided herein for *Crinipellis xiaoxinganlingensis* nom. prov. These data aim to support future collections and comparative studies of taxa occupying similar phylogenetic positions and may help achieve a more robust resolution of this taxonomic problem. *Cr. xiaoxinganlingensis* nom. prov. is currently known only from the Lesser Khingan Mountains in Northeast China. The Lesser Khingan region lies within the mid-temperate continental monsoon climate zone and features mixed coniferous-broad-leaved forests (e.g., well-preserved primary *Pinus koraiensis* Siebold & Zucc. forests) (Sun et al. 2026). A distinctive forest-floor microenvironment may be formed by the cold climate, seasonally frozen ground, dense canopy, and slowly decomposing litter layer (Zhang and Jia 2023).

## Discussion

### Morphology and phylogenetic analyses

In this study, the reconstructed phylogenetic framework of *Marasmiaceae* can support the conclusions drawn by Oliveira et al. (2024a). As indicated by the findings of this study, *Crinipellis* forms a sister clade to the clade with *Moniliophthora* and *Paramarasmius*, and the latter two genera show a close correlation, and *Marasmius* is recovered as a monophyletic group. The nomenclature for the two subfamilies within *Marasmiaceae* is also corrected herein, namely *Marasmioideae* and *Crinipelloideae*. Although these genera were established or circumscribed based on defined morphological characters, substantial morphological overlap and taxonomic confusion persist among closely related genera. The inclusion of *Cr. xiaoxinganlingensis* nom. prov. furnishes new evidence for interpreting relationships among these genera. Historically, numerous species originally assigned to *Crinipellis* have been recombined into *Chaetocalathus* or *Moniliophthora*, such as *Ch. asperifolius* (Pat.) Singer (= *Cr. asperifolia* Pat.), *Mo. canescens* (Har. Takah.) Kerekes & Desjardin (= *Cr. canescens* Har. Takah.), and *Mo. conchata* (Har. Takah.) Antonín, Ryoo & Ka (= *Cr. conchata* Har. Takah.). These taxonomic revisions have complicated morphological delimitation within *Crinipellis*. From a morphological perspective, the discovery of *Cr. xiaoxinganlingensis* nom. prov. is of great significance. Its basidiomata display a distinct bruising discoloration reaction when squeezed or injured, a feature that has been rarely recorded in *Marasmiaceae*. The above-mentioned reaction may be linked to oxidative enzyme activity and may indicate adaptive strategies against insect herbivory or microbial competition. This character reveals that the morphological diversity of *Crinipellis* may be far greater than presently recognized and that additional discoveries will likely yield valuable insights into the evolution and delimitation of this genus.

Recent multigene phylogenetic research has revised the classification of *Marasmius*, restricted its circumscription, and recognized at least two subgenera, namely subgen. *Globulares* and subgen. *Marasmius* (Oliveira et al. 2024a). Phylogenetic analyses of *Marasmius* subg. *Globulares* and *Marasmius* subg. *Marasmius* were performed using combined ITS, LSU, *rpb*2, and *tef*1-α sequence data. Within *Marasmius* subg. *Globulares*, the clades corresponding to sect. *Sicci* and sect. *Globulares* received strong phylogenetic support. Morphologically, the two sections are primarily distinguished by the presence or absence of pleurocystidia and by the occurrence of refractive elements. Compared with sect. *Sicci*, the infrasectional classification of sect. *Globulares* is considerably more complex and comprises both subsection- and series-level taxa. Delimitation of these subordinate groups frequently requires careful examination of multiple morphological characters, including lamellar attachment, as well as the morphology of pleurocystidia and caulocystidia (Oliveira et al. 2024a; Guard et al. 2026). Within *Marasmius* subg. *Marasmius*, species of subsect. *Marasmius* possess a pileipellis composed of typical *Rotalis*-type cells. This contrasts with the closely related sect. *Guyanenses*, in which *Siccus*-type cells predominate and only a few smooth cells are occasionally intermixed.

### Ecology and distribution

Regarding habitat preference, *Crinipellis* exhibits a comparatively broad habitat spectrum, with species occurring on broadleaf, herbaceous, and coniferous substrates (Bandala et al. 2012; Antonín 2012; Niveiro et al. 2020). Most species are saprotrophic, reflecting a high degree of ecological adaptability. Notably, *Moniliophthora* and *Paramarasmius* show a stronger preference for broadleaf and herbaceous substrates. In general, their species occur as plant pathogens, thereby distinguishing them ecologically from *Crinipellis* (Antonín et al. 2022; Capelari et al. 2010; Haqnawaz et al. 2024). Nevertheless, *Moniliophthora* and *Paramarasmius* display a high degree of similarity in habitat selection. Morphologically, some species of *Moniliophthora* (e.g., *Mo. mayarum*) possess sparse pileal squamules, a feature that may represent an intermediate state toward the characteristic morphology observed in *Paramarasmius* (Antonín 2012; Niveiro et al. 2020; Oliveira et al. 2024a). As indicated by existing research, *Moniliophthora* and *Paramarasmius* constitute a single evolutionary clade (Oliveira et al. 2024a). The occurrence of *Cr. xiaoxinganlingensis* on shrub branches may reflect a specialized substrate preference. In the forest understory of this region, low light availability, a thick litter layer, and slow decomposition may limit suitable microhabitats at ground level. By contrast, shrub branches provide a relatively elevated and well-aerated substrate, potentially reducing exposure to strong seasonal freeze–thaw disturbance near the forest floor. This interpretation, based on limited field observations, remains preliminary. If confirmed by repeated collections and additional ecological evidence, this substrate preference may provide further insights into the ecological differentiation and adaptive evolution of this lineage. In addition, the recently described species *Me. coprophila* Burenb. & T. Bau, which occurs on sheep dung, represents an exceptionally rare ecological niche (Hou et al. 2024). Although this species is retained within *Marasmiaceae*, the obtained phylogenetic results provide support for its placement within *Campanellaceae*, consistent with its distinct evolutionary position and ecological characteristics (Petersen and Hughes 2024).

The marasmioid fungi of the Northeast China Ecological Region are dominated by temperate elements and exhibit biogeographical affinities with the northern temperate regions of Europe and North America. A total of 10 new species were discovered and described. On that basis, the known species diversity of marasmioid fungi in the Northeast China Ecological Region has been substantially enriched. To date, approximately 75 species of marasmioid fungi have been recorded from this region (Li 1991, 1998; Li and Bau 2003; Zhang 2014; Bau et al. 2017; Liu and Li 2019; Bau 2019; Bao et al. 2021; Guo et al. 2023; Bau 2024; Jiang et al. 2024).

## Supplementary Material

XML Treatment for Marasmius jilinensis

XML Treatment for Marasmius horqinensis

XML Treatment for Marasmius unicuspidatus

XML Treatment for Marasmius variicystidiatus

XML Treatment for Marasmius daqinggouensis

XML Treatment for Marasmius coronatospinosus

XML Treatment for Marasmius clavocystidiatus

XML Treatment for Marasmius maoershanensis

XML Treatment for Crinipellis macrospora

XML Treatment for Crinipellis fuscosquamosa

XML Treatment for Moniliophthora vaginicola

XML Treatment for Crinipellis xiaoxinganlingensis

## Additional information

### Artificial Intelligence (AI) use

The authors accept full responsibility for the content of the manuscript, including the disclosure of any use of AI.

No AI tools were used in the preparation of this manuscript.

### Adherence to national and international regulations

All the fungal strains used in this study have been legally obtained, respecting the Convention on Biological Diversity (Rio Convention).

### Author ORCIDs

W.-N. Hou https://orcid.org/0000-0002-9838-3489

T. Bau https://orcid.org/0000-0003-2461-9345

### Data availability

All the sequences have been deposited in GenBank (https://www.ncbi.nlm.nih.gov). Fungal name registrations have been submitted to either Index Fungorum(https://www.indexfungorum.org) or Mycobank (https://www.mycobank.org). The data presented in this study are deposited in the Zenodo repository https://doi.org/10.5281/zenodo.21225752. The reviewer access URL is https://zenodo.org/records/21225752?preview=1&token=eyJhbGciOiJIUzUxMiJ9.eyJpZCI6IjE4MDc3ZGJiLTA1NmQtNGJhMi05MDE1LWFkNzBkYmVlZmVlYSIsImRhdGEiOnt9LCJyYW5kb20iOiI0NmMxMGE2NTY5MjZmMWQxNTQ4NDlmNTAzYmNmNTRhOCJ9.QWqqihN3OtVHCUVCz6KE89KevfFzxy6ajWfwxAMI0beZ1jDDDCnB4N7c_yfzGmrr4_gU-mZlt6mEOZaoRuJlVQ.
